# MEK1/2 inhibitor withdrawal reverses acquired resistance driven by BRAF^V600E^ amplification whereas KRAS^G13D^ amplification promotes EMT-chemoresistance

**DOI:** 10.1038/s41467-019-09438-w

**Published:** 2019-05-02

**Authors:** Matthew J. Sale, Kathryn Balmanno, Jayeta Saxena, Eiko Ozono, Katarzyna Wojdyla, Rebecca E. McIntyre, Rebecca Gilley, Anna Woroniuk, Karen D. Howarth, Gareth Hughes, Jonathan R. Dry, Mark J. Arends, Pilar Caro, David Oxley, Susan Ashton, David J. Adams, Julio Saez-Rodriguez, Paul D. Smith, Simon J. Cook

**Affiliations:** 1 Signalling Programme, The Babraham Institute, Babraham Research Campus, Cambridge, CB22 3AT UK; 2 Proteomics Facility, The Babraham Institute, Babraham Research Campus, Cambridge, CB22 3AT UK; 3 Experimental Cancer Genetics, Wellcome Sanger Institute, Wellcome Genome Campus, Hinxton, Cambridgeshire CB10 1SA UK; 4 Hutchison-MRC Research Centre, Department of Pathology, University of Cambridge, Hills Road, Cambridge, CB2 0XZ UK; 5 Oncology Bioscience, Innovative Medicines and Early Development Biotech Unit, AstraZeneca, CRUK Cambridge Institute, Robinson Way, Cambridge, CB2 0RE UK; 6 Oncology Bioscience, Innovative Medicines and Early Development Biotech Unit, AstraZeneca, 35 Gatehouse Drive, Waltham, MA 02451 USA; 7 Division of Pathology, Centre for Comparative Pathology, Cancer Research UK Edinburgh Centre, MRC Institute of Genetics and Molecular Medicine, University of Edinburgh, Western General Hospital, Crewe Road South, Edinburgh, EH4 2XR UK; 8 Oncology Bioscience, Innovative Medicines and Early Development Biotech Unit, AstraZeneca, Alderley Park, Macclesfield, SK10 4TG UK; 9 European Molecular Biology Laboratory, European Bioinformatics Institute (EMBL-EBI), Wellcome Genome Campus, Hinxton, Cambridgeshire CB10 1SD UK

**Keywords:** Kinases, Cancer therapeutic resistance, Growth factor signalling

## Abstract

Acquired resistance to MEK1/2 inhibitors (MEKi) arises through amplification of BRAF^V600E^ or KRAS^G13D^ to reinstate ERK1/2 signalling. Here we show that BRAF^V600E^ amplification and MEKi resistance are reversible following drug withdrawal. Cells with BRAF^V600E^ amplification are addicted to MEKi to maintain a precise level of ERK1/2 signalling that is optimal for cell proliferation and survival, and tumour growth in vivo. Robust ERK1/2 activation following MEKi withdrawal drives a p57^KIP2^-dependent G1 cell cycle arrest and senescence or expression of NOXA and cell death, selecting against those cells with amplified BRAF^V600E^. p57^KIP2^ expression is required for loss of BRAF^V600E^ amplification and reversal of MEKi resistance. Thus, BRAF^V600E^ amplification confers a selective disadvantage during drug withdrawal, validating intermittent dosing to forestall resistance. In contrast, resistance driven by KRAS^G13D^ amplification is not reversible; rather ERK1/2 hyperactivation drives ZEB1-dependent epithelial-to-mesenchymal transition and chemoresistance, arguing strongly against the use of drug holidays in cases of KRAS^G13D^ amplification.

## Abstract (web summary)

Colorectal cancer cells can acquire resistance to MEK inhibition due to BRAF or KRAS amplification. Here, the authors show that while MEK inhibitor withdrawal in BRAF mutant cells restores sensitivity to the inhibitor through the loss of BRAF amplification mediated by a p57-dependent mechanism, drug withdrawal from KRAS mutant cells does not restore sensitivity but results in EMT and chemoresistance.

## Introduction

Tumour cells with BRAF (most frequently BRAF^V600E^) or KRAS mutations are addicted to MEK1/2 (MAPK or ERK Kinase)–ERK1/2 (extracellular signal-regulated kinase) signalling for their proliferation, survival and other malignant properties. BRAF inhibitors (BRAFis) and allosteric MEK1/2 inhibitors (MEKis) are effective against BRAF^V600E^-positive melanoma^1,2^ and are approved for the treatment of melanoma; indeed, BRAFi + MEKi combination is now the front line treatment for BRAF^V600E^-mutant melanoma^3,4^. RAF inhibitors are ineffective in RAS^Mut^ tumours since they drive paradoxical ERK1/2 pathway activation and adventitious tumour development^1,5^. MEKi do not exhibit this limitation but relief of feedback inhibition and pathway reactivation limits MEKi monotherapy in RAS^Mut^-driven tumours^2,6^.

Although BRAFi^7,8^, MEKi^9^ and BRAFi + MEKi^3,4^ improve progression-free and overall survival, clinical responses are often short lived, due to the emergence of acquired resistance, which typically maintains or reactivates the ERK1/2 pathway in the presence of drug^1,2,10^. Resistance to BRAFi involves mechanisms that reactivate MEK1/2 including: amplification of *BRAF*^*T1799A*^ (hereafter referred to as BRAF^V600E^ amplification)^11^; emergence of BRAF^V600E^ splice variants^12;^ alternative MEK1/2 activators^13;^ NRAS or RTK upregulation and emergent MEK1 or NRAS mutations^14,15^. Mechanisms of acquired resistance to MEKi include: mutations in MEK1 that prevent drug binding or enhance kinase activity^15–18;^ BRAF^V600E^ amplification^19,20^ or *KRAS*^*G38A*^ amplification (hereafter referred to as KRAS^G13D^ amplification)^17,20^.

We previously demonstrated that colorectal cancer cells acquire resistance to the MEKi selumetinib (AZD6244/ARRY-142886) through amplification of BRAF^V600E^ or KRAS^G13D^ ^20^. We now show that selumetinib resistance driven by BRAF^V600E^ amplification is completely reversible upon prolonged drug withdrawal because BRAF^V600E^ amplification confers a selective disadvantage in the absence of MEKi. MEKi withdrawal drives ERK1/2 activation beyond a critical sweet spot that is optimal for cell viability and proliferation. This drives a p57^KIP2^-dependent G1 cell cycle arrest and senescence or expression of the pro-apototic protein NOXA and cell death; these terminal responses select against cells with BRAF^V600E^ amplification, thereby driving reversal of resistance. Remarkably, MEKi resistance driven by KRAS^G13D^ amplification is not reversible; these cells do not exhibit growth defects upon MEKi withdrawal but undergo an ERK1/2-dependent epithelial-to-mesenchymal transition (EMT) and exhibit resistance to commonly used chemotherapeutics. Thus, the emergence of drug-addicted, MEKi-resistant cells, and the opportunity this may afford for intermittent dosing schedules (drug holidays), may be determined by the nature of the amplified driving oncogene (BRAF^V600E^ vs. KRAS^G13D^) further underscoring the difficulties of targeting KRAS mutant tumour cells.

## Results

### BRAF^V600E^ amplification and MEKi resistance are reversible

**Fig. 1:**
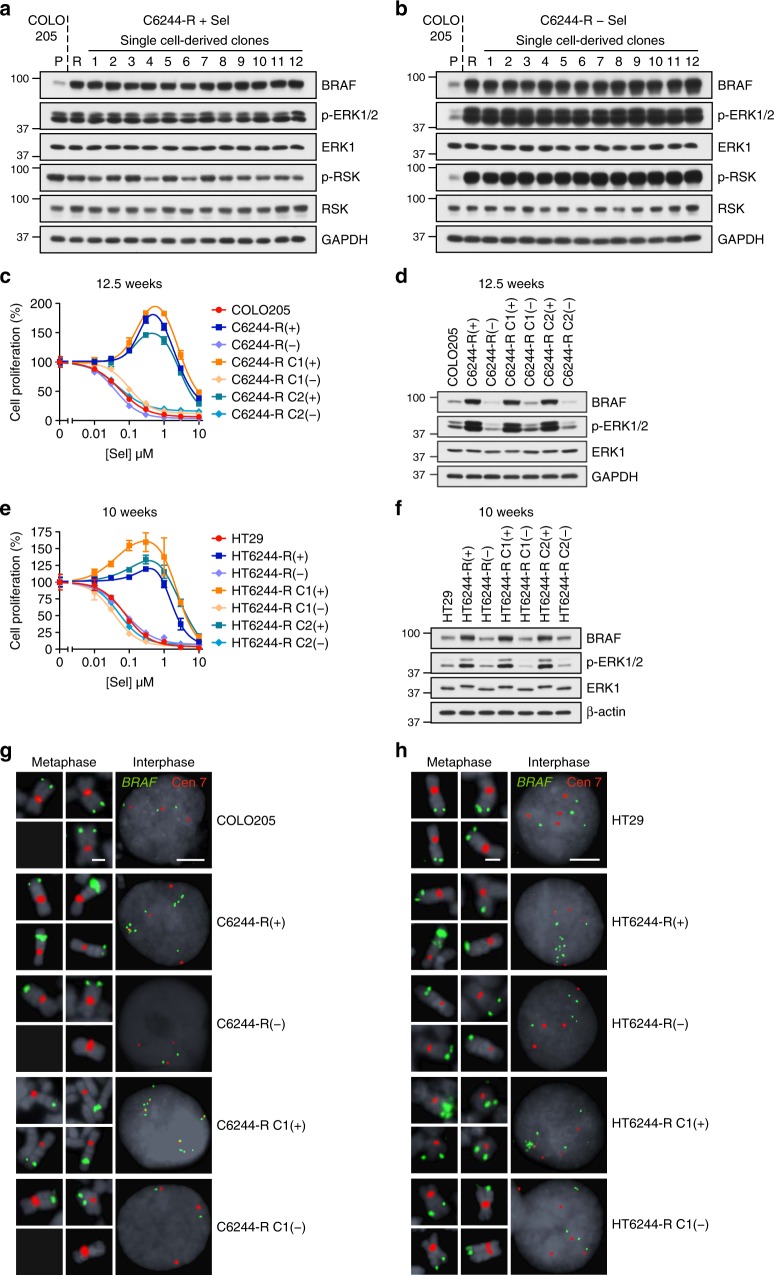
*BRAF* amplification is reversible in cells with acquired resistance to MEKi. **a**, **b** Non-clonal COLO205 cells with acquired resistance to selumetinib (C6244-R cells, R) and 12 single-cell clone derivatives of C6244-R (1–12) were treated with 1 μM selumetinib (Sel) (**a**) or selumetinib-free medium (**b**) for 24 h. Parental COLO205 cells (P) were treated in parallel with selumetinib-free medium for 24 h. Lysates were western blotted with the indicated antibodies. **c**, **d** Following 12.5 weeks culture in the presence (+) or absence (COLO205 and (−)) of 1 μM selumetinib, cells were treated with the indicated concentrations (10 nM to 10 μM) of selumetinib (Sel) for 24 h, and DNA synthesis assayed by [^3^H]thymidine incorporation (**c**), or incubated in selumetinib-free medium for 24 h and lysates western blotted with the indicated antibodies (**d**). C6244-R C1 and C6244-R C2 are single-cell clonal derivatives of C6244-R. Results (**c**) are mean ± SD of cell culture triplicates and normalized to control for each cell line. **e**, **f** Following 10 weeks culture in the presence (+) or absence (HT29 and (−)) of 1 μM selumetinib, cells were treated with the indicated concentrations (10 nM to 10 μM) of selumetinib (Sel) for 24 h, and DNA synthesis assayed by [^3^H]thymidine incorporation (**e**), or incubated in selumetinib-free medium for 24 h and lysates western blotted with the indicated antibodies (**f**). HT6244-R C1 and HT6244-R C2 are single-cell clone derivative cell lines of HT6244-R. Results (**e**) are mean ± SD of cell culture triplicates and normalized to control for each cell line. **g**, **h** Following 20 weeks culture in the presence (+) or absence (COLO205 and (−)) of 1 μM selumetinib (**g**), or 10 weeks culture in the presence (+) or absence (HT29 and (−)) of 1 μM selumetinib (**h**), *BRAF* locus PAC DNA (RP5-1173P7; green) and chromosome 7 centromere probe (red) were hybridized to metaphase spreads and interphase nuclei (grey, DAPI). C1 and C2 are single-cell clonal derivatives of C6244-R or HT6244-R as indicated. Scale bars indicate 2 µm (metaphase panels) and 10 µm (interphase panels)

BRAF^V600E^-mutant COLO205 and HT29 cells (Supplementary Table 1) adapt to MEK1/2 inhibition by amplifying BRAF^V600E^ to maintain ERK1/2 signalling in the presence of selumetinib^20^. For example, all single-cell clones derived from selumetinib-resistant COLO205 cells (C6244-R cells) exhibited elevated BRAF expression and normal, parental levels of active phosphorylated ERK1/2 (p-ERK1/2) in the presence of drug (Fig. 1a). This is because selumetinib does not block the activating phosphorylation of MEK1/2 by BRAF^V600E^ but constrains p-MEK1/2 in an inactive conformation; indeed, withdrawal of selumetinib for 24 h drove hyperactivation of ERK1/2 (Fig. 1b). When non-clonal C6244-R cells or two clonal lines (C6244-R C1 and C2) were cultured in the absence of selumetinib, resensitization was apparent after just 2.5 weeks (Supplementary Fig. 1a). By 12.5 weeks, cells reverted to full selumetinib sensitivity (Fig. 1c) with BRAF expression and p-ERK1/2 levels re-set to parental, drug-naive levels (Fig. 1d; Supplementary Fig. 1b). All clones derived from selumetinib-resistant HT29 cells also exhibited increased BRAF expression, normal MEKi-restrained levels of p-ERK1/2 and ERK1/2 hyperactivation after drug withdrawal (Supplementary Fig. 2a, b). Selumetinib resistance was also reversed by 10 weeks of drug withdrawal in HT6244-R and HT6244-R C1 and C2 clonal cell lines (Fig. 1e; Supplementary Fig. 2c) and BRAF expression and p-ERK1/2 levels were re-set to parental levels (Fig. 1f; Supplementary Fig. 2d).

Fluorescence in situ hybridization (FISH) revealed that COLO205 cells had three copies of chromosome 7, each with a single *BRAF* signal, whereas C6244-R(+) cells (maintained in selumetinib) had four copies of chromosome 7 and around 10 *BRAF* signals due to a focal intrachromosomal amplification (Fig. 1g). We failed to detect *BRAF* amplification in any revertant C6244-R(−) cells (non-clonal or single-cell-derived clones) that had been withdrawn from selumetinib selection (Fig. 1g). Intriguingly, both C6244-R(−) and C6244-R C1(−) cells reverted to a *BRAF* copy number of two despite having three copies of chromosome 7. Given that the C6244-R C1(+) clone harboured four copies of chromosome 7, two with a *BRAF* amplicon, this suggests that an entire copy of chromosome 7 with amplified *BRAF* was lost, whereas the *BRAF* amplicon was fully removed from the other to yield a chromosome 7 with no copies of *BRAF*. HT6244-R(+) and clonal derivative HT6244-R C1(+) cells also exhibited an intrachromosomal *BRAF* amplification on one of four copies of chromosome 7, amounting to ~ 12 copies of *BRAF* in total; this was absent in parental HT29 cells (four copies of *BRAF*). This amplification was lost in the revertant HT6244-R(−) cells: the number of copies of chromosome 7 was maintained (at 4) in revertant cells but they exhibited five copies of *BRAF* in total, again suggesting that the amplicon had been removed from its parent chromosome (Fig. 1h). Thus, BRAF^V600E^ amplification, which is selected for and confers selumetinib resistance^20^, was selected against and lost when cells were deprived of selumetinib.

### MEKi withdrawal from cells with amplified BRAF^V600E^ drives cell cycle arrest or death

**Fig. 2:**
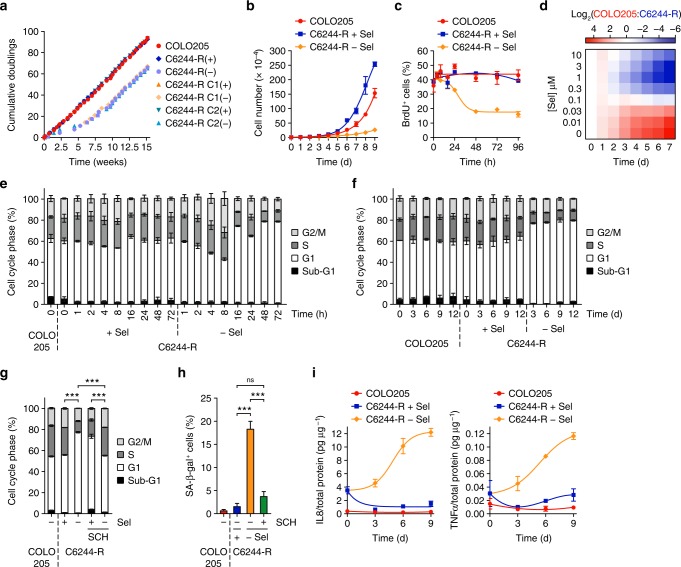
MEKi withdrawal from BRAF^V600E^-amplified MEKi-resistant COLO205 cells causes cell cycle arrest and senescence. **a** Cumulative doubling tallies for cells growing in the presence (+) or absence (COLO205 and (−)) of selumetinib over 15 weeks. **b** COLO205 and C6244-R cells were treated with 1 μM selumetinib (C6244-R + Sel) or DMSO only (COLO205, C6244-R − Sel) and cell numbers counted over 9 days. Results are mean ± SD of cell culture triplicates, representative of two experiments. **c** COLO205 and C6244-R cells were washed and treated with 1 μM selumetinib (C6244-R + Sel) or DMSO only (COLO205, C6244-R − Sel) and bromodeoxyuridine (BrdU) incorporation assayed at the indicated time points using flow cytometry. **d** COLO205 and C6244-R cells were independently labelled with distinct cell membrane dyes, mixed 1:1 and treated as indicated with selumetinib for 7 days. COLO205:C6244-R cell number ratio was determined daily by flow cytometry. Results are log_2_(mean COLO205:C6244-R cell number ratio) of four independent experiments. **e**, **f** COLO205 and C6244-R cells were treated with either 1 μM selumetinib (C6244-R + Sel) or DMSO only (COLO205, C6244-R − Sel) for the indicated times, and cell cycle distribution determined by flow cytometry. **g** COLO205 or C6244-R cells were treated with 1 μM selumetinib (Sel; +) or DMSO only (−), with or without 30 nM SCH772984 (SCH) for 72 h, and cell cycle distribution determined by flow cytometry. **h** COLO205 and C6244-R cells were treated with 1 μM selumetinib (C6244-R + Sel) or DMSO only (COLO205, C6244-R − Sel) for 9 days, after which the number of senescence-associated β-galactosidase-positive (SA-β-gal^+^) cells was scored. **i** C6244-R cells were treated with 1 μM selumetinib (C6244-R + Sel) or DMSO only (C6244-R − Sel) for up to 9 days, after which IL8 (left) or TNFα (right) levels in the culture medium were determined by ELISA. IL8 and TNFα levels were normalized to total cellular protein. **c**–**i** Results are mean of at least three (**c**–**h**) or two (**i**) independent experiments and error bars indicate SD. *P* < 0.001 (***) or *P* > 0.05 (ns) determined by one-way ANOVA with Tukey’s multiple comparisons test of G1 fractions (**g**) or SA-β-gal-positive cells (**h**)

Intrachromosomal gene amplifications tend to be more stable than extrachromosomal amplifications following release from drug selection^21^ so we examined the cellular response to selumetinib withdrawal. Resistant lines deprived of selumetinib (C6244-R(−)) went through a 7-week proliferative crisis before resuming proliferation at a similar rate to COLO205 or C6244-R(+) cells maintained in selumetinib (Fig. 2a). This coincided with the loss of BRAF expression that was apparent within 7.5 weeks (Supplementary Fig. 1b). At early time points of drug withdrawal C6244-R(−) cells exhibited a striking reduction in proliferation rate (Fig. 2b), including a 60% reduction in bromodeoxyuridine (BrdU) labelling (Fig. 2c), indicating they had become addicted to selumetinib for proliferation. Co-culture of distinctly labelled COLO205 and C6244-R cells in the presence of 0–10 µM selumetinib over 7 days revealed a ~ 30-fold enrichment of COLO205 cells in the absence of selumetinib indicating that BRAF^V600E^ amplification did indeed confer a fitness deficit in the absence of selumetinib (Fig. 2d). Increasing the selumetinib concentration resulted in a 100-fold enrichment of C6244-R in 1 µM selumetinib (Fig. 2d). C6244-R(−) cells progressed through the cell cycle for the first 8 h of drug withdrawal but underwent a G1 cell cycle arrest from 16 h that was sustained for 72 h (Fig. 2e) and at least 12 days (Fig. 2f). The ERK1/2 inhibitor SCH772984 prevented this G1 arrest, confirming dependence on ERK1/2 (Fig. 2g). Selumetinib withdrawal for 9 days also drove senescence of C6244-R cells as judged by ERK1/2-dependent senescence-associated beta-galactosidase (SA-β-gal) staining (Fig. 2h) and the secretion of senescence-associated secretory phenotype (SASP) cytokines (Fig. 2i)^22^.

**Fig. 3:**
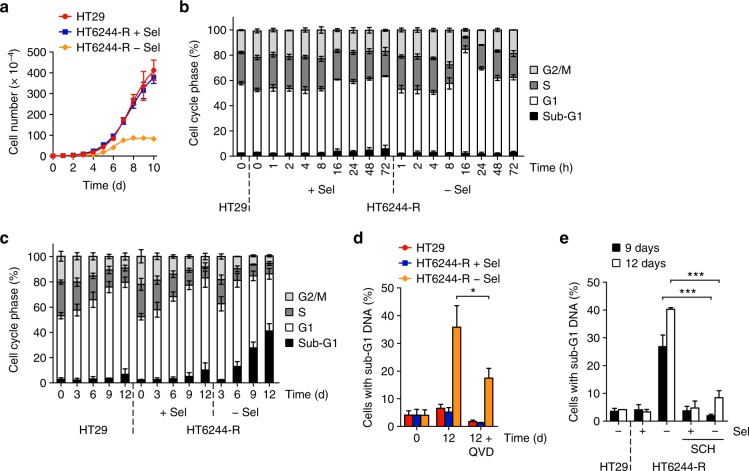
MEKi withdrawal from BRAF^V600E^-amplified MEKi-resistant HT29 cells causes cell death. **a** HT29 and HT6244-R cells were treated with either 1 μM selumetinib (HT6244-R + Sel) or DMSO only (HT29, HT6244-R − Sel) and cell numbers counted over 10 days. Results are mean ± SD of cell culture triplicates, representative of two experiments. **b**, **c** HT29 and HT6244-R cells were treated with either 1 μM selumetinib (HT6244-R + Sel) or DMSO only (HT6244-R − Sel) for the indicated times, and cell cycle distribution determined by flow cytometry. **d** HT29 and HT6244-R cells were treated with 1 μM selumetinib (HT6244-R + Sel) or DMSO only (HT29, HT6244-R − Sel), with or without 10 μM Q-VD-OPh (Q-VD) for up to 12 days, and sub-G1 fraction determined by flow cytometry. *P* < 0.05 (*) determined by unpaired two-tailed *t*-test. **e** HT29 or HT6244-R cells were treated with 1 μM selumetinib (Sel; +) or DMSO only (−), with or without 0.1 μM SCH772984 (SCH) for 9 or 12 days, and sub-G1 fraction determined by flow cytometry. *P* < 0.001 (***) determined by one-way ANOVA with Tukey’s multiple comparisons test. **b**–**e** Results are mean ± SD of three independent experiments

HT6244-R cells also exhibited a proliferation defect upon withdrawal of selumetinib (Fig. 3a). These cells underwent a transient G1 arrest after 16 h (Fig. 3b) but then proceeded to die from 6 days onwards as judged by their detachment from the culture surface (Supplementary Fig. 2e) and an increase in the fraction of cells exhibiting sub-G1 DNA (Fig. 3c). This cell death was reduced by the pan-caspase inhibitor Q-VD*-*OPH (Fig. 3d), and could be prevented by direct ERK1/2 inhibition (Fig. 3e). Thus, selumetinib-resistant cells with BRAF^V600E^ amplification underwent a G1 cell cycle arrest and senescence (C6244-R cells) or apoptosis (HT6244-R cells) when deprived of selumetinib, and these responses were ERK1/2 dependent.

### ERK1/2-dependent expression of p57^KIP2^ drives reversal of MEKi resistance

**Fig. 4:**
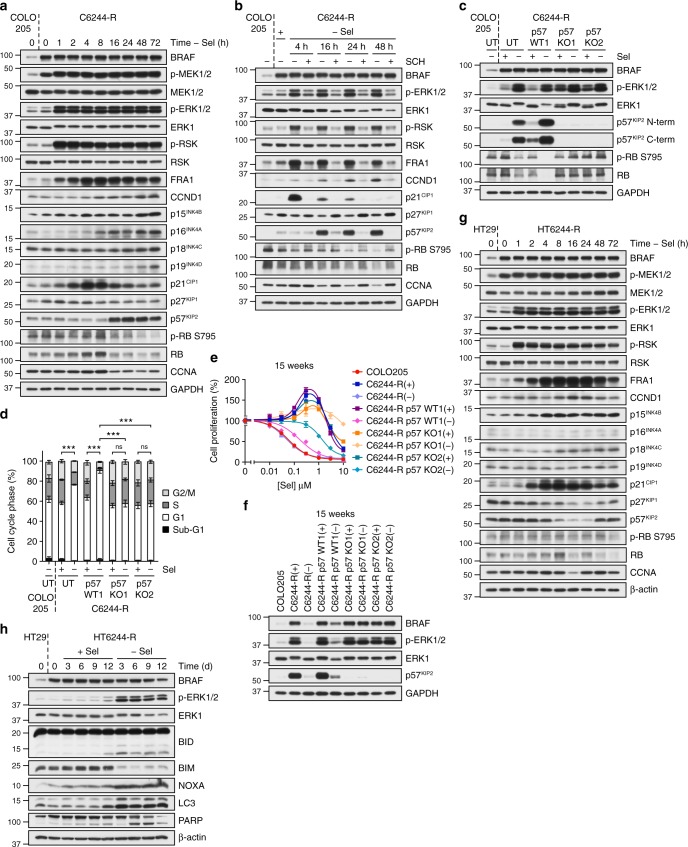
p57^KIP2^ is required for cell cycle arrest and reversal of resistance upon MEKi withdrawal from C6244-R cells. **a** C6244-R cells were treated with drug-free media (− Sel) for the indicated times (0–72 h), and compared to COLO205 cells at 0 h. Lysates western blotted with the indicated antibodies. **b** C6244-R cells were treated with drug-free media (− Sel) or 30 nM SCH772984 (SCH) for the indicated times. COLO205, and C6244-R cells treated with 1 μM selumetinib (+), were included as time 0 controls. Lysates were western blotted with the indicated antibodies. **c**, **d** Wild-type (p57 WT1) and knockout (p57 KO1, p57 KO2) C6244-R cells generated by CRISPR/Cas9 gene editing with guide RNA (gRNA#2) targeting *CDKN1C* (encoding p57^KIP2^) were treated with (+) selumetinib (Sel) or DMSO only (−) for 72 h. Untransfected (UT) COLO205 and C6244-R cells were included as controls. Lysates were western blotted with the indicated antibodies (**c**) or cell cycle profile determined (**d**). Results (**d**) are mean ± SD of three independent experiments. *P* < 0.001 (***) or *P* > 0.05 (ns) determined by one-way ANOVA with Tukey’s multiple comparisons test of G1 fractions. **e**, **f** Following 15 weeks culture in the presence (+) or absence (COLO205 and (−)) of 1 μM selumetinib, cells were washed and either treated with the indicated concentrations (10 nM to 10 μM) of selumetinib (Sel) for 24 h, and DNA synthesis assayed by [^3^H]thymidine incorporation (**e**), or incubated in selumetinib-free medium for 24 h and lysates western blotted with the indicated antibodies (**f**). Results (**e**) are mean ± SD of cell culture triplicates and normalized to control for each cell line. **g**, **h** HT29 and HT6244-R cells were treated with selumetinib-free medium (− Sel) for the indicated time periods and lysates western blotted with the indicated antibodies. Results (**a**–**c** and **f**–**h**) are representative of at least two experiments giving equivalent results

Compared with COLO205 cells, C6244-R cells exhibited increased expression of BRAF, increased p-MEK1/2, but parental levels of p-ERK1/2, restrained by the presence of selumetinib (Fig. 4a). Selumetinib withdrawal de-repressed this pool of p-MEK1/2, resulting in an immediate and sustained activation of ERK1/2 (Fig. 4a). This was followed by increased p-RSK (ERK1/2 substrate) and expression of FRA1 (ERK1/2 substrate and target gene) and cyclin D1 (CCND1, which links ERK1/2 signalling to cell cycle re-entry). Despite this, p-RB S795 and cyclin A (CCNA) levels decreased from 16 h onwards, consistent with the G1 arrest.

To assess the wider consequences of ERK1/2 hyperactivation, C6244-R cells were deprived of selumetinib for 4, 16 or 48 h or treated with fresh selumetinib-containing medium and RNA samples hybridized to Illumina Human HT-12 bead chips. Gene set enrichment analysis (GSEA)^23^ showed enrichment for the KEGG (Kyoto Encyclopedia of Genes and Genomes) MAPK (mitogen-activated protein kinase) signalling gene set (Supplementary Fig. 3a, b), a 16-gene MEK1/2 activation signature^24^ (Supplementary Fig. 3c) and enrichment for a senescence gene set^25^ (Supplementary Fig. 3d, e). The KEGG cell cycle gene set was negatively enriched upon selumetinib withdrawal (Supplementary Fig. 4a–c)); the few genes that were upregulated included *CCND1* and several cyclin-dependent kinase inhibitors (CDKIs). Indeed, the senescence GSEA revealed upregulation of *CDKN1A*/p21^CIP1^, *CDKN1C*/p57^KIP2^, *CDKN2B*/p15^INK4B^ and *CDKN2D*/p19^INK4D^ (Supplementary Fig. 3e). Excessive, persistent ERK1/2 signalling drives expression of p21^CIP1^ and p16^INK4A^, which promote cell cycle arrest and oncogene-induced senescence^26–32^. We observed a modest but delayed increase in p15^INK4B^, p16^INK4A^ and p19^INK4D^ expression, but a striking increase in p21^CIP1^ following selumetinib withdrawal (Fig. 4a). Although p21^CIP1^ can mediate ERK1/2-driven cell cycle arrest^26,27^, the G1 arrest following selumetinib withdrawal from C6244-R cells was unaffected by complete knockdown of p21^CIP1^ (Supplementary Fig. 4d, e). Indeed, p21^CIP1^ expression was transient and declining by 16 h, at which time the decline in p-RB S795 and G1 arrest became apparent. The levels of p21^CIP1^ and p16^INK4A^ following drug withdrawal were also extremely low in comparison with basal levels in HCT116 cells and HeLa cells (Supplementary Fig. 4f, g). We therefore examined p57^KIP2^, which exhibited the greatest fold increase in expression of any CDKI in the array data. p57^KIP2^ was strongly induced 16 h after selumetinib withdrawal, maintained throughout the G1 arrest and correlated well with the loss of p-RB S795 (Fig. 4a). Furthermore, the ERK1/2 inhibitor SCH772984 abolished p57^KIP2^ induction at all time points (Fig. 4b), defining p57^KIP2^ as a new target of anti-proliferative ERK1/2 signalling.

We used CRISPR/Cas9 gene editing to knockout p57^KIP2^ in C6244-R cells using two guide RNAs (gRNAs) and derived multiple targeted clones (p57 KO1, p57 KO2, etc) for each guide, as well as control clones (p57 WT1, WT2) that retained full induction of p57^KIP2^ upon selumetinib removal (Fig. 4c; Supplementary Figs. 5, 6). DNA sequencing confirmed *CDKN1C* mutations in the C6244-R p57^KIP2^ KO cells and the absence of mutations in WT cells. Loss of p57^KIP2^ in C6244-R clones prevented the loss of p-RB S795 and the G1 arrest observed following selumetinib withdrawal (Fig. 4c, d; Supplementary Fig. 6a, b). When subjected to drug withdrawal for 15 weeks, untransfected C6244-R and C6244-R p57^KIP2^ WT cells reverted to drug sensitivity and BRAF expression and p-ERK1/2 levels re-set to parental levels (Fig. 4e, f; Supplementary Figs. 5a, 5b, 6c, 6d)). In contrast, all four p57^KIP2^ KO CRISPR clones exhibited a profound delay in reversion; indeed, two clones failed to revert to a drug-sensitive phenotype following 15 weeks drug withdrawal; the remaining two clones exhibited different degrees of partial resensitization (Fig. 4e, f; Supplementary Figs. 5a, 6c). Furthermore, the p57^KIP2^ KO clones retained the upregulation of BRAF and consequent hyperactivation of ERK1/2 (Fig. 4f; Supplementary Figs. 5b, 6d).

HT6244-R cells deprived of selumetinib also reactivated ERK1/2 signalling and expressed CCND1 and p21^CIP1^ (Fig. 4g) but failed to increase p57^KIP2^ and only underwent a transient G1 arrest at 16 h (Fig. 3b) before progressing to ERK1/2-driven apoptosis (Fig. 3c–e). Expression of the pro-apoptotic protein BIM decreased following selumetinib withdrawal (Fig. 4h) consistent with it being repressed by ERK1/2^33–35^. However, we observed increased expression of another pro-apoptotic BH3-only protein, NOXA, and cleavage of BID to the activated form, tBID, following selumetinib withdrawal; this preceded PARP cleavage (Fig. 4h). In addition, LC3 was processed when HT6244-R cells were deprived of selumetinib, consistent with an increase in autophagy.

In summary, C6244-R and HT6244-R cells with amplified BRAF^V600E^ underwent ERK1/2-dependent cell cycle arrest/senescence (C6244-R) or death (HT6244-R) upon selumetinib withdrawal. In C6244-R cells, p57^KIP2^ was required for the G1 cell cycle arrest, loss of BRAF expression and determined the rate of reversal of drug resistance. Thus, ERK1/2-induced cell cycle arrest/senescence or cell death selects against those cells that retain amplification of BRAF^V600E^ upon withdrawal of selumetinib, favouring cells that do not exhibit BRAF^V600E^ amplification, which repopulate the culture as selumetinib-sensitive cells.

### A narrow window of ERK1/2 activity sustains MEKi-resistant cells with BRAF^V600E^ amplification

**Fig. 5:**
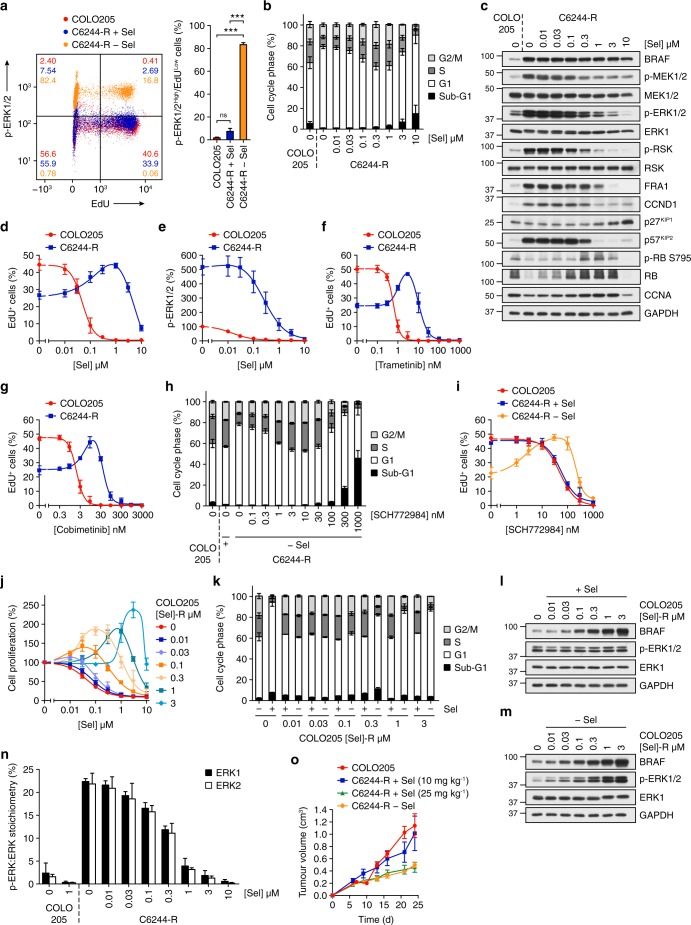
BRAF^V600E^-mutant COLO205 cells evolve resistance to MEKi by precisely reinstating parental ERK1/2 phosphorylation. **a** COLO205 and C6244-R cells were treated with 1 μM selumetinib (C6244-R + Sel) or DMSO only (COLO205, C6244-R − Sel) for 72 h, and phospho-ERK1/2 and EdU incorporation determined by flow cytometry. Representative plots (left) are shown with pooled quantifications (right). *P* < 0.001 (***) or *P* > 0.05 (ns) determined by one-way ANOVA with Tukey’s multiple comparisons test. **b**, **c** COLO205 and C6244-R cells were treated as indicated with selumetinib (Sel) for 72 h and cell cycle distribution determined (**b**) or lysates western blotted with the indicated antibodies (**c**). **d**–**g** COLO205 and C6244-R cells were treated as indicated with selumetinib (Sel) (**d**, **e**), trametinib (**f**) or cobimetinib (**g**) for 72 h. Percent EdU-positive (EdU^+^) cells (**d**, **f**, **g**) or phospho-ERK1/2 levels (**e**) were determined by high-content image analysis. Results are normalized to COLO205 control. **h**, **i** COLO205 and C6244-R cells were treated with SCH772984 (SCH) in the presence ((+), or + Sel) or absence of selumetinib (COLO205, − Sel) for 72 h. Cell cycle distribution was determined by flow cytometry (**h**) or EdU incorporation assessed by high-content image analysis (**i**). Results (**i**) are normalized to COLO205 control. **j** COLO205 cells with acquired resistance to the indicated concentrations of selumetinib (Sel) were treated as indicated with selumetinib for 24 h, and DNA synthesis assayed by [^3^H]thymidine incorporation. Results are normalized to control for each cell line. **k**–**m** COLO205 cells with acquired resistance to the indicated concentrations of selumetinib (COLO205 [Sel]-R) were treated with the respective concentration of selumetinib to which they had acquired resistance ((+) or + Sel) or with DMSO only ((−), or − Sel) for 72 (**k**) or 24 (**l**, **m**) hours. Cell cycle distribution was determined (**k**) or lysates western blotted with the indicated antibodies (**l**, **m**). **n** COLO205 and C6244-R cells were treated with the indicated concentrations of selumetinib (Sel) for 72 h. Lysates were then subjected to SDS-PAGE and gel bands containing ERK1 and ERK2 removed for absolute quantification of dual-phosphorylated and total ERK1 and ERK2 peptides by mass spectrometry. **o** Mice were dosed twice daily with vehicle only (C6244-R − Sel), or 10 or 25 mg kg^−1^ selumetinib, as indicated. On dosing day 2, 9–10 mice per group were injected with C6244-R cells and an additional group (*n* = 10) with parental COLO205 cells. Mice continued to be dosed twice daily and tumour growth recorded twice weekly. Results are mean ± SEM. Results (**c**, **l**, **m**) are representative of at least two experiments giving equivalent results, and results (**a**, **b**, **d**–**k**, **n**) are mean ± SD of at least three independent experiments

Our results suggested that BRAF^V600E^-amplified cells required selumetinib to maintain ERK1/2 signalling at an optimal level for proliferation. Drug withdrawal from C6244-R cells strikingly reduced their 5-ethynyl-2-deoxyuridine (EdU) staining (Supplementary Fig. 7a), consistent with cell cycle arrest. In all, 36% of C6244-R cells in selumetinib were EdU^High^ of which the vast majority were p-ERK1/2^Low^ (Fig. 5a), whereas drug withdrawal caused a profound shift of cells to p-ERK1/2^High^/EdU^Low^ (82%). Thus, those cells with the highest p-ERK1/2 underwent cell cycle arrest (Fig. 5a), G1 arrest (Fig. 5b) and expressed p57^KIP2^ (Fig. 5c). Cell cycle arrest upon selumetinib withdrawal was rescued by progressively higher concentrations of selumetinib up to 1 µM (Fig. 5d), the concentration at which C6244-R cells were selected and maintained. At this concentration, C6244-R cells exhibited equivalent levels of p-ERK1/2, ERK1/2 pathway output (p-RSK, FRA1), p57^KIP2^, p-RB S795 and CCNA as parental COLO205 (Fig. 5c–e) consistent with their normal cell cycle profile (Fig. 5b). The cell cycle arrest observed upon withdrawal of selumetinib was also rescued by low concentrations of two other MEKi, trametinib or cobimetinib (Fig. 5f, g). Indeed, 3 nM trametinib or 10 nM cobimetinib set ERK1/2 phosphorylation in C6244-R precisely to parental levels (Supplementary Fig. 7b, c) and allowed optimal proliferation (Fig. 5f, g). HT6244-R cells also exhibited ~ 100-fold resistance to trametinib and cobimetinib and proliferated optimally when ERK1/2 phosphorylation was clamped to parental HT29 levels by MEKi (Supplementary Fig. 7d–i)). C6244-R cell cycle arrest, p57^KIP2^ expression and loss of p-RB S795 and CCNA upon withdrawal of selumetinib were also rescued by low concentrations of the ERKi SCH772984, whereas higher concentrations caused cell cycle arrest (Fig. 5h; Supplementary Fig. 7j), and a bell-shaped concentration-response curve for cell proliferation (Fig. 5i) that correlated with inhibition of RSK phosphorylation (Supplementary Fig. 7k).

We next generated COLO205 cells that were resistant to a range of concentrations of selumetinib (Supplementary Fig. 7l, m)). Each cell line proliferated optimally in the concentration of selumetinib to which it had evolved resistance (Fig. 5j) and exhibited a normal, parental cell cycle profile and parental level of p-ERK1/2, enabled by a progressive increase in BRAF expression (Fig. 5k, l). However, upon drug withdrawal, each cell line hyperactivated ERK1/2 in proportion to the degree of BRAF expression (Fig. 5m) and those with the strongest ERK1/2 activation underwent G1 cell cycle arrest (Fig. 5k). Thus, COLO205 cells adapt to selumetinib by amplifying BRAF^V600E^ but this requires the presence of drug to maintain a narrow window of p-ERK1/2 that sustains proliferation while avoiding quiescence (hypoactivation) or arrest/senescence (hyperactivation).

To define this optimal level of ERK1/2 activation, we used mass spectrometry to provide absolute quantification of total and dual pT-E-pY phosphorylated ERK1 and ERK2. Remarkably, just 2–3% of the ERK1/2 was active in proliferating parental COLO205 cells and in resistant C6244-R cells maintained in 1 µM selumetinib (Fig. 5n). Lowering the selumetinib concentration caused a concentration-dependent increase in the active p-ERK1/2 pool (Fig. 5n), which correlated with the cell cycle arrest (Fig. 5b). For example, the G1 cell cycle arrest was maximal at 10 nM selumetinib, at which concentration 20–22% of the total cellular ERK1/2 was active.

We also examined selumetinib addiction in vivo by growing C6244-R tumour xenografts. Mice were dosed for 24 h with vehicle or 10 mg kg^−1^ selumetinib and then implanted with C6244-R cells; treatment with vehicle or selumetinib was maintained throughout the experiment. C6244-R tumours in mice dosed with 10 mg kg^−1^ selumetinib and parental COLO205 tumours grew at a similar rate, whereas C6244-R tumours in vehicle-dosed mice grew more slowly (Fig. 5o). In a third arm, mice received a higher dose of selumetinib (25 mg kg^−1^) before and after C6244-R implantation; this also inhibited tumour growth. Thus, withdrawing drug to activate ERK1/2 or increasing the selumetinib concentration to more strongly inhibit ERK1/2 impaired tumour growth, consistent with a narrow window of ERK1/2 activity maintaining C6244-R tumour growth in vivo (Fig. 5o). The effect of drug withdrawal on tumour growth was more apparent the longer the duration of the experiment. For example, C6244-R tumours regressed from 30 days onwards in mice treated with vehicle (Supplementary Fig. 7n). In this experiment, the tumours in selumetinib-treated mice reached a plateau in size; their failure to grow further may reflect the greater variability in in vivo drug exposure so that the tumour drug concentration may periodically have over- or under-shot the sweet spot for maintaining C6244-R cell proliferation. Thus, our results show that C6244-R cells are addicted to selumetinib to maintain a low level of ERK1/2 signalling that is optimal for proliferation in vitro and tumour growth in vivo.

### MEKi resistance driven by KRAS^G13D^ amplification is not reversible

**Fig. 6:**
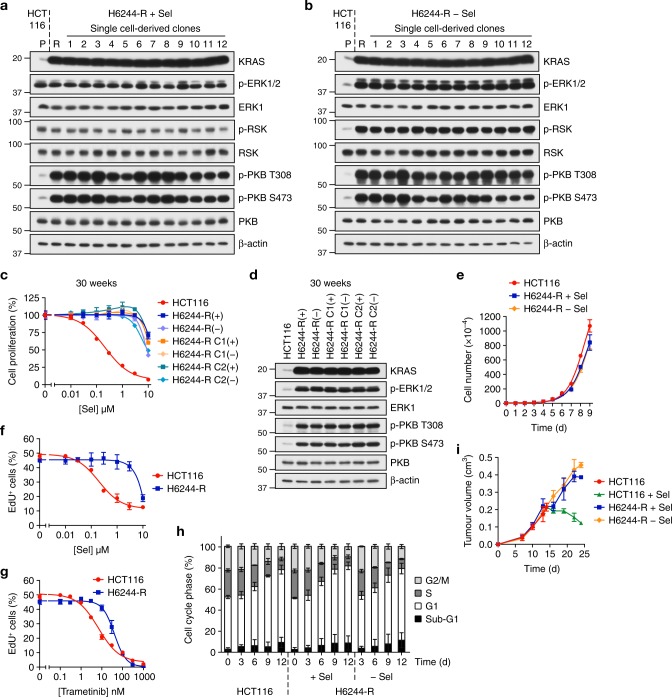
Acquired MEKi resistance driven by KRAS^G13D^ amplification is not reversible. **a**, **b** Non-clonal HCT116 cells with acquired resistance to selumetinib (H6244-R cells, R) and 12 single-cell clone derivatives (1–12) were treated with 2 μM selumetinib (Sel) (**a**) or selumetinib-free medium (**b**) for 24 h. Parental HCT116 cells (P) were treated in parallel with selumetinib-free medium. Lysates were western blotted with the indicated antibodies. **c**, **d** Following 30 weeks culture in the presence (+) or absence (HCT116, (−)) of 2 μM selumetinib, cells were treated as indicated with selumetinib (Sel) for 24 h, and DNA synthesis assayed by [^3^H]thymidine incorporation (**c**), or incubated in selumetinib-free medium for 24 h and lysates western blotted with the indicated antibodies (**d**). H6244-R C1 and C2 are single-cell clone derivative cell lines of H6244-R. Results (**c**) are mean ± SD of cell culture triplicates and normalized to control for each cell line. **e** HCT116 and H6244-R cells were treated with either 2 μM selumetinib (H6244-R + Sel) or DMSO only (HCT116, H6244-R − Sel) and cell numbers counted over 9 days. Results are mean ± SD of cell culture triplicates, representative of three independent experiments. **f**, **g** HCT116 and H6244-R cells were treated as indicated with selumetinib (Sel) (**f**) or trametinib (**g**) for 72 h. Percent EdU-positive (EdU^+^) cells was determined by high-content image analysis. Results are normalized to HCT116 control. **h** HCT116 and H6244-R cells were treated with either 2 μM selumetinib (H6244-R + Sel) or DMSO only (HCT116, H6244-R − Sel) for the indicated times. Cell cycle distribution was determined by flow cytometry. **i** Mice were dosed orally with 50 mg kg^−1^ selumetinib and the following day injected subcutaneously with H6244-R cells. Nine days after transplantation mice were randomized in to two groups: the next day selumetinib dosing either continued (H6244-R + Sel; *n* = 16) or was withdrawn (H6244-R − Sel; *n* = 16). In parallel, three mice were injected with HCT116 cells, allowed to grow for 14 days (HCT116) before receiving 50 mg kg^−1^ selumetinib (HCT116 + Sel). Tumour sizes were recorded twice a week. Results are mean ± SEM. **f**, **g**, **h** Results are mean ± SD of three independent experiments

We also examined selumetinib-resistant HCT116 (H6244-R) and LoVo (L6244-R) cells (both expressing KRAS^G13D^, Supplementary Table 1). HCT116 cells adapt to selumetinib by amplifying KRAS^G13D^ to re-instate ERK1/2 signalling^20^. RNA-seq confirmed selective amplification of the KRAS^G13D^ mutant allele (Supplementary Fig. 8a) and revealed a reduction in the wild-type KRAS allele; this may relate to a tumour-suppressor function for wild-type RAS^36–39^. All single-cell clones derived from H6244-R cells exhibited elevated KRAS expression, parental levels of p-ERK1/2 in the presence of drug (Fig. 6a) and strong ERK1/2 activation upon selumetinib withdrawal (Fig. 6b); H6244-R cells also exhibited a striking hyperphosphorylation of PKB. Remarkably, even after 30 weeks selumetinib withdrawal H6244-R cells were fully resistant to selumetinib (Fig. 6c; Supplementary Fig. 9a), whereas reversion was apparent after 2.5 weeks of drug withdrawal in BRAF^V600E^ amplified C6244-R cells (Supplementary Fig. 1a). H6244-R did not exhibit loss of KRAS expression or reversal of hyperactivated signalling to ERK1/2 or PKB upon drug withdrawal (Fig. 6d; Supplementary Fig. 9b). Drug withdrawal did not affect the rate of H6244-R proliferation over 9 days (Fig. 6e); neither was there a change in EdU incorporation, cell cycle profile or cell death upon drug withdrawal despite an equivalent increase in ERK1/2 phosphorylation (~ 500% over parental HCT116) as the BRAF^V600E^-amplified cells (Fig. 6f–h; Supplementary Fig. 8b–f). H6244-R cells were cross-resistant to trametinib and cobimetinib, but unlike BRAF^V600E^ amplified cells, low doses of these MEKi did not augment proliferation (Fig. 6g and Supplementary Fig. 8b. Although HCT116 tumour xenograft growth was inhibited when mice were treated with selumetinib at day 14, H6244-R tumours grew at a similar rate regardless of whether the mice received selumetinib or vehicle (Fig. 6i). Thus, H6244-R cells were not addicted to the presence of selumetinib for proliferation in vitro or tumour growth in vivo.

**Fig. 7:**
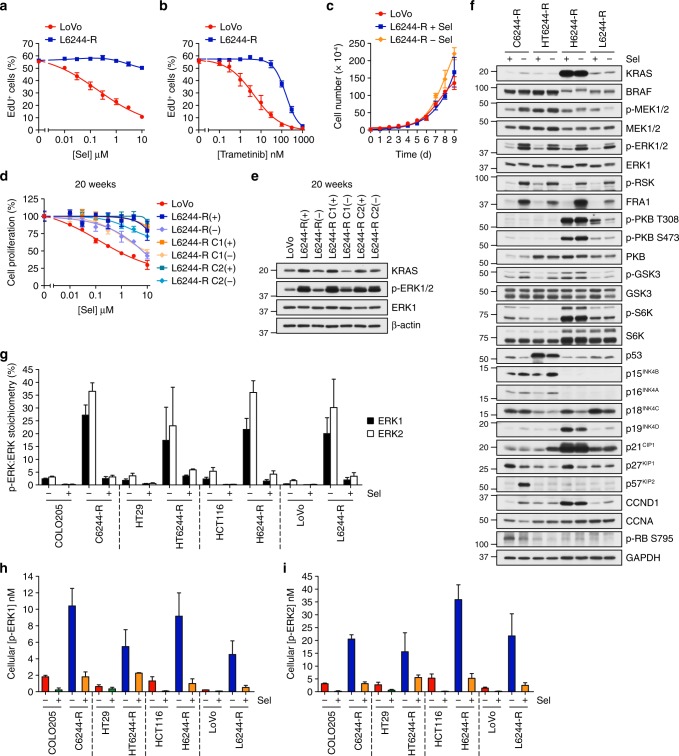
MEKi withdrawal from BRAF^V600E^- or KRAS^G13D^-amplified cells causes equivalent ERK1/2 hyperactivation. **a**, **b** LoVo and L6244-R cells were treated with the indicated concentrations of selumetinib (Sel) (**a**) or trametinib (**b**) for 72 h. Percent EdU-positive (EdU^+^) cells was determined by high-content image analysis. Results are normalized to LoVo control. **c** LoVo and L6244-R cells plated in their normal growth medium were treated with either 4 μM selumetinib (L6244-R + Sel) or DMSO only (LoVo, L6244-R − Sel) and cell numbers counted over 9 days. Results are mean ± SD of cell culture triplicates, representative of two experiments. **d**, **e** Following 20 weeks culture in the presence (+) or absence (LoVo, (−)) of 4 μM selumetinib, cells were treated with the indicated concentrations (10 nM to 10 μM) of selumetinib (Sel) for 24 h, and DNA synthesis assayed by [^3^H]thymidine incorporation (**d**), or incubated in selumetinib-free medium for 24 h and lysates western blotted with the indicated antibodies (**e**). Results (**d**) are mean ± SD of cell culture triplicates and normalized to control for each cell line. **f** C6244-R, HT644-R, H6244-R and L6244-R cells were treated with 1, 1, 2 and 4 µM selumetinib (Sel, (+)), respectively, or with DMSO only (−), for 72 h. Lysates were western blotted with the indicated antibodies. Results are representative of at least two experiments giving equivalent results. **g**–**i** COLO205/C6244-R, HT29/HT6244-R, HCT116/H6244-R and LoVo/L6244-R cells were treated with 1, 1, 2, 4 µM selumetinib (Sel), respectively, or with DMSO only (−) for 72 h. Lysates were then subjected to SDS-PAGE and gel bands containing ERK1 and ERK2 removed for absolute quantification of dual-phosphorylated and total ERK1 and ERK2 peptides by mass spectrometry. Results are p-ERK/ERK stoichiometry (%) (**g**) or cellular concentrations of p-ERK calculated using cell volume measurements (**h**, **i**). Results (**a**, **b**, **g**–**i)** are mean ± SD of three independent experiments

The increase in KRAS expression in selumetinib-resistant LoVo cells (L6244-R cells)^20^ was not due to a copy number change in *KRAS* but RNA-seq confirmed a moderate proportional increase of both the wild-type and KRAS^G13D^ alleles (Supplementary Fig. 10a, b). This increase in total KRAS was variable, with 5 of 12 single-cell clones exhibiting a more modest increase in KRAS (Supplementary Fig. 10c) and yet all 12 clones exhibited normal, parental p-ERK1/2 in the presence of selumetinib and strong ERK1/2 activation when drug was withdrawn (Supplementary Fig. 10d). RNA-seq revealed the presence of additional mutations that might contribute to ERK1/2 pathway activation in L6244-R cells (Supplementary Fig. 10e) including a mutation in *MAP2K1* encoding a MEK1^G128D^ that most likely disrupts MEKi binding^15^ and a mutation in *GNAI1* encoding GNAI1^H322N^ a Giα1 subunit of heterotrimeric GTPases that may contribute to activation of ERK1/2 and other signalling pathways. The significance of mutations in *NF1* (a −1384 frameshift, which could increase RAS-GTP by disrupting NF1 RAS-GAP activity) and *BRAF* (S429Y, a potentially oncogenic mutant by Functional Analysis through Hidden Markov Models) is unclear due to the low frequency of supporting reads (Supplementary Fig. 10e). So MEKi resistance in most L6244-R clones reflects an increase in both KRAS^WT^/KRAS^G13D^ expression in combination with other mutations that may drive ERK1/2 signalling. L6244-R cells exhibited striking resistance to selumetinib, trametinib and cobimetinib (Fig. 7a, b; Supplementary Fig. 10f)) and a ~ 4-fold increase in ERK1/2 phosphorylation upon selumetinib withdrawal (Supplementary Fig. 10g–i). As with H6244-R cells, we observed no proliferation deficit upon selumetinib withdrawal from L6244-R cells (Fig. 7a–c; Supplementary Fig. 10f, j, k). In resistance reversibility experiments some clones exhibited a partial reversion while others were refractory to drug withdrawal (Fig. 7d, e; Supplementary Fig. 11). The lack of any proliferation deficit when H6244-R or L6244-R cells were deprived of selumetinib correlated with the lack of increase in expression of any CDKIs in these cells (Fig. 7f). This was not due to a lower level of ERK1/2 hyperactivation in these cells upon drug withdrawal (Fig. 7f–i). Absolute quantification of total and dual pT-E-pY phosphorylated ERK1 and ERK2 revealed a p-ERK:ERK stoichiometry of ~ 20–35% in H6244-R and L6244-R in the absence of drug, which was equivalent to BRAF^V600E^-amplified C6244-R and HT6244-R cells (Fig. 7g). In all cases, the stoichiometry in parental cells, ~ 1–5%, and resistant cells maintained in selumetinib, was very similar. To better compare active ERK levels across the cell lines and account for any differences in total ERK expression, we used these results to calculate the global cellular p-ERK concentration following cell volume measurement. This analysis confirmed that in all cases p-ERK1 and p-ERK2 concentrations were very similar in parental vs. resistant cells in drug (p-ERK1, ~ 0.5–2 nM; p-ERK2, ~ 2–5 nM), and that the extent of p-ERK1/2 hyperactivation (p-ERK1, ~ 5–10 nM; p-ERK2, ~ 15–35 nM) did not correlate with or account for the different phenotypes observed between BRAF and KRAS amplified/upregulated cells upon selumetinib withdrawal.

### MEKi withdrawal from resistant cells with KRAS^Mut^ drives an ERK1/2- and ZEB1-dependent EMT and chemoresistance

**Fig. 8:**
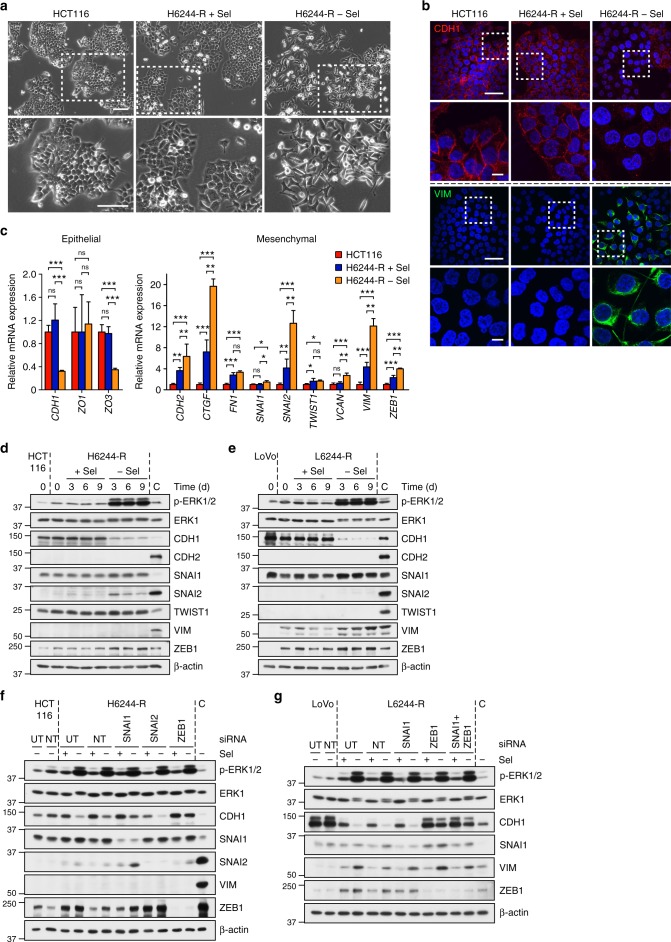
MEKi withdrawal from cells with KRAS^G13D^ amplification/upregulation induces a ZEB1-dependent EMT. **a** HCT116 and H6244-R cells were treated with 2 μM selumetinib (H6244-R + Sel) or DMSO only (HCT116, H6244-R − Sel) and imaged by brightfield phase contrast microscopy after 9 days. Scale bars indicate 100 µm. **b** HCT116 and H6244-R cells were treated with 2 μM selumetinib (H6244-R + Sel) or DMSO only (HCT116, H6244-R − Sel) for 9 days and stained for CDH1 (red) or VIM (green) and nuclei (blue). Scale bars indicate 50 µm (upper panels) and 10 µm (lower panels). **c** HCT116 and H6244-R cells were treated with 2 μM selumetinib (H6244-R + Sel) or DMSO only (HCT116, H6244-R − Sel) for 9 days and relative expression of the indicated mRNAs (normalized to *B2M*) determined by RT-qPCR. Results are mean ± SD of at least three independent experiments. *P* < 0.001 (***), *P* < 0.01 (**), *P* < 0.05 (*), *P* > 0.05 (ns) as determined by one-way ANOVA with Tukey’s multiple comparisons test. **d** HCT116 and H6244-R cells were treated with 2 μM selumetinib ( + Sel) or DMSO only (HCT116, − Sel) for the indicated times. **e** LoVo and L6244-R cells were treated with 4 μM selumetinib ( + Sel) or DMSO only (LoVo, − Sel) for the indicated times. **f** HCT116 and H6244-R cells were either left untransfected (UT), transfected with non-targeting (NT) siRNA or transfected with SNAI1-, SNAI2-, or ZEB1-specific siRNA as indicated. Twenty-four hours later, cells were treated with 2 μM selumetinib (+) or DMSO only (−) for 48 h. **g** LoVo and L6244-R cells were either left untransfected (UT), transfected with non-targeting (NT) siRNA or transfected with SNAI1- and/or ZEB1-specific siRNAs as indicated. Twenty-four hours later cells were treated with 4 μM selumetinib (+) or DMSO only (−) for 48 h. **d**–**g** Lysates were western blotted with the indicated antibodies and results are representative of at least two experiments giving equivalent results. A549 cells were used for positive control (C), except for SNAI2 (SW620 cells)

H6244-R cells exhibited striking changes in cell morphology upon selumetinib withdrawal. HCT116 cells, and H6244-R cells maintained in selumetinib, exhibited an epithelial morphology, with pronounced cell–cell contacts that stained well for E-cadherin (CDH1; Fig. 8a, b). H6244-R cells deprived of selumetinib for 9 days exhibited loss of cell–cell contacts, elongated protrusions, grew over each other and were phase bright (Fig. 8a). H6244-R cells lost CDH1 following drug withdrawal but acquired expression of the mesenchymal marker vimentin (VIM), which was absent in HCT116 cells or H6244-R cells maintained in selumetinib (Fig. 8b). These changes are typical of an EMT; indeed RT-qPCR (reverse transcription quantitative polymerase chain reaction) confirmed the loss of the epithelial markers *CDH1* and *ZO3*, and increased expression of the mesenchymal markers *CDH2*, *CTGF*, *SNAI2*, *VIM* and *ZEB1* in H6244-R cells deprived of selumetinib (Fig. 8c). L6244-R cells also underwent EMT following drug withdrawal (Supplementary Fig. 12a–c). Several EMT-promoting transcription factors were upregulated following selumetinib withdrawal from H6244-R, most strikingly SNAI1, SNAI2 and ZEB1; these effects were apparent at 24 h (Supplementary Fig. 13a), and were well established following 3 days of drug withdrawal, and correlated with repression of CDH1 (Fig. 8d). Similar upregulation of VIM and ZEB1 and suppression of CDH1, was observed in L6244-R cells upon selumetinib withdrawal (Fig. 8e and Supplementary Fig. 13b).

We employed RNA interference to assess the role of SNAI1, SNAI2 and ZEB1. Although non-targeting siRNA or siRNA to SNAI1 or SNAI2 knockdown had no effect, ZEB1 knockdown almost completely prevented the repression of CDH1 observed upon selumetinib withdrawal in H6244-R cells (Fig. 8f). Very similar results were observed in L6244-R cells (Fig. 8g). Thus, rather than eliciting cell cycle arrest or cell death, withdrawal of selumetinib from H6244-R or L6244-R cells (with increased KRAS^G13D^) resulted in a ZEB1-dependent EMT.

**Fig. 9:**
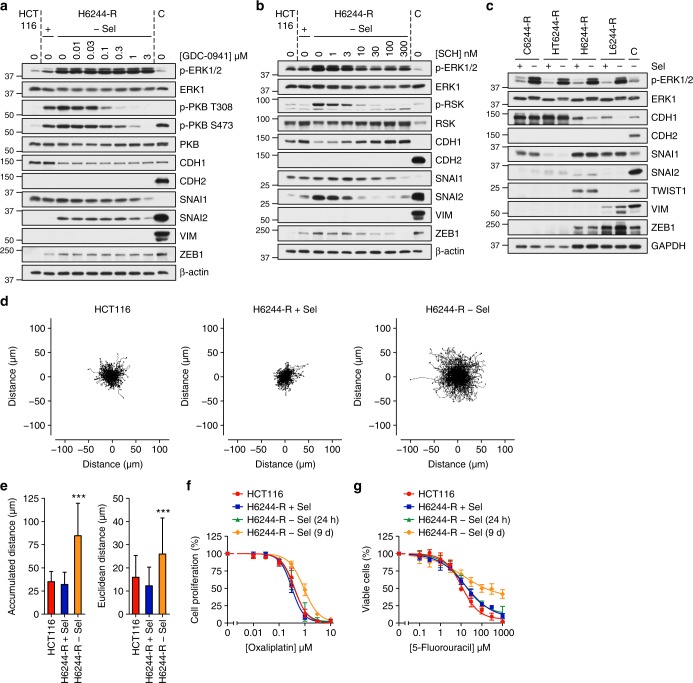
EMT following MEKi withdrawal is ERK1/2-dependent and associated with enhanced cell motility and chemoresistance. **a**, **b** H6244-R cells were treated with the indicated concentrations of GDC-0941 (**a**) or SCH772984 (SCH) (**b**) in the absence of selumetinib (− Sel) for 72 h. HCT116, and H6244-R cells treated with 2 μM selumetinib (+), were included as controls. **c** C6244-R, HT644-R, H6244-R and L6244-R cells were treated with 1, 1, 2 and 4 µM selumetinib (Sel, (+)), respectively, or with DMSO only (−) for 72 h. **a**–**c** Lysates were western blotted with the indicated antibodies. Results are representative of at least two experiments giving equivalent results and A549 cells were used for positive control (C), except for SNAI2 (SW620 cells). **d**, **e** HCT116 and H6244-R cells were treated with 2 μM selumetinib (H6244-R + Sel) or DMSO only (HCT116, H6244-R − Sel) for 9 days. Cell movements were then tracked every 10 min for 16 h. Spider diagrams of individual cell paths (**d**) are shown with averaged results (**e**) of mean accumulated and Euclidean distance ± SD (*n* > 300 cells for each cell line). *P* < 0.001 (***) determined by one-way ANOVA with Tukey’s multiple comparisons test. **f**, **g** HCT116 and H6244-R cells were treated with 2 μM selumetinib (H6244-R + Sel) or DMSO only (HCT116, H6244-R − Sel (9 d)) for 9 days. The following day, cells were treated as indicated with oxaliplatin for 24 h (**f**) or 5-fluorouracil for 72 h (**g**), maintaining prior presence or absence of selumetinib. As a control, H6244-R cells maintained in selumetinib throughout, were switched to selumetinib-free medium for the last 24 h of oxaliplatin or 5-fluorouracil treatment (H6244-R − Sel (24 h)). Cell proliferation was assayed by [^3^H]thymidine incorporation (**f**) or cell viability assayed using Sytox Green (**g**). Results are mean ± SD of three independent experiments and normalized to control for each cell line

In addition to ERK1/2, PI3K (phosphatidylinositol-3-kinase) signalling can also promote EMT^40^ and both pathways are activated downstream of amplified KRAS^G13D^ (Fig. 6a, 9a)^20^. Although the PI3K inhibitor GDC-0941 inhibited PI3K (loss of p-PKB) it had no effect on ZEB1 or CDH1 expression (Fig. 9a), whereas the ERK1/2 inhibitor SCH772984 prevented the increase in ZEB1 and SNAI2 and the loss of CDH1 following selumetinib withdrawal in H6244-R cells (Fig. 9b); similar results were obtained in L6244-R cells (Supplementary Fig. 13c). Thus, the EMT observed upon withdrawal of selumetinib was ERK1/2-dependent but PI3K independent. Despite this, only resistant cells with elevated KRAS^G13D^, not BRAF^V600E^, underwent EMT upon drug withdrawal as judged by loss of CDH1 levels (Fig. 9c). Indeed, both KRAS^G13D^ cell lines expressed markedly higher levels of ZEB1 and SNAI1 and lower levels of CDH1 compared with the BRAF^V600E^ cell lines even in the presence of selumetinib. Thus, likely due to increased KRAS^G13D^ expression, H6244-R and L6244-R cells were further along an EMT continuum than the BRAF^V600E^ amplified cell lines, such that selumetinib withdrawal and ERK1/2 hyperactivation was able to drive a pronounced EMT.

EMT confers enhanced cell motility and we observed that H6244-R cells exhibited faster monolayer wound closure when deprived of selumetinib (Supplementary Fig. 13d, e). This accelerated wound closure most likely reflects the enhanced motility of H6244-R, which exhibited a 2- to 3-fold increase in the mean accumulated distance and a 1.5- to 2-fold increase in the Euclidean (as the crow flies) distance upon selumetinib withdrawal in undirected motility assays (Fig. 9d, e); similar results were observed in L6244-R cells (Supplementary Fig. 13f, g). Prompted by the increased motility of H6244-R cells, we investigated whether this was manifest as increased metastasis or invasion in vivo. Initial attempts at orthotopic colonic xenografts were hindered by technical difficulties and so subcutaneous xenografts were undertaken. We observed no difference in the invasive potential of H6244-R cells growing in mice dosed with or without selumetinib; ~ 40% of tumours invaded adjacent fat or muscle tissue regardless of drug treatment (Supplementary Table 2). No liver or lung metastases were observed in any case.

Recent studies have argued that EMT can confer resistance to chemotherapy agents^41,42^. We examined this using 5-fluorouracil (5-FU) and oxaliplatin, both agents that are frequently used in the treatment of colorectal cancer. Long-term withdrawal of selumetinib from H6244-R cells for 9 days, which causes a profound EMT, reduced sensitivity to oxalipatin and 5-FU (Fig. 9f, g). A similar but more modest shift in response to 5-FU, but not oxaliplatin, was also observed in L6244-R cells upon chronic selumetinib withdrawal (Supplementary Fig. 13h–j). This resistance was not simply due to the known anti-apoptotic effects of ERK1/2 signalling as 24 h of selumetinib withdrawal failed to confer resistance and yet promoted the same magnitude of ERK1/2 activation as 9 days selumetinib withdrawal (Fig. 8d; Supplementary Fig. 13a). Thus, chemoresistance required long-term selumetinib withdrawal that drove EMT re-programming.

## Discussion

We show here that acquired resistance to MEKi driven by BRAF^V600E^ amplification is reversible upon drug withdrawal because BRAF^V600E^ amplification confers a selective disadvantage in the absence of MEKi. The magnitude of ERK1/2 reactivation upon selumetinib withdrawal is not tolerated and drives cell cycle arrest and senescence, or cell death with features of apoptosis and autophagy, selecting against those cells with BRAF^V600E^ amplification. This is consistent with a recent study in PDX models where elevated BRAF^V600E^ only conferred a growth advantage when an ERKi was present; ERKi withdrawal drove a reduction in *BRAF* copy number suggesting that the magnitude of ERK1/2 signalling was a critical fitness threshold^43^. Indeed, our co-culture experiments confirmed that BRAF^V600E^ amplification conferred a fitness deficit in the absence of a restraining MEKi (Fig. 2d).

In C6244-R cells, strong ERK1/2 activation drove expression of p57^KIP2^, which promoted cell cycle arrest, was required for the loss of BRAF^V600E^ amplification and determined the rate of reversal of MEKi resistance. Thus, p57^KIP2^ links ERK1/2 hyperactivation to reversal of MEKi resistance, representing a novel mechanism by which excessive ERK1/2 signalling halts cell proliferation and may promote senescence. This regulation is likely transcriptional, analogous to *CDKN1A*/p21^CIP1^, although p57^KIP2^ induction appears to be p53 independent since it was induced in C6244-R cells (p53^Mut^) but not in HCT116 or LoVo (p53^WT^) and selumetinib withdrawal did not increase p53 abundance (Fig. 7f). Rather, the *CDKN1C* gene contains several classic ERK1/2-responsive elements^44,45^. HT6244-R cells also lost BRAF^V600E^ amplification upon MEKi withdrawal; these cells increased NOXA and progressed to caspase-dependent cell death with features of autophagy. Interestingly, NOXA has previously been implicated in autophagic cell death arising from strong ERK1/2 activation^46,47^. Our results indicate that during selumetinib withdrawal those cells with BRAF^V600E^ amplification are lost because they cannot tolerate the level of ERK1/2 hyperactivation and undergo cell cycle arrest, senescence or death with the culture being repopulated by cells with no BRAF^V600E^ amplification and moderate ERK1/2 signalling. These results are consistent with those in BRAFi + MEKi- or MEKi-resistant melanoma, in which responses to ERK1/2 hyperactivation following drug withdrawal ranged from transient slow cycling to cell death^48^. That resistance was reversible from single-cell clone derivatives of C6244-R and HT6244-R cells, and that these original single cells must have harboured *BRAF* amplification in order to thrive, strongly suggests that loss of *BRAF* copy number can occur on an individual cell level rather than simply result from the outgrowth of rare dormant parent-like cells that repopulate the culture once selumetinib is withdrawn. This is supported by FISH in these revertant cells, which suggested that entire chromosomes (C6244-R) or whole *BRAF* amplicons (C6244-R and HT6244-R) were lost during the process of reversion (Fig. 1g, h).

Implicit in this model is the notion that ERK1/2 signalling operates within tightly defined parameters to drive tumour cell proliferation. Indeed, C6244-R cells with the highest level of p-ERK1/2 exhibited the lowest proliferation but cell cycle arrest was rescued by low concentrations of MEKi (selumetinib, trametinib, cobimetinib) or ERKi (SCH772984). Furthermore, COLO205 cells selected in increasing concentrations of selumetinib upregulated BRAF expresssion in a concentration-dependent manner to those levels required to sustain proliferative ERK1/2 signalling in the presence of selumetinib. This was also reflected in vivo; C6244-R cells grew as xenografts in mice dosed with 10 mg kg^−1^ selumetinib, whereas increasing the selumetinib dose to 25 mg kg^−1^ or withdrawing the drug inhibited tumour growth. Thus, ERK1/2 signalling operates within a strict sweet spot or fitness threshold^43^ to drive tumour cell proliferation with extremes of ERK1/2 signalling being associated with quiescence (low p-ERK1/2) or cell cycle arrest/senescence/death (high p-ERK1/2). Quantification of p-ERK1/2 by mass spectrometry revealed that parental COLO205 cells proliferated with just 2–3% of their ERK1/2 pool active; similar results were seen in HT29 cells, and KRAS^Mut^ HCT116 and LoVo cells (Fig. 7g–i). Remarkably, C6244-R cells in selumetinib exhibited near-identical stoichiometry of ERK1/2 activation despite the amplification of BRAF^V600E^, whereas selumetinib withdrawal to drive cell cycle arrest was associated with 20–30% of the ERK1/2 active. These studies also reveal a substantial spare capacity for ERK1/2 activation that is likely maintained through the action of the DUSP family of MAPK phosphatases. Indeed, DUSP5, a nuclear ERK1/2-specific phosphatase, limits BRAF^V600E^-induced ERK1/2 activation to prevent senescence^49^.

Our results provide a clear rationale for intermittent treatment (drug holidays), to delay or overcome emergent resistance; indeed, intermittent vemurafenib dosing in BRAF^V600E^-mutant melanoma forestalls resistance compared with continual dosing^50^. Critically, this only applied to MEKi resistance driven by BRAF^V600E^ amplification. Cells where MEKi resistance was driven by KRAS^G13D^ amplification/upregulation did not exhibit a fitness deficit upon drug withdrawal; MEKi resistance was maintained even after 30 weeks of drug withdrawal and growth of H6244-R xenografts was unaffected by the presence or absence of selumetinib. This was not due to a lower level of ERK1/2 activation; all resistant cell models, regardless of BRAF^V600E^ or KRAS^G13D^ amplification, exhibited similar levels of p-ERK1/2, whether basal or upon drug withdrawal. Thus, the response of MEKi-resistant cells to drug withdrawal or drug holiday may be determined by the nature of the amplified driving oncogene. This suggests that the anti-proliferative effects of excessive ERK1/2 signalling may be tempered or mitigated by other KRAS effector pathways^51^ or other metabolic, epigenetic, or genomic changes that are not mimicked by BRAF^V600E^ amplification. Notably, different KRAS^Mut^ tumour models respond very differently to MEKi treatment: acute myeloid leukaemia and colorectal cancer models evolved to amplify KRAS^Mut^, lost KRAS^WT^ and remained ERK1/2 dependent^38;^ pancreatic ductal adenocarcinoma genetically engineered mouse models (GEMMs) subjected to MEKi + ERKi treatment amplified KRAS^G12D^ to maintain ERK1/2 signalling, whereas non-small-cell lung cancer GEMM models did not alter KRAS^G12D^ frequency and downregulated ERK1/2 signalling^52^. Thus, in KRAS^Mut^ tumours the response to ERK1/2 pathway inhibition is complicated by the diversity of RAS effector pathways and codon-dependent differences in effector pathway use, whereas tissue-specific differences in mutant allele frequency will further complicate how tumours evolve to ERK1/2 pathway inhibition, including whether they remain ERK1/2 dependent.

ERK1/2 hyperactivation in the context of KRAS^G13D^ amplification/upregulation drove ZEB1-dependent EMT and enhanced cell motility. ERK1/2 can induce *ZEB1* mRNA via FRA1, and also promote interaction between ZEB1 and the CtBP co-repressor complex to repress *CDH1* transcription^53,54^. Although selumetinib withdrawal did not confer increased invasiveness in vivo, which may be a shortcoming of a subcutaneous xenograft model^55^, EMT was associated with resistance to chemotherapeutics. Thus, while drug holidays should be considered in dosing regimens in cases of BRAF^V600E^, our results argue against this in cases where ERK1/2 pathway inhibitor resistance is driven by KRAS amplification. This may be relevant to MEKi resistance in models with KRAS mutation because MEKi efficacy is likely to require the use of drug combinations that include conventional chemotherapy^2^. Finally, our results further underscore the wider challenges of treating RAS-driven tumours and the need for direct RAS-targeted therapies.

## Methods

### Reagents and resources

A full list of reagents and resources used in this study is provided in Supplementary Table 3. Further information and requests for resources and reagents should be directed to Simon J. Cook (simon.cook@babraham.ac.uk).

### Cell lines

A549 (human, male), COLO205 (human, male), HeLa (human, female), HT29 (human, female) and SW620 (human, male) were purchased from ATCC (distributor LGC Standards, UK). HCT116 (human, male) were provided by the laboratory of Professor Bert Vogelstein (The Johns Hopkins University, Baltimore, USA), and LoVo (human, male) from the laboratory of Professor Kevin Ryan (The Beatson Institute for Cancer Research, Glasgow, UK). Cells were grown in Dulbecco’s modified Eagle’s medium (DMEM) (A549, HCT116, HeLa, LoVo), Liebovitz’s L-15 (SW620), McCoy’s 5A (HT29) or RPMI-1640 (COLO205) media supplemented with 10% (v/v) foetal bovine serum, penicillin (100 U mL^−1^), streptomycin (100 mg mL^−1^) and 2 mM glutamine. Liebovitz’s L-15 medium was additionally supplemented with 0.75 mg mL^−1^ sodium bicarbonate. Cells were incubated in a humidified incubator at 37 °C and 5% (v/v) CO_2_. All cell lines were authenticated by short tandem repeat (STR) profiling and confirmed negative for mycoplasma. All reagents were from Gibco, Thermo Fisher Scientific (Paisley, UK). Selumetinib-resistant COLO205 (C6244-R), HCT116 (H6244-R), HT29 (HT6244-R) and LoVo (L6244-R) cells used in this study were generated previously^20^ by culturing cells in escalating concentrations of selumetinib (COLO205 and HCT116), or a chronic maximal concentration method (HT29 and LoVo), until cells grew in 10 × IC_50_ selumetinib at a stable rate similar to that of parental cells. C6244-R and HT6244-R cells were grown in the same media as their parent cell line with the addition of 1 µM selumetinib. H6244-R and L6244-R cells were grown in the same media as their parent cell line with the addition of 2 µM and 4 µM selumetinib, respectively.

### Mice

The care and use of all mice in this study was performed in accordance with UK Home Office regulations, UK Animals Scientific Procedures Act of 1986 and with AstraZeneca Global Bioethics policy. Experimental details are outlined in Home Office Project license 40/3483, which has gone through the AstraZeneca Ethical Review Process. Mice were maintained in a pathogen-free environment on a 12 h light: 12 h dark cycle with lights off at 19:30 and no twilight period. The ambient temperature was 21 ± 2 °C and the humidity was 55 ± 10%. Mice were housed at 3–5 mice per cage (365 × 207 × 140 mm, floor area 530 cm^2^) in individually ventilated caging (Tecniplast Sealsafe 1284L) receiving 60 air changes per hour. Mice were given water and food ad libitum. Female athymic mice (nu/nu:Alpk; AstraZeneca) were bred at AstraZeneca. Female SCID (NOD.CB17-Prkdcscid/J;) mice used in this study were purchased from The Jackson Laboratory (Maine, USA).

### Cell culture compound treatments

Cells were seeded in to dishes or plates in their normal growth medium (containing selumetinib for resistant cells) and allowed to settle for 24 h. For experiments with resistant cells, all treatment groups were washed with complete media only and then treated with fresh media containing the indicated compounds or media containing vehicle only. Vehicle concentrations (typically dimethyl sulfoxide, dimethylsulfoxide (DMSO)) were normalized so that they were equivalent for all treatments.

### Generation of selumetinib-resistant cells and drug withdrawal from resistant cell lines

The selumetinib-resistant colorectal cancer cells lines COLO205 (C6244-R), HCT116 (H6244-R), HT29 (HT6244-R) and LoVo (L6244-R) cells used in this study were generated previously by culturing cells in escalating concentrations of selumetinib (COLO205 and HCT116), or a chronic maximal concentration method (HT29 and LoVo), until cells grew in 10 × IC_50_ selumetinib at a stable rate similar to that of parental cells^20^. To generate single-cell clones of these cell lines, cells in media containing selumetinib at a density of 1 × 10^7^ cells mL^−1^ were filtered with a 30 µm filter (Sysmex, Milton Keynes, UK) to eliminate cell clumps and labelled with 4,6-diamidino-2-phenylindole (DAPI; Sigma-Aldrich, Dorset, UK) to allow exclusion of non-viable cells. DAPI-negative single cells were then sorted in to 96-well plates using a 100 µm nozzle on a BD FACSARIA III cell sorter (BD Biosciences, Oxford, UK). Single-cell clones were then expanded, maintaining the presence of selumetinib throughout, and media changed at least once a week.

To generate COLO205 cells with acquired resistance to a range of concentrations of selumetinib, cells 50% confluent in 175 cm^2^ tissue culture flasks were treated with the concentrations of selumetinib indicated in the Figure legends (0.01 µM–10 µM). Cells were then either split as required or media changed at least once a week until all cells grew at a stable rate that was similar to parental COLO205 cells. Cumulative doublings tallies were calculated by tracking splitting ratios and using the formula: number of doublings = log_2_(splitting dilution factor).

To examine the long-term effects of selumetinib withdrawal from selumetinib-resistant cells, 75 cm^2^ tissue culture flasks at 50% confluence were washed once with media only and then either treated with media containing selumetinib (+) or drug-free media (−). Cells were then either split as required or media changed at least once a week until cells grew at a stable rate that was similar to parental cells. Cumulative doublings tallies were calculated by tracking splitting ratios and using the formula: number of doublings = log_2_(splitting dilution factor).

### CRISPR-mediated gene editing

gRNAs to *CDKN1C* (encoding p57^KIP2^) were designed using Horizon Discovery gUIDEbook (Horizon Discovery, Cambridge, UK) and cloned into a pD1301-AD mammalian Cas9 (double-stranded nuclease *Steptococcus pyrogenes* Cas9) genome-editing vector (ATUM, Newark, California, USA). C6244-R cells were transfected with the gRNA containing Cas9 plasmids by electroporation using an Amaxa Nucleofector Device (Lonza, Basel, Switzerland) using Nucleofector solution T and setting T-020. Transfection was monitored by green fluorescent protein (GFP) expression and single GFP positive/DAPI-negative cells were sorted in to 96-well plates using a 100 µm nozzle on a BD FACSARIA III cell sorter (BD Biosciences, Oxford, UK). Clones of interest were identified by western blot screening for absence of p57^KIP2^. Guides 1 and 2 (5ʹ-TCCGCAGCACATCCACGATG-3ʹ and 5ʹ-GTGGGACCTTCCCAGTTACT-3ʹ, respectively) both produced clones with no p57^KIP2^ expression, as well as control clones, which still expressed p57^KIP2^.

Genomic DNA was extracted from C6244-R (control untransfected) and p57 WT1, p57 KO1 and p57 KO2 (generated using guide 2). Cells were lysed with Tail Lysis Buffer (50 mM Tris pH 8.0, 5 mM EDTA, 0.25% SDS, 200 mM NaCl) and 200 μg mL^−1^ proteinase K added to the lysate prior to incubation at 55 °C overnight. An equivalent volume of phenol/chloroform/isoamyl alcohol 25:24:1 (v/v) saturated with 10 mM Tris pH 8.0, 1 mM EDTA (Sigma-Aldrich, Dorset, UK) was then added and tubes mixed by inversion. After centrifugation, the DNA-containing aqueous phase was collected and precipitated by adding 0.8 volumes of 100% isopropanol (Sigma-Aldrich, Dorset, UK). The precipitated DNA was pelleted by centrifugation, washed with 70% (v/v) ethanol and solubilized in nuclease-free water.

Genomic DNA flanking the CRISPR guide binding site was amplified by PCR using OneTaq (NEB, Hitchin, UK) with GC reaction buffer (NEB, Hitchin, UK) and the following primers: 5ʹ-AGAAGAGTCCACCACCGGAC-3ʹ; 5ʹ-CGGACAGCTTCTTGATCGCC-3ʹ. This generated a 1 kb fragment, which was then cloned into the TOPO-TA cloning vector (Thermo Fisher Scientific, Loughborough, UK) following the manufacturer’s instructions. The resulting constructs were used to transform chemically competent DH5α (NEB, Hitchin, UK), 4–5 of the resulting clones derived from DNA for each cell line were sent for sequencing (Genewiz, Bishop’s Stortford, UK). Uncropped western blot images are shown for knockout of *CDKN1C*/p57^KIP2^ (Fig. 4c) in the Supplementary Data 1.

### siRNA-mediated knockdown

For transient siRNA in COLO205/C6244-R cells, 4 × 10^6^ cells were resuspended in 100 μL of Nucleofector solution T (Lonza, Basel, Switzerland) and mixed with 6 μg of the non-targeting (NT) siRNA pool (5ʹ-UAAGGCUAUGAAGAGAUAC-3ʹ, 5ʹ-AUGUAUUGGCCUGUAUUAG-3ʹ, 5ʹ-AUGAACGUGAAUUGCUCAA-3ʹ, 5ʹ-UGGUUUACAUGUCGACUAA-3ʹ) or siRNA targeting *CDKN1A* (encoding p21^CIP1^; 5ʹ-CUGUACUGUUCUGUGUCUU-3ʹ). Cells were transfected by electroporation with an Amaxa Nucleofector Device (Lonza, Basel, Switzerland) and protocol T-020. Cells were then plated out for subsequent treatment 24 h post-transfection as detailed in the Figure legends.

For transient siRNA in HCT116/H6244-R and LoVo/L6244-R cells, oligonucleotides targeting the following sequences were used: non-targeting (5ʹ-UAAGGCUAUGAAGAGAUAC-3ʹ, 5ʹ-AUGUAUUGGCCUGUAUUAG-3ʹ, 5ʹ-AUGAACGUGAAUUGCUCAA-3ʹ, 5ʹ-UGGUUUACAUGUCGACUAA-3ʹ); si*SNAI1* (5ʹ-ACUCAGAUGUCAAGAAGUA-3ʹ, 5ʹ-GCAAAUACUGCAACAAGGA-3ʹ, 5ʹ-GCUCGGACCUUCUCCCGAA-3ʹ, 5ʹ-GCUUGGGCCAAGUGCCCAA-3ʹ); si*SNAI2* (5ʹ-GGACACACAUACAGUGAUU-3ʹ, 5ʹ-UAAAUACUGUGACAAGGAA-3ʹ, 5ʹ-GAAUGUCUCUCCUGCACAA-3ʹ, 5ʹ-GAAUCUGGCUGCUGUGUAG-3ʹ); si*ZEB1* (5ʹ-GAACCACCCUUGAAAGUGA-3ʹ, 5ʹ-GAAGCAGGAUGUACAGUAA-3ʹ, 5ʹ-AAACUGAACCUGUGGAUUA-3ʹ, 5ʹ-GAUAGCACUUGUCUUCUGU-3ʹ) (GE Healthcare Life Sciences, Buckinghamshire, UK). siRNA oligonucleotides were combined with Opti-MEM (Gibco, Thermo Fisher Scientific, Paisley, UK) and incubated for 5 min at room temperature (RT). At the same time, an equivalent volume of DharmaFECT2 (GE Healthcare Life Sciences, Buckinghamshire, UK)/Opti-MEM (1:60 ratio) mix was incubated for 5 min at RT. siRNA and DharmaFECT2 mixes were then combined and incubated for 20 min at RT. siRNA/DharmaFECT2 complexes were added to culture dishes and cells seeded in penicillin/streptomycin-free medium at the required density. For H6244-R cells, final siRNA concentrations were 15 nM *SNAI1* siRNA, 50 nM *SNAI2* siRNA and 15 nM *ZEB1* siRNA. For L6244-R cells, final siRNA concentrations were 10 nM *SNAI1* and 50 nM *ZEB1*. Cells were then treated 24 h post-transfection as indicated in the Figure legends. Uncropped western blot images are shown for Fig. 8f (knockdown of SNAI1, SNAI2 or ZEB1) in Supplementary Data 2.

### Xenograft experiments

For compound formulation, hydroxypropyl methylcellulose (HPMC) was prepared by adding 5% (w/v) Methocel E4M Premium (Colorcon, Dartford, UK) and 1% (v/v) Polysorbate 80 (Sigma-Aldrich, Dorset, UK) to water. Selumetinib (AstraZeneca, Cambridge, UK) was ball milled in 0.5% (w/v) Methocel E4M Premium/0.1% (v/v) Polysorbate 80 overnight. Selumetinib suspension was stored in the dark and discarded after 7 days.

COLO205 and C6244-R cells were grown to ~ 80% confluence in RPMI 1640 supplemented with 10% (v/v) foetal bovine serum, 2 mM glutamine (Gibco, Thermo Fisher Scientific, Paisley, UK) and 1 µM selumetinib for C6244-R cells. On the day of injection, cells were trypsinized, centrifuged and resuspended at a concentration of 3 × 10^7^ cells mL^−1^ in cold RPMI-1640 containing 50% (v/v) Matrigel (BD Biosciences, Oxford, UK) and 1 µM selumetinib. In all, 10 mg kg^−1^ or 25 mg kg^−1^ selumetinib or vehicle (0.5% (w/v) HPMC/0.1% (v/v) Polysorbate 80) were administered to groups of 9–10 (Fig. 5o) or 21 (Supplementary Fig. 7n) female athymic mice randomized by body mass (18 g or higher) by oral gavage twice daily with an 8 h interval. The following day mice received selumetinib or vehicle and 2 h later were injected subcutaneously with 3 × 10^6^ C6244-R cells suspended in 50% (v/v) Matrigel/cold RPMI-1640 containing 1 µM selumetinib. Mice continued to be weighed and dosed twice daily. In addition, a separate control group (*n* = 10 athymic females) were injected with COLO205 cells (parental). All xenograft sizes were recorded twice weekly using calipers as soon as tumours were palpable until meeting pre-established humane endpoint criteria including loss of body mass ( >17–20% at anytime) or general signs of sickness such as piloerection. Operators were not blinded during these studies.

HCT116 and H6244-R cells were grown to ~ 80% confluence in DMEM, 10% (v/v) foetal bovine serum, penicillin (100 U mL^−1^), streptomycin (100 mg mL^−1^) and 2 mM glutamine (Gibco, Thermo Fisher Scientific, Paisley, UK), supplemented with 2 µM selumetinib for H6244-R cells. On the day of injection, cells were trypsinized, centrifuged and resuspended at a concentration 1 × 10^7^ cells mL^−1^ in cold PBS. In total, 50 mg kg^−1^ selumetinib (determined by tolerance testing) was administered to 32 female SCID (NOD.CB17-*Prkdc*^*scid*^/J; The Jackson Laboratory, Maine, US) mice aged 98 days ± 26 days (mean ± SD) by oral gavage. The following day mice received 50 mg kg^−1^ selumetinib and 2 h later were anesthetized and injected subcutaneously with 3 × 10^6^ H6244-R cells suspended in 50% (v/v) Matrigel/cold PBS. Mice continued to be weighed and dosed daily. Nine days after transplantation, mice were paired according to xenograft size and for each pair, one mouse was randomly assigned into group A (*n* = 16; tumour volume 0.0886 ± 0.0655 cm^3^, mean ± SD) and another into group B (*n* = 16; tumour volume 0.0777 ± 0.0457 cm^3^, mean ± SD): the next day selumetinib dosing continued in group A but drug was withdrawn for group B, which received vehicle only (0.5% (w/v) HPMC/0.1% (v/v) Polysorbate 80). In addition, a small control group (*n* = 3 SCID females) were injected with HCT116 cells (parental) on the same day as H6244-R injections, and HCT116 xenografts were allowed to grow for 14 days before administration of 50 mg kg^−1^ selumetinib. All xenograft sizes were recorded twice weekly using calipers from 7 days after transplantation until reaching pre-established humane endpoint criteria including xenograft size (L × W > 1.2 cm^2^), loss of body weight (>10% in a week) or general signs of sickness such as piloerection. Mice were sacrificed and xenografts, lungs and livers (the main organs for HCT116 metastases) placed in a cassette and submersed in 10% neutral buffered formalin (NBF; CellPath Ltd, Powys, UK) for histological analyses. Two animal technicians performed all xenograft measurements and were not blinded to dosing groups. The histologist was blinded to dosing group.

For all xenograft experiments, tumour length (L) and width (W) were measured using calipers and tumour volume calculated using the formula for the volume of a prolate spheroid V = 4/3π(L/2)(W/2)².

For histological analysis, tissues were fixed in 10% NBF for 24–48 h before dehydrating by incubating for 3 min in increasing concentrations of ethanol (50%, 70%, 80%, 95%, 100%) and embedded in paraffin. Samples were sectioned (4 µm) on a cryostat (Leica Biosystems, Newcastle, UK), mounted on glass slides, dewaxed in Xylene (Aquascience, Lowestoft, UK) and rehydrated by incubation for 3 min in decreasing concentrations (100%, 80%, 70%, 50%, water) of ethanol before staining with Haematoxylin Gill III (Leica Biosystems, Newcastle, UK) and Eosin (Leica Biosystems, Newcastle, UK). A trained histopathologist scored tumours for invasiveness and inspected organs for the presence of metastases.

### Preparation of cell lysates for SDS-PAGE and western blotting

Culture medium from cells growing on dishes was either removed or non-adherent cells and cellular material recovered from the medium by centrifugation (1300 × *g* at RT for 3 min). Cells were washed with PBS and then lysed for 5 min with ice-cold TG lysis buffer (20 mM Tris-HCl (pH 7.4), 137 mM NaCl, 1 mM EGTA, 1% (v/v) Triton X-100, 10% (v/v) glycerol, 1.5 mM MgCl_2_, 50 mM NaF, 1 mM Na_3_VO_4_, 5 μg mL^−1^ aprotinin, 10 μg mL^−1^ leupeptin, 1 mM phenylmethylsulfonyl fluoride (PMSF)). Lysates were detached and collected using a cell scraper and transferred to pre-chilled tubes. Lysates were then cleared of non-soluble material by centrifugation (13,000 × *g* at 4 °C for 10 min), and supernatant protein concentration determined by Bradford protein assay. For Bradford assay, 5 µL cleared lysate was mixed with 795 µL ultra-pure water and 200 µL Bradford reagent (Bio-Rad, Watford, UK), and absorbance at 595 nm measured using a PHERAstar FS plate reader (BMG Labtech, Aylesbury, UK). Samples were prepared for sodium dodecyl sulfate-polyacrylamide gel electrophoresis (SDS-PAGE) by boiling in 1 × Laemmli sample buffer (50 mM Tris-HCl (pH 6.8), 2% (w/v) SDS, 10% (v/v) glycerol, 1% (v/v) β-mercaptoethanol, 0.01% (w/v) bromophenol blue).

### Preparation of cell lysates for SDS-PAGE and mass spectrometry

Culture medium from cells growing on dishes was either removed or non-adherent cells and cellular material recovered from the medium by centrifugation (1300 × *g* at RT for 3 min). Cells were washed with PBS and then lysed for 5 min with ice-cold RIPA lysis buffer (20 mM Tris-HCl (pH 7.4), 137 mM NaCl, 1 mM EGTA, 1% (v/v) Triton X-100, 0.1% (w/v) SDS, 1% (w/v) sodium deoxycholate, 10% (v/v) glycerol, 1.5 mM MgCl_2_, 50 mM NaF, 1 mM Na_3_VO_4_, 5 μg mL^−1^ aprotinin, 10 μg mL^−1^ leupeptin, 1 mM PMSF, 0.025 U mL^−1^ benzonase (Sigma-Aldrich, Dorset, UK)). Lysates were detached and collected using a cell scraper and transferred to pre-chilled tubes. Protein concentration was determined by BCA protein assay (Pierce BCA Protein Assay Kit, Thermo Fisher Scientific, Loughborough, UK) and absorbance measured at 562 nm using a PHERAstar FS plate reader (BMG Labtech, Aylesbury, UK). Samples were prepared for SDS-PAGE by boiling for 5 min in 1 × Laemmli sample buffer (50 mM Tris-HCl (pH 6.8), 2% (w/v) SDS, 10% (v/v) glycerol, 1% (v/v) β-mercaptoethanol, 0.01% (w/v) bromophenol blue).

### SDS-PAGE and western blotting

Lysates were separated by SDS-PAGE (Mighty small II gel apparatus, Hoefer, Massachusetts, USA). Polyacrylamide gels consisted of a resolving phase of 8–16% (w/v) acrylamide (37.5:1 acrylamide:bisacrylamide, 2.7% crosslinker; Bio-Rad, Watford, UK), 0.375 M Tris-HCl (pH 8.8), 0.2% (w/v) SDS (Bio-Rad, Watford, UK), 0.1% (w/v) ammonium persulphate, 0.1% (v/v) tetramethylethylenediamine (TEMED; Bio-Rad, Watford, UK) and a stacking phase of 4.5% (w/v) acrylamide (37.5:1 acrylamide:bisacrylamide, 2.7% crosslinker), 0.125 M Tris-HCl (pH 6.8), 0.2% (w/v) SDS (Bio-Rad, Watford, UK), 0.1% (w/v) ammonium persulphate, 0.125% (v/v) TEMED. Gels were run using running buffer (0.2 M glycine, 25 mM Tris, 0.1% (w/v) SDS) and a current of 15 mA per gel for 3–4 h. Gels were then blotted by wet transfer (Bio-Rad, Watford, UK) to methanol activated polyvinylidene difluoride (PVDF) membrane (Immobilon-P Membrane, Merck Millipore, Watford, UK) using transfer buffer (0.2 M glycine, 25 mM Tris, 20% (v/v) methanol) and a current of 300 mA for 90 min. Membranes were blocked in 5% milk/ Tris buffered saline and Tween 20 (TBST) (5% (w/v) non-fat powdered milk, 10 mM Tris-HCl (pH 7.6), 150 mM NaCl, 0.1% (v/v) Tween-20) for 1 h at RT. Membranes were incubated with primary antibodies against BID, CDH1 (E-cadherin), CDH2 (N-cadherin), MEK1/2, p18^INK4C^, p57^KIP2^ C-term, PARP, phospho-ERK1/2 T185 Y187/T202 Y204, phospho-GSK3α/β S21/S9, phospho-MEK1/2 S217/S221, phospho-PKB/AKT T308, phospho-PKB/AKT S473, phospho-RB S795, phospho-RSK S380, phospho-S6K T389, PKB/AKT, RSK, SNAI1 (Snail), SNAI2 (Slug), VIM, ZEB1 (Cell Signaling Technology, NEB, Hitchin, UK), BRAF, CCNA (cyclin A), FRA1, p15^INK4B^, p16^INK4A^, p19^INKD^, TWIST1 (Santa Cruz Biotechnology, Dallas, USA), BIM, CCND1 (cyclin D1), KRAS, NOXA, p27^KIP1^, p53 (Merck Millipore, Watford, UK), ERK1, p21^CIP1^ (BD Biosciences, Oxford, UK), β-actin, LC3B (Sigma-Aldrich, Dorset, UK), and p57^KIP2^ N-term (Abcam, Cambridge, UK) diluted as recommended in 5% milk/TBST or 5% bovine serum albumin (BSA)/TBST overnight at 4 °C with agitation. Membranes were then washed in TBST for 4 × 10 min, and incubated with horseradish peroxidase (HRP)-conjugated secondary antibodies (Bio-Rad, Watford, UK) diluted 1:3000 in 5% milk/TBST for 1 h at RT. Membranes were again washed for 4 × 10 min in TBST. Detection was performed using Amersham ECL Western Blotting Detection Reagent (GE Healthcare Life Sciences, Buckinghamshire, UK), Clarity Western ECL Substrate (Bio-Rad, Watford, UK) or Immobilon Western Chemiluminescent HRP Substrate (Merck Millipore, Watford, UK), X-ray film and Compact X4 film developer (Xograph, Gloucestershire, UK). Uncropped western blot images are shown for Fig. 4c (knockout of *CDKN1C*/p57^KIP2^) and Fig. 8f (knockdown of SNAI1, SNAI2 or ZEB1) in Supplementary Data 1 and Supplementary Data 2, respectively.

### Mass spectrometry

Samples run by SDS-PAGE were stained with Coomassie (SimplyBlue SafeStain, Thermo Fisher Scientific, Loughborough, UK) and gel pieces covering the mass range 37–46 kDa excised and transferred to a microfuge tube (Eppendorf LoBind tubes, Sigma-Aldrich, Dorset, UK). Gel pieces were destained by washing in aqueous solution (fresh 50 mM NH_4_HCO_3_) for 5 min and then twice with organic solution (fresh 50 mM NH_4_HCO_3_/acetonitrile 1:1). Destained gel pieces were then incubated in 10 mM dithiothreitol (DTT) in aqueous 50 mM NH_4_HCO_3_ for 1 h at 50 °C and then cooled to RT and DTT discarded. Iodoacetamide (50 mM iodoacetamide in aqueous 50 mM NH_4_HCO_3_) was then added and incubated for 1 h in the dark at RT with occasional mixing. The supernatant was then discarded and gel pieces washed with aqueous solution for 5 min, and then with organic solution twice for 5 min. Gel pieces were then dried completely in a centrifugal vacuum concentrator. Dried gel pieces were rehydrated in trypsin solution (50 µL, 1 ng µL^−1^ trypsin in 25 mM ammonium bicarbonate, 0.01% octyl-β-glucoside) containing 1 fmol of each ERK1 or ERK2 heavy peptide standard (Table 1). A further 50 µL of 25 mM ammonium bicarbonate was added to each sample for overnight digestion at 30 °C. The digestion was stopped by the addition of 10 µL of 10% formic acid, then 100 µL acetonitrile was added and the sample sonicated for 15 min. The supernatant was transferred to a fresh tube and gel pieces sonicated for 15 min with a further 50 µL of acetonitrile. The combined supernatants were dried in a vacuum centrifuge then resuspended in 100 µL 0.1% trifluoroacetic acid (TFA) and stored at −20 °C prior to nano liquid chromatography tandem mass spectrometry (nLC-MS/MS) analysis. All chemicals were from Sigma-Aldrich, Dorset, UK.

**Table 1:** Heavy peptide standard sequences for quantification of phosphorylated and non-phosphorylated ERK1 and ERK2 by mass spectrometry

Species^a^	Peptide^b^	m/z	charge	t start [min]	t stop [min]	Max injection time [ms]	AGC target
ERK1_L_NP	GQPFDVGPR	486.7485	2	0	19	250	100,000
ERK1_H_NP	GQPFDVGPR_H_	491.7527	2	0	19	250	100,000
ERK2_L_NP	GQVFDVGPR	487.7563	2	0	19	250	100,000
ERK2_H_NP	GQVFDVGPR_H_	492.7605	2	0	19	250	100,000
ERK1_L_P	IADPEHDHTGFLpTEpYVATR	777.9943	3	19	32	500	50,000
ERK1_H_P	IADPEHDHTGFLpTEpYVATR_H_	781.3304	3	19	32	100	50,000
ERK2_L_P	VADPDHDHTGFLpTEpYVATR	768.6505	3	19	32	500	50,000
ERK2_H_P	VADPDHDHTGFLpTEpYVATR_H_	771.9866	3	19	32	100	50,000

AGC, automatic gain control

^a^ Peptide features: *L* light, *H* heavy, *NP* non-phospho, *P* phospho

^b^ Peptide sequences: *pT* phospho-threonine, *pY* phospho-tyrosine, *R*_*H*_ heavy isotope arginine

Quantitative analysis of ERK1 and ERK2 peptides was performed on the UltiMate 3000 nanoUHPLC interfaced via an EASY-Spray nano electrospray ion source to an Orbitrap Fusion Lumos mass spectrometer (all from Thermo Fisher Scientific, Loughborough, UK). Samples were loaded onto a precolumn (0.1 × 20 mm Acclaim PepMap100, 5 µm particles) in 0.1% TFA at a flow rate of 8 µL min^−1^. Peptides were eluted from the precolumn and separated on an analytical column (0.075 × 500 mm, EASY-Spray PepMap RSLC, 2 µm particles) with a 16.7 min linear gradient from 14 to 43% solvent B (80% acetonitrile, 0.1% formic acid), at 300 nL min^−1^ and 40 °C. ERK1 and ERK2 peptide ions were isolated in the quadrupole with a 1.2 Th window, and fragmented by higher energy collisional dissociation (HCD) with a relative collision energy of 30%. Fragment ions were analyzed in the Orbitrap analyzer over the m/z range 120–1200 Th with a 15,000 resolution setting. Quantitative information was extracted from the raw data using Skyline software (MacCoss Lab, University of Washington, USA). Each independent experiment was performed in technical triplicate.

### Multiplexed enzyme-linked immunosorbent assay (ELISA)

At the end of treatment, cell culture supernatant medium was harvested and subjected to multiplexed ELISA analysis to determine the concentration of selected cytokines (Human ProInflammatory 7-Plex Tissue Culture Kit or Human ProInflammatory-4 II Tissue Culture Kit, Meso Scale Discovery, Maryland, US) following the manufacturer’s instructions. Results were normalized to total cellular protein as determined by Bradford assay.  Each independent experiment was performed in technical triplicate.

### Cell cycle analysis by flow cytometry

Culture medium from subconfluent cells growing in plates was collected, adherent cells trypsinized and cells and media then recombined. Cells were pelleted by centrifugation (500 × *g*, RT, 5 min) and resuspended in 0.2 mL PBS. Cells were fixed in 70% (v/v) ethanol/PBS at 4 °C for at least 30 min. Samples were then centrifuged (500 × *g*, 4 °C, 5 min), washed with PBS, and resuspended in 0.25 mL PBS containing 25 μg RNase A (Sigma-Aldrich, Dorset, UK) and 12.5 μg of the DNA intercalating agent propidium iodide (PI; Sigma-Aldrich, Dorset, UK). Following 30 min incubation at 37 °C, samples were passed through a 25-gauge needle to minimize cell clumping. Cell cycle profiles were acquired with a FACS Calibur or LSRII flow cytometer (BD Biosciences, Oxford, UK) to measure binding of PI to DNA, and counting 10,000 cells per sample. Each independent experiment was performed with cell culture triplicates. Data were analyzed using CellQuest (BD Biosciences, Oxford, UK) or FlowJo (FlowJo LLC, Oregon, USA) software packages and sub-G1, G1, S and G2-M cell cycle phases gated.

### Flow cytometric analysis of p-ERK1/2 and EdU

Cells were treated as described in the Figure legends and 1 h prior to harvest incubated with 10 μM EdU (Click-iT EdU Alexa Fluor 647 Flow Cytometry Assay Kit, Thermo Fisher Scientific, Loughborough, UK). Cells, subconfluent at the point of harvest, were fixed with 4% paraformaldehyde/PBS (Santa Cruz Biotechnology, Dallas, USA) for 15 min at RT and permeabilized with 100% ice-cold methanol while vortexing gently and incubated on ice for 30 min. EdU was detected following the manufacturer’s instructions. Cells were then washed in PBS and stained with p-ERK1/2 (T202 and Y204/T185 and Y187) antibody conjugated to Alexa Fluor 488 (rabbit monoclonal #4344, Cell Signaling Technology, NEB, Hitchin, UK) diluted 1:100 in 0.5% (w/v) BSA/PBS for 1 h at RT in the dark. Cells were washed three times in 0.5% (w/v) BSA/PBS and resuspended in 1 µg mL^−1^ of DAPI (Sigma-Aldrich, Dorset, UK) in PBS to allow assessment of cell cycle profile. Phospho-ERK1/2 and EdU co-staining was assessed with a FACS LSRII flow cytometer (BD Biosciences, Oxford, UK), counting 10,000 cells per sample using no EdU and no phospho-ERK1/2 primary antibody samples as gating controls. Data were analyzed using FlowJo software (FlowJo LLC, Oregon, USA).

### Co-culture of COLO205 and C6244-R cells

COLO205 and C6244-R cells in suspension were pelleted (500 × *g*, RT, 3 min), washed once in PBS and then resuspended in PBS to 1 × 10^6^ cells per mL. COLO205 cells were then incubated with 1 µM CellTrace far red fluorescent stain (Thermo Fisher Scientific, Loughborough, UK), and C6244-R cells with 5 µM CellTrace violet fluorescent stain, for 10 min. Warm fresh complete medium was then added to five times the staining volume and incubated for 5 min at 37 °C, 5% (v/v) CO_2_. Cells were then pelted (500 × *g*, RT, 3 min), resuspended in warm complete media and incubated for 10 min at 37 °C, 5% (v/v) CO_2_ before pelleting again and resuspending cells in warm complete medium to a concentration of 1 × 10^6^ cells per mL. COLO205 and C6244-R cells were then combined at a 50:50 ratio, plated and treated with 0.01 µM–10 µM selumetinib as indicated. At the point of harvest, culture medium from subconfluent cells was collected, adherent cells trypsinized and cells and media then recombined. Cells were pelleted by centrifugation (500 × *g*, RT, 5 min) and resuspended in 0.2 mL PBS. The ratio of COLO205 to C6244-R cells was then determined at each selumetinib concentration every day for 7 days by flow cytometry using a FACS LSRII flow cytometer (BD Biosciences, Oxford, UK) and 405/50 and 640/10 band pass filters.

### SA-β-gal assay

Following the treatments indicated in the Figure legends, subconfluent cells were washed three times in PBS, fixed with 4% parafomaldehyde (Santa Cruz Biotechnology, Dallas, USA) for 5 min, washed twice with PBS and incubated with fresh β-galactosidase staining solution (40 mM citric acid/sodium phosphate buffer pH 6, 5 mM K_4_[Fe(CN)_6_].3H_2_O, 5 mM K_3_[Fe(CN)_6_], 150 mM sodium chloride, 2 mM magnesium chloride and 1 mg mL^−1^ X-gal in distilled water) overnight at 37 °C in a humidified atmosphere. The following day, cells were washed twice in PBS, once in methanol and allowed to air dry. Images of SA-β-gal-stained cells were taken on a Zeiss Axiovert 100 microscope (Carl Zeiss Ltd, Cambridge, UK).

### Proliferation assay with [^3^H-methyl]thymidine

Cells were seeded in 24-well plates and 24 h later washed once with fresh medium only and then treated with fresh medium containing the appropriate drug concentrations. Cells were incubated at 37 °C, 5% (v/v) CO_2_ for a further 24 h, with 5 μM thymidine containing 0.5 μCi [^3^H-methyl]thymidine (Perkin Elmer, Buckinghamshire, UK) added for the final 6 h. Medium was aspirated and cells, which were subconfluent, fixed in 5% (w/v) trichloroacetic acid (TCA) at 4 °C overnight. Fixed cells were then washed with water, solubilized with 0.5 mL 0.1 M NaOH, transferred to scintillation vials containing 4 mL scintillation fluid (0.4% (w/v) TCA; Perkin Elmer, Buckinghamshire, UK), and counted on a Packard Tricarb 4000 series scintillation counter (Perkin Elmer, Buckinghamshire, UK). Each independent experiment was performed with cell culture triplicates.

### Cell count proliferation assay

Parental and resistant cells were plated in six-well plates, in triplicate for each time point and condition, and 24 h later washed with fresh medium only followed by treatment with or without selumetinib as indicated in the Figure legends. On the day of treatment (day 0) and on each subsequent day for a total of 9–10 days, cells were trypsinized and counted using a haemocytometer (Brand, Wertheim, Germany) to give the number of cells per well and proliferation rate. Each independent experiment was performed with cell culture triplicates.

### Proliferation assay with BrdU

Cells were plated in six-well plates and 24 h later washed with fresh medium only, followed by treatment with fresh medium containing the appropriate drug concentrations. One hour prior to harvest, cells were incubated with 10 μM BrdU (Thermo Fisher Scientific, Loughborough, UK) before fixing with 70% (v/v) ethanol/PBS at 4 °C for at least 10 min. Cells were centrifuged (500 × *g*, 4 °C, 5 min) and washed twice in PBS, DNA denatured with 1.5 M HCl for 30 min and again centrifuged (500 × *g*, 4 °C, 5 min) and washed twice in PBS. Cells were then blocked in 0.5% (w/v) BSA/PBS for 10 min at RT before addition of anti-BrdU primary antibody (Cell Signaling Technology, NEB, Hitchin, UK) diluted 1:200 for 1 h at RT. Following centrifugation (500 × *g*, 4 °C, 5 min) and washing twice in 0.5% (w/v) BSA/PBS, cells were incubated with anti-mouse fluorochrome-conjugated secondary antibody (anti-mouse Alexa Fluor 488, Thermo Fisher Scientific, Loughborough, UK) diluted 1:200 for 30 min at RT. Cells were then centrifuged (500 × *g*, 4 °C, 5 min) and washed in 0.5% (w/v) BSA/PBS three times, and finally resuspended in 0.2 mL PBS. BrdU-positive cells were assessed with a FACS LSRII flow cytometer (BD Biosciences, Oxford, UK), counting 10,000 cells per sample using no BrdU samples as gating controls. Data were analyzed using FlowJo software (FlowJo LLC, Oregon, USA).

### High-content microscopy and analysis of EdU incorporation, p-ERK1/2 and p-RSK levels

Cells were seeded in 96-well black wall imaging plates (ViewPlate-96, Perkin Elmer, Buckinghamshire, UK) and treated 24 h later as indicated in the Figure legends. For EdU incorporation analysis, cells were incubated with 10 μM EdU (Thermo Fisher Scientific, Loughborough, UK) for the last 1 h of treatment, except in background control wells where no EdU was added. Cells were then harvested and fixed with 4% paraformaldehyde/PBS (Santa Cruz Biotechnology, Dallas, USA), washed once with PBS and then permeabilized with 100% methanol for 10 min at −20 °C. Cells were then washed in PBS and EdU click reaction performed following the manufacturer’s instructions (Click-iT EdU Alexa Fluor 647 HCS Assay, Thermo Fisher Scientific, Loughborough, UK). For detection of p-ERK1/2 and p-RSK, either in isolation or in conjunction with prior EdU incorporation and click reaction, cells were blocked for 1 h with 2% BSA/PBS at RT, followed by incubation with p-ERK1/2 antibody (rabbit monoclonal #4370, Cell Signaling Technology, NEB, Hitchin, UK) or p-RSK (rabbit monoclonal #11989, Cell Signaling Technology, NEB, Hitchin, UK) diluted 1:500 (p-ERK1/2) or 1:200 (p-RSK) in 2% BSA/PBS at 4 °C overnight. For background control wells, 2% BSA/PBS without p-ERK1/2 or p-RSK antibody was added. Cells were washed three times with PBS, and then incubated with anti-rabbit secondary antibodies (goat anti-rabbit Alexa Fluor 488 or goat anti-rabbit Alexa Fluor 568, Thermo Fisher Scientific, Loughborough, UK) diluted 1:300 in 2% BSA/PBS containing 1 µg mL^−1^ of DAPI (Sigma-Aldrich, Dorset, UK) for 1 h at RT. Cells were washed four times with PBS and stored in 100 μL PBS before imaging. Cells were imaged using an IN Cell Analyzer 6000 microscope (GE Healthcare Life Sciences, Buckinghamshire, UK) using a 10 × objective lens, and typically imaging 2000–10,000 individual cells (in six fields) per well. Image analysis to determine the mean signal intensity per cell or percent cells staining positively was performed using IN Cell Analyzer software (GE Healthcare Life Sciences, Buckinghamshire, UK).

### Cell viability assay with Sytox Green

Cells were plated in 96-well plates in triplicate for each condition and treated as indicated in the Figure legends. At the end of treatment, a dead cell read was performed by adding the DNA-binding dye Sytox Green (Thermo Fisher Scientific, Loughborough, UK) at a final concentration of 0.15 µM directly to the media, incubating for 1 h in the dark and then reading the fluorescence intensity (Sytox Green excitation: 504 nm, emission: 523 nm) on PHERAstar FS plate reader (BMG Labtech, Aylesbury, UK). Cells were then permeabilized with 0.03% (w/v) saponin (Sigma-Aldrich, Dorset, UK) overnight at RT, and read as before. Both Sytox Green and saponin were dissolved in TBS/EDTA buffer (0.1 M Tris, 0.15 M NaCl, 5 mM EDTA, pH 7.0). Relative live cell proportion was calculated by subtracting the dead read fluorescence intensity from the total read fluorescence intensity. Each independent experiment was performed with cell culture triplicates.

### Brightfield phase contrast microscopy

Brightfield phase contrast imaging was performed using an Olympus BX41 Microscope (Olympus, Southend-on-Sea, UK).

### Immunocytochemistry

Cells were seeded in six-well plates containing sterilized glass coverslips and treated as indicated in the Figure legends. Coverslips were washed three times with PBS and fixed with 4% (v/v) paraformaldehyde/PBS for 15 min. Following three washes in PBS, cells were permeabilized with 0.1% (v/v) Triton X-100/PBS for 10 min. Cells were washed three times in PBS and blocked for 1 h in BSA blocking solution (2% (w/v) BSA, 0.02% (w/v) sodium azide in PBS) at RT. Cells were incubated with primary antibodies to CDH1 (E-cadherin; rabbit monoclonal #3195, Cell Signaling Technology, NEB, Hitchin, UK) or VIM (rabbit monoclonal #5741, Cell Signaling Technology) diluted 1:200 (CDH1) or 1:100  (VIM) in BSA blocking solution (50 μl per coverslip) at 4 °C overnight. Cells were washed three times in BSA blocking solution and then incubated with anti-rabbit fluorochrome-conjugated secondary antibody (anti-rabbit Alexa Fluor 488, Thermo Fisher Scientific, Loughborough, UK) diluted in BSA blocking solution 1:500 (50 μl per coverslip) for 1 h at RT in the dark. Cells were washed three times with BSA blocking solution and twice in PBS, before being mounted onto microscope slides with VectaShield Antifade Mounting Medium with DAPI (Vector Laboratories, Peterborough, UK). Slides were left to dry for 1 h at RT in the dark and imaged using FV1000 confocal microscope (Olympus, Southend-on-Sea, UK) or Nikon A1-R confocal microscope (Nikon Instruments, Amsterdam, The Netherlands). The colours displayed in images are pseudo-colours.

### Cell volume measurement

To measure the volumes of COLO205, HT29, HCT116 and LoVo cells ± selumetinib and their selumetinib-resistant derivatives ± selumetinib, cells were plated in their normal growth medium in chamber slides (Thistle Scientific, Glasgow, UK) and 24 h later washed with media only and treated with or without selumetinib for a further 72 h. Cells were then incubated for 5 min with CellMask Orange Plasma Membrane Stain (Thermo Fisher Scientific, Loughborough, UK), which was added directly to the media at a dilution of 1:1000. Cells were then imaged using an Andor Revolution confocal spinning disk system (Andor, Oxford Instruments, Oxfordshire, UK) taking 100 images per cell, over a depth of 50 µm and a step interval of 0.5 µm. At least 300 cells were imaged per condition and cell volumes were determined using Imaris imaging analysis software (Bitplane, Oxford Instruments, Oxfordshire, UK).

### Undirected motility assay

Cells were treated as indicated in the Figure legends for a total of 9 days. Five days after treatment initiation, cells were split and seeded at 2 × 10^4^ cells per well of a six-well plate so as to be subconfluent on day 9 after treatment initiation. Cells maintained at 37 °C and 5% (v/v) CO_2_ were then photographed every 10 min for 16 h using an Olympus CellR microscope (Olympus, Southend-on-Sea, UK) or Nikon Eclipse Ti-E microscope (Nikon Instruments, Amsterdam, The Netherlands). At least 300 cells per condition, across two independent experiments, were tracked using the Manual Tracking ImageJ plug in (https://imagej.net/Manual_Tracking), and data analyzed with the ibidi GmbH Chemotaxis and Migration Tool (ibidi GmbH, Munich, Germany).

### Wound-healing assay

Cells were treated as indicated in the Figure legends for a total of 9 days. Five days after treatment initiation cells were split and seeded in to 6 cm dishes so as to reach confluence on the day of wounding (9 days after treatment initiation). Wounding was performed with a 200 μL pipette tip, and cells were then washed twice with fresh media before treatments were reapplied for the remainder of the experiment. Photographs were taken every 12 h in an identical location for a total of 60 h with an Olympus BX51 microscope (Olympus, Southend-on-Sea, UK) and wound areas remaining at each time point determined with Adobe Photoshop (Adobe Systems Europe Ltd, Maidenhead, UK).

### Chromosome harvest and FISH

To prepare metaphase chromosomes, cells were treated with 0.1 μg mL^−1^ colcemid for 1.5 h. Cells were then trypsinized, pelleted and resuspended in 0.5 mL media before being swelled by dropwise addition of 20 mL hypotonic solution (0.075 M KCl, 37 °C) under gentle agitation. Following incubation in hypotonic solution for 15 min at 37 °C, 1 mL of ice cold 3:1 fix (75% (v/v) methanol, 25% (v/v) acetic acid) was added dropwise and the suspension centrifuged at 500 × *g* for 5 min. The supernatant was aspirated leaving 0.5 mL in which cells were resuspended and cell clumps eliminated. 20 mL fresh ice cold 3:1 fix was then added dropwise under smooth agitation and cells incubated on ice for 5 min. Centrifugation, resuspension and fixing were then repeated a further two times, initially with 3:1 fix and then finally with 3:2 fix (60% (v/v) methanol, 40% (v/v) acetic acid). The resulting suspension was stored in 3:2 fix at −20 °C for at least 24 h before chromosomes were dropped on to slides. Metaphase spreads were prepared by dropping 20 μL of cell suspension onto 100 μL drops of sterile water on clean slides.

Chromosome 7 centromere probe was kindly provided by Suet-Feung Chin and Carlos Caldas (Department of Oncology, CRUK Cambridge Institute, University of Cambridge, UK). *BRAF* locus PAC clone RP5-1173P7 was selected from the laboratory of Paul Edwards 1 Mb BAC/PAC clone library. PAC DNA was extracted using Qiagen HiSpeed Plasmid Midi kit following the manufacturer’s instructions.

Chromosome 7 centromere probe was labelled with Spectrum Orange (Abbott Molecular, Illinois, USA) and PAC DNA with digoxygenin-11-dUTP (Sigma-Aldrich, Dorset, UK), using a nick translation kit (Abbott Molecular, Illinois, USA) following the manufacturer’s instructions. For each slide, 3 μg human Cot-1 DNA (Sigma-Aldrich, Dorset, UK), 200–500 ng centromere probe, and 50–100 ng PAC DNA were co-precipitated with 100% ethanol and dissolved for 1–3 h at 37 °C in 20 µL hybridization buffer (50% (v/v) deionized formamide, 10% (w/v) dextran sulphate, 0.3 M sodium chloride, 30 mM sodium citrate, 0.02% (w/v) Ficoll 400, 0.02% (w/v) polyvinylpyrrolidone, 40 mM sodium phosphate solution and 0.02% (w/v) BSA). Probe mixtures were denatured at 70 °C for 10 min, incubated on ice for 2 min, followed by incubation at 37 °C for 1 h. Slides with dropped chromosomes were dehydrated through an ethanol gradient (70%, 85%, 100%, 3 min each), pre-warmed to 37 °C, denatured at 70 °C in 70% (v/v) deinionized formamide/2 × SSC (0.3 M sodium chloride, 30 mM sodium citrate) for 1 min 20 s, quenched in ice-cold 70% (v/v) ethanol for 2 min, and again dehydrated through an ethanol gradient (70%, 85%, 100%, 3 min each). Probes were hybridized to slides overnight at 37 °C. Unbound probe was removed by washing with 2 × SSC at RT for 5 min, twice in 50% (v/v) formamide/0.5 × SSC (75 mM sodium chloride, 7.5 mM sodium citrate) at 42 °C for 5 min and twice in 0.5 × SSC (75 mM sodium chloride, 7.5 mM sodium citrate) at 42 °C for 5 min. For detection of digoxygenin-dUTP, slides were further washed in 0.05% BSA/4 × SST (0.05% (w/v) BSA, 0.6 M sodium chloride, 60 mM sodium citrate, 1 % (v/v) Tween) at RT, blocked in 3% BSA/4 × SST for 30 min at 37 °C, washed in 0.05% BSA/4 × SST for 1 min and incubated with sheep anti-digoxygenin-FITC (Sigma-Aldrich, Dorset, UK) for 30–45 min. Coverslips were then applied to slides using VectaShield Antifade Mounting Medium with DAPI (Vector Laboratories, Peterborough, UK). Slides were sealed with nail polish and stored at 4 °C in the dark. All probes were checked by hybridization to normal metaphase chromosomes. Slides were visualized using 83000 (Chroma Technology) and XF93 (Omega Optical) triple band pass filter sets mounted on a Nikon E800 fluorescence microscope fitted with a 100 W mercury lamp and a charge-coupled device (CCD) camera (Leica Biosystems, Newcastle, UK). Images were captured using CytoVision software (Leica Biosystems, Newcastle, UK) and the colours displayed are pseudo-colours.

### RNA extraction and quantitative reverse transcription PCR

At the of end treatment as indicated in the Figure legends, cells were lysed in TRI reagent (Sigma-Aldrich, Dorset, UK), typically 2 mL per 10 cm dish, and lysates mixed until a homogenous constituency was achieved. Lysates were transferred to sterile RNase free tubes, and 0.1 mL 1-bromo-3-chloropropane added per mL of lysate. The samples were mixed thoroughly and then allowed to stand for 5 min. Tubes were then centrifuged at 12,000 × *g* for 15 min at 4 °C, and the upper aqueous phase transferred to a fresh tube. In all, 0.5 mL 2-propanol per mL original lysate was then added, tubes mixed and allowed to stand for 5 min. Samples were then centrifuged at 12,000 × *g* for 10 min at 4 °C, and RNA pellets washed with 75% (v/v) ethanol. Pellets were resuspended in RNase free water and RNA concentrations and purity (A260:A280 ratio) determined with a Nanodrop 2000 spectrophotometer (Thermo Fisher Scientific, Loughborough, UK).

Reverse transcription PCR was performed using QuantiTect Reverse Transcription Kit (Qiagen, Manchester, UK) following the manufacturer’s instructions. mRNA levels were then determined by real-time PCR using SYBR Green PCR Master Mix (Thermo Fisher Scientific, Loughborough, UK) following the manufacturer’s instructions and CFX96 Real-Time PCR Detection System (Bio-Rad, Watford, UK). Expression levels of mRNAs of interest were normalized to an internal control mRNA, either *B2M* (beta-2-microglobulin) or *RPL13A* (ribosomal protein L13a), which were stable mRNAs unaffected by the experimental conditions. Each independent real-time PCR was performed in technical triplicate. Experimental design, relative mRNA expression calculations and statistical tests of significance were performed using guidelines described^56^.

### Microarray and GSEA

Following treatment as indicated in the Figure legends, RNA was extracted using TRI reagent as described above. RNA purity and concentration were checked using a Nanodrop 2000 spectrophotometer (Thermo Fisher Scientific, Loughborough, UK) to determine the A260:A280 ratio. RNA was sent to Cambridge Genomic Services (Department of Pathology, University of Cambridge, Cambridge, UK) and further RNA quality control checks performed. Sample was amplified using Ambion Illumina TotalPrep RNA Amplification Kit (Thermo Fisher Scientific, Loughborough, UK), and then labelled, washed and analytical probes bound as part of the Illumina Whole-Genome Gene Expression HumanHT-12 v4 Chip Kit (Illumina, Eindhoven, The Netherlands) according to manufacturer’s instructions. Array scanning was performed using BeadArray Reader (Illumina, Eindhoven, The Netherlands) and features extracted using Illumina BeadStudio software (Illumina, Eindhoven, The Netherlands). Data were provided to us as a.txt file and contained the following information: average signal, *P-*value for bead detection, bead standard error, average number of beads per probe for each sample, probe ID and other annotation information.

Downstream data analysis was performed after quality checks. The data were filtered using the detection *P-*value, at a threshold of > half of the sample to have detected signal for the probe to pass to the comparison stage. The data were then transformed using model-based variance-stabilizing transformation and normalized using quantile normalization, both using the lumi R bioconductor software package. Differentially expressed genes were identified using limma bioconductor software package based on a moderated *t*-test. A linear model was fit through the data using eBayes regression model. To expedite processing and reduce false positives, the data were filtered to remove unannotated probes and unexpressed probes. *P*-values were adjusted for multiple testing using the false detection rate correction method and the adjusted *P*-values were used for all subsequent analysis. The ‘Toptable’ function was used to export lists of top hits with log fold change, adjusted *P*-values, *t*-statistics, *B*-values and *F*-values. GSEA was performed using GSEA software^23^ (Broad Institute, Massachusetts Institute of Technology, USA) and the gene sets indicated in the Figure legends. Data are available in the Gene Expression Omnibus (https://www.ncbi.nlm.nih.gov/geo/) under accession number GSE120993.

### RNA sequencing (RNA-seq)

Following cell treatments as indicated in the Figure legends, total RNA was extracted from snap frozen cell pellets using miRNeasy kit (Qiagen, Manchester, UK) with DNase treatment, following the manufacturer’s instructions. Integrity of the extracted RNA was assessed with the RNA 6000 Nano Assay on the BioAnalyser (Agilent Technologies, Stockport, UK). rRNA (ribosomal ribonucleic acid) was depleted and strand-specific RNA-seq libraries were prepared using the Epicentre strand-specific ScriptSeq RNA-Seq Library Preparation Kit (Illumina, Eindhoven, The Netherlands). Sequencing was performed on the Illumina HiSeq (Illumina, Eindhoven, The Netherlands), three samples per lane, 100 bp paired end reads averaging 100 million reads (50 million pairs) per sample. For gene expression analysis, the count of reads aligning to the gene and ratio per million reads was calculated, and normalized to gene length by reads per kilobase million (RPKM). Parental to resistant differential expression for individual cell lines was measured using DESeq bioconductor software, and cross-sample consistent differential expression significance was confirmed using paired analysis with edgeR bioconductor software. Differentially expressed genes were considered with *q* value < 0.05 (‘high significance’) and additional genes with *P-*value < 0.01 considered if fold change was > 3 or counts per million (CPM) difference > 4 (‘marginal’). TopHat (CCB, Johns Hopkins University, Baltimore, USA) was used to align RNA-seq data, and variants confirmed using Genome Analysis Toolkit (GATK, Broad Institute, Massachusetts Institute of Technology, USA), filtering for variants in coding sequence (CDS) regions (including splice variants), non-synonymous, mean base quality > 30, depth ≥ 5, mutant allele frequency ≥ 0.05 (5%), mean positions in reads ≥ 8. Data are available in the Gene Expression Omnibus (https://www.ncbi.nlm.nih.gov/geo/) under accession number GSE126109.

### Copy number variation analysis

Following cell treatments as indicated in the Figure legends, genomic DNA was prepared using the AllPrep DNA/RNA/miRNA Universal Kit (Qiagen, Manchester, UK). Genomic DNA extracted from experimental (cell lines) and normal control female samples (Promega, Southampton, UK) was labelled using CytoSure HT Genomic DNA Labelling Kit (Oxford Gene Technology, Oxfordshire, UK) following the manufacturer’s instructions for array comparative genomic hybridization analysis. Labelled experimental and control DNA were hybridized to the Agilent Human Genome CGH 2 × 400k Microarray (Agilent Technologies, Stockport, UK). The arrays were incubated for 40 h at 65 °C in a rotating oven at 20 rpm and then washed following the manufacturer’s instructions. Hybridized arrays were scanned on an Agilent G2505C scanner and images analyzed using Agilent Feature Extraction Software (Agilent Technologies, Stockport, UK). Log_2_ ratio values of experiment/control (Cy3/Cy5) were quantified using Nexus Copy Number software (BioDiscovery, El Segundo, California, USA) with the following settings: remove flagged/saturated spots, combine (mean) replicates within array, recenter (median) probes, FASST2 correction, max contiguous probe spacing 1000 kbp, min three probes per segment, robust variance sample QC, remove 3% outliers. Significance threshold of *P* < 0.0005 with high copy number gain > 1.14, gain > 0.42, loss < −0.62, Bis Loss < −1.1 (3:1 sex chromosome gain > 1.2, 4:1 sex chromosome gain > 1.7). Data are available in the Gene Expression Omnibus (https://www.ncbi.nlm.nih.gov/geo/) under accession number GSE126367.

### Statistical analyses

Results, unless otherwise indicated, are presented as mean ± standard deviation (SD) and originate from at least three independent experiments. Statistical significance was determined by two-tailed *t*-test or analysis of variance (ANOVA) using GraphPad Prism 5. Significance values were set at *P* < 0.05 (*), *P* < 0.01 (**) and *P* < 0.001 (***).

## Supplementary information

Supplementary Information

Description of Additional Supplementary Files

Supplementary Data 1

Supplementary Data 2

## Data Availability

All figures summarize raw data that are available upon reasonable request. Microarray (accession number GSE120993), RNA sequencing (GSE126109) and array comparative genomic hybridization (GSE126367) data are available in the Gene Expression Omnibus (https://www.ncbi.nlm.nih.gov/geo/).
