# Supplementary material for: MEK1/2 inhibitor withdrawal reverses acquired resistance driven by BRAFV600E amplification whereas KRASG13D amplification promotes EMT-chemoresistance
Source: Nat Commun. 2019 May 2;10:2030. doi: 10.1038/s41467-019-09438-w (PMC6497655; doi:10.1038/s41467-019-09438-w)
Supplement: Supplementary file 1 — Supplementary Information [file 41467_2019_9438_MOESM1_ESM.pdf]

MEK1/2 inhibitor withdrawal reverses acquired resistance driven by $BRAF^{V600E}$ amplification whereas $KRAS^{G13D}$ amplification promotes EMT-chemoresistance

Sale, M. J. et al.

Supplementary Information

Supplementary Information contains 13 Supplementary Figures, 3 Supplementary Tables and Supplementary References.

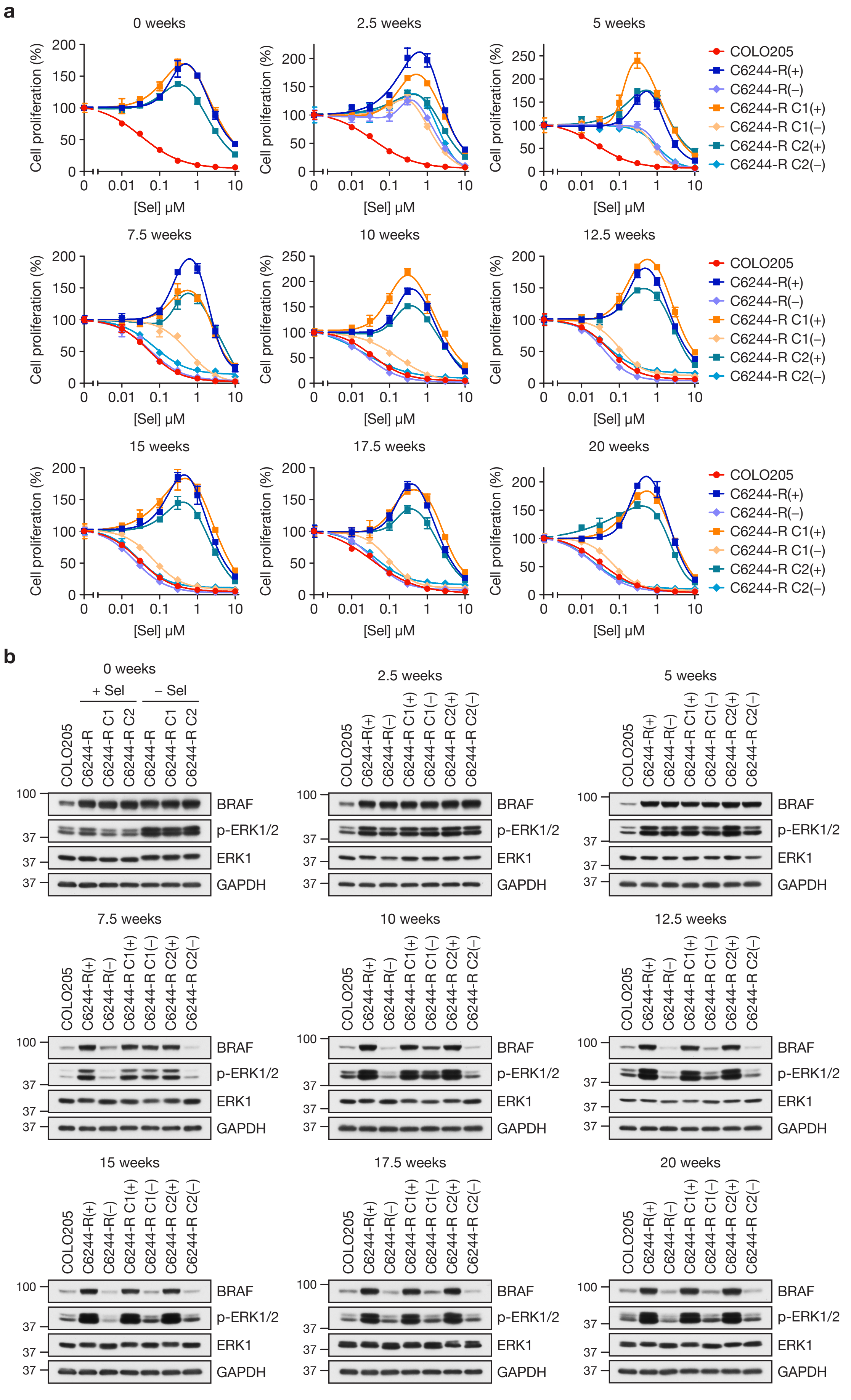

Supplementary Fig. 1 Reversal of acquired resistance and BRAF expression in C6244-R cells is apparent following 2.5 weeks MEKi withdrawal and complete by 12.5 weeks. **a**, **b** Following culture in the presence (+) or absence (COLO205, (–)) of 1 μM selumetinib for the indicated times, cells were treated with increasing concentrations (10 nM to 10 μM) of selumetinib (Sel) for 24 hours, and DNA synthesis assayed by [^{3}H]thymidine incorporation (**a**), or incubated in selumetinib-free medium for 24 hours and lysates western blotted with the indicated antibodies (**b**). C6244-R C1 and C2 are single cell clone derivative cell lines of C6244-R. Results (**a**) are mean ± SD of cell culture triplicates and normalized to control for each cell line.

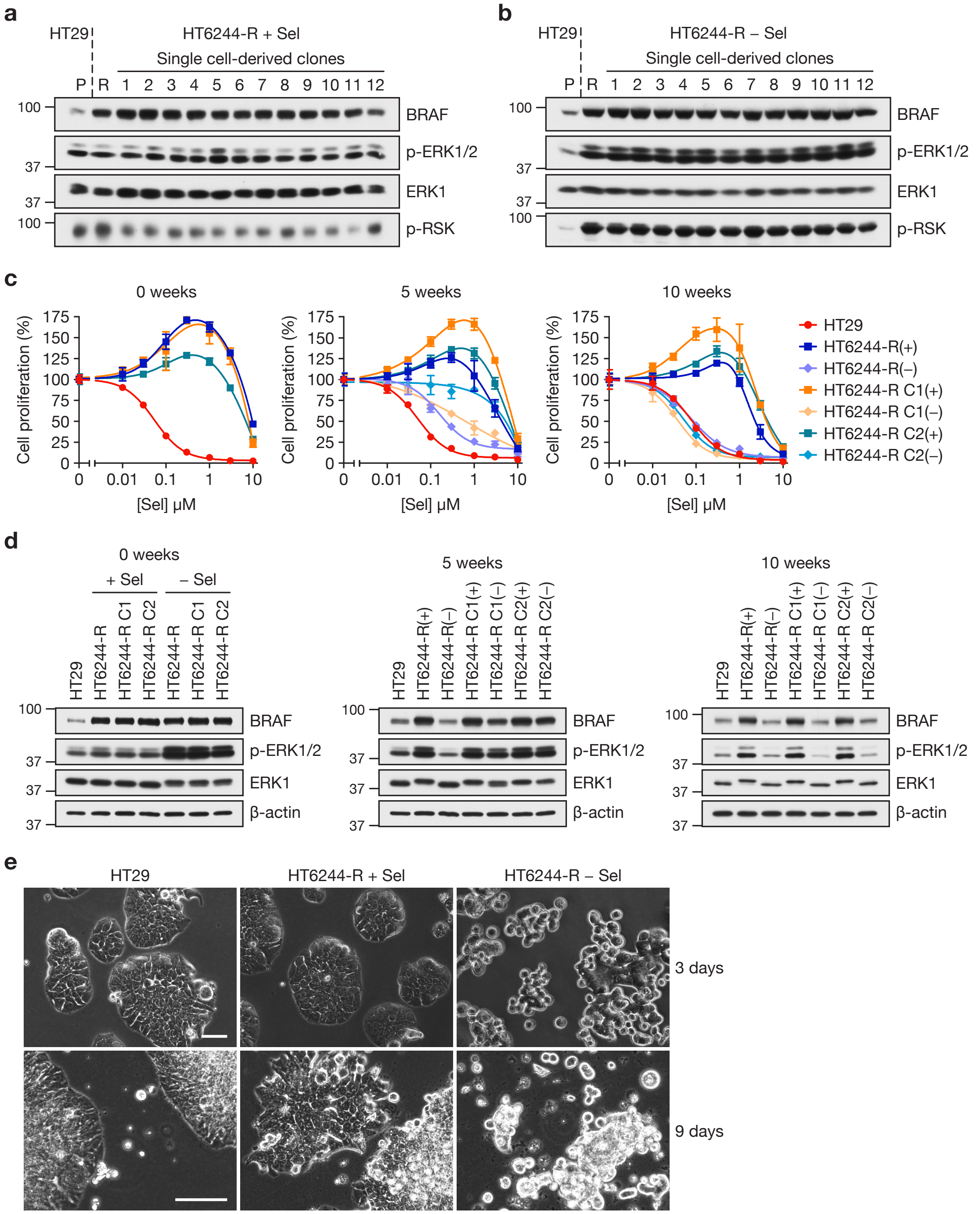

Supplementary Fig. 2 Reversal of acquired resistance and BRAF expression in HT6244-R cells is apparent following 5 weeks selumetinib withdrawal and complete by 10 weeks. **a**, **b** Non-clonal HT6244-R cells (R) were single-cell sorted, expanded and 12 clonal cell lines (1-12) were treated with 1 μM selumetinib (Sel) (**a**) or selumetinib-free medium (**b**) for 24 hours. In parallel parental HT29 cells (P) were treated with selumetinib-free medium for 24 hours. Lysates were western blotted with the indicated antibodies. **c**, **d** Following culture in the presence (+) or absence (HT29, (–)) of 1 μM selumetinib for the indicated times, cells were treated with increasing concentrations (10 nM to 10 μM) of selumetinib (Sel) for 24 hours, and DNA synthesis assayed by [^{3}H]thymidine incorporation (**c**), or incubated in selumetinib-free medium for 24 hours and lysates western blotted with the indicated antibodies (**d**). HT6244-R C1 and C2 are single cell clone derivative cell lines of HT6244-R. Results (**c**) are mean ± SD of cell culture triplicates and normalized to control for each cell line. **e** HT29 and HT6244-R cells were treated with 1 μM selumetinib (HT6244-R + Sel) or DMSO only (HT29, HT6244-R – Sel) for 3 or 9 days and images captured using brightfield phase contrast microscopy. Scale bars indicate 100 μm.

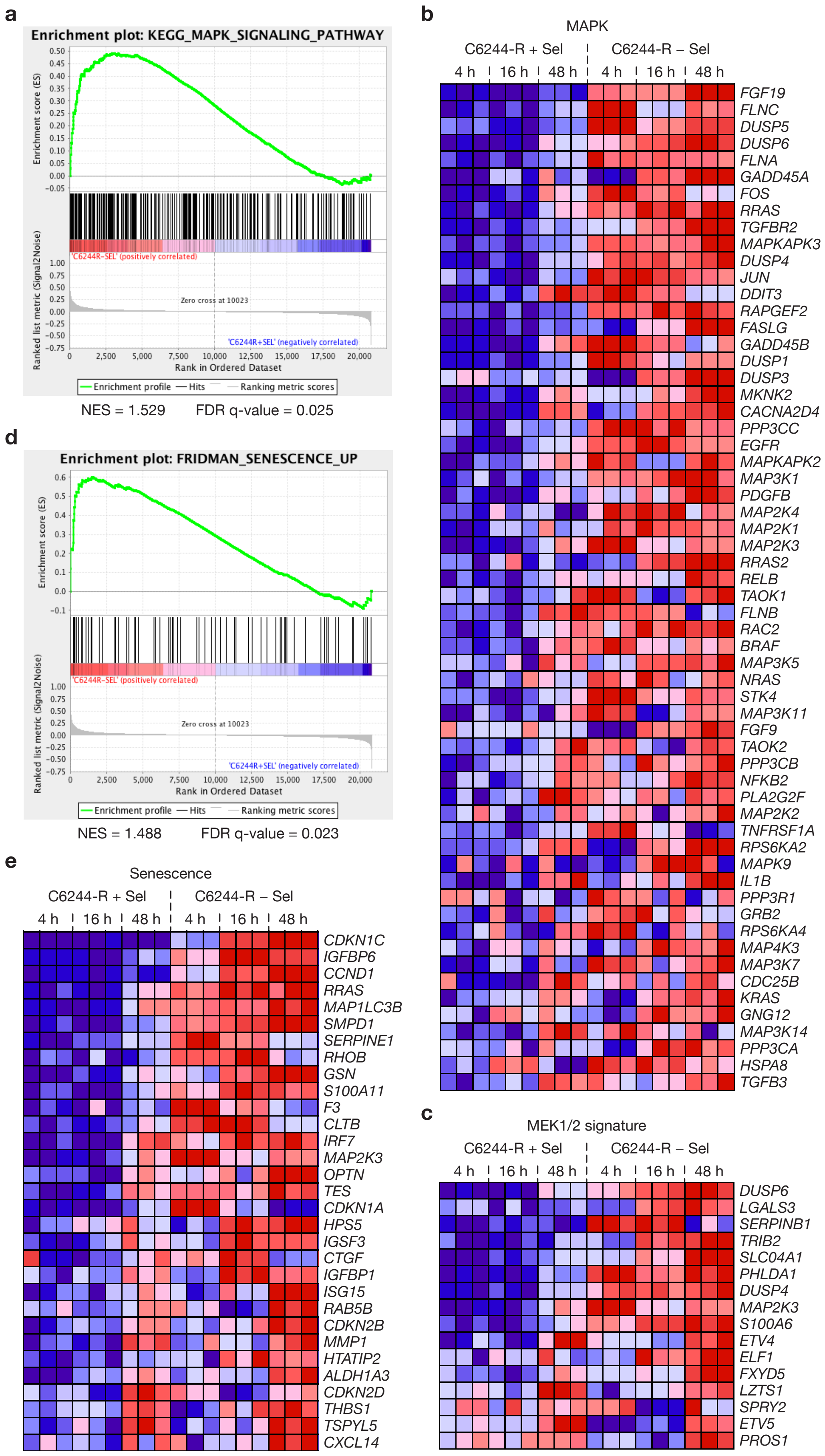

Supplementary Fig. 3 MAPK, MEK1/2 activation signature and senescence gene sets are enriched upon MEKi withdrawal from C6244-R cells. **a**-**e** C6244-R cells were treated with 1 µM selumetinib (+ Sel) or DMSO only (– Sel) for 4, 16 or 48 hours. RNA was extracted and subjected to microarray analysis, from which enrichment of MAPK (curated by Kyoto Encyclopedia of Genes and Genomes (KEGG)) (**a**, **b**), MEK1/2 activation signature[1] (**c**) and senescence[2] (**d**, **e**) gene sets was assessed. Colours (red to blue) represent range of expression values (high to low). NES, normalized enrichment score; FDR, false discovery rate.

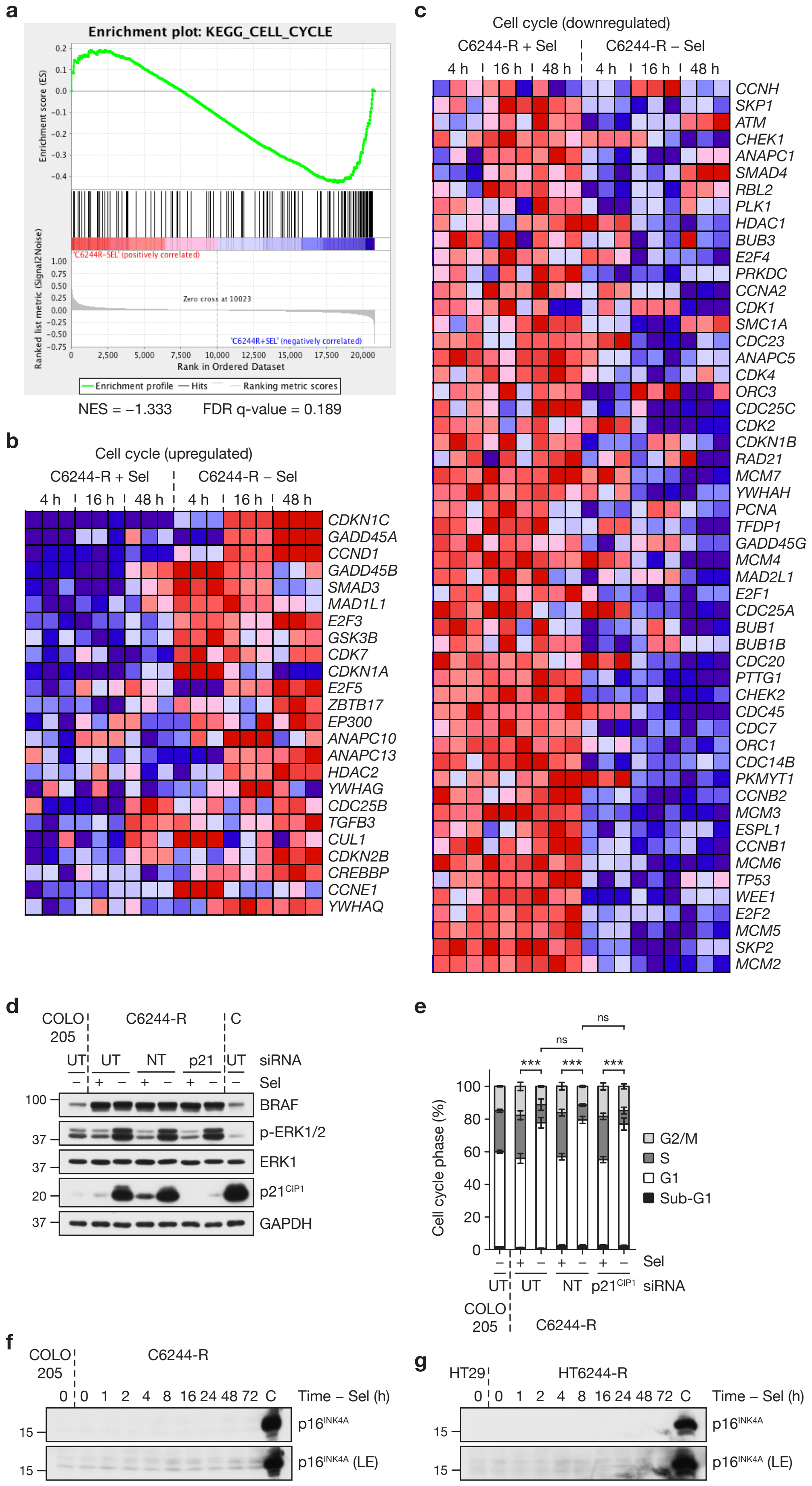
a
Enrichment plot: KEGG_CELL_CYCLE
Enrichment score (ES)
Ranked list metric (Signal2Noise)
'C6244R-SEL' (positively correlated)
Zero cross at 10023
'C6244R+SEL' (negatively correlated)
Rank in Ordered Dataset
Enrichment profile
Hits
Ranking metric scores
NES = −1.333
FDR q-value = 0.189
b
Cell cycle (upregulated)
C6244-R + Sel
C6244-R – Sel
4 h
16 h
48 h
CDKN1C
GADD45A
CCND1
GADD45B
SMAD3
MAD1L1
E2F3
GSK3B
CDK7
CDKN1A
E2F5
ZBTB17
EP300
ANAPC10
ANAPC13
HDAC2
YWHAG
CDC25B
TGFB3
CUL1
CDKN2B
CREBBP
CCNE1
YWHAQ
c
Cell cycle (downregulated)
C6244-R + Sel
C6244-R – Sel
4 h
16 h
48 h
CCNH
SKP1
ATM
CHEK1
ANAPC1
SMAD4
RBL2
PLK1
HDAC1
BUB3
E2F4
PRKDC
CCNA2
CDK1
SMC1A
CDC23
ANAPC5
CDK4
ORC3
CDC25C
CDK2
CDKN1B
RAD21
MCM7
YWHAH
PCNA
TFDP1
GADD45G
MCM4
MAD2L1
E2F1
CDC25A
BUB1
BUB1B
CDC20
PTTG1
CHEK2
CDC45
CDC7
ORC1
CDC14B
PKMYT1
CCNB2
MCM3
ESPL1
CCNB1
MCM6
TP53
WEE1
E2F2
MCM5
SKP2
MCM2
d
COLO 205
C6244-R
C
UT
UT
NT
p21
UT
siRNA
Sel
BRAF
p-ERK1/2
ERK1
p21CIP1
GAPDH
e
Cell cycle phase (%)
ns
***
G2/M
S
G1
Sub-G1
Sel
UT
UT
NT
p21CIP1
siRNA
COLO 205
C6244-R
f
COLO 205
C6244-R
0 0 1 2 4 8 16 24 48 72 C
Time – Sel (h)
p16INK4A
p16INK4A (LE)
g
HT29
HT6244-R
0 0 1 2 4 8 16 24 48 72 C
Time – Sel (h)
p16INK4A
p16INK4A (LE)

Supplementary Fig. 4 Positive cell cycle regulators are typically downregulated following MEKi withdrawal from C6244-R cells while negative regulators are enriched. **a**-**c** C6244-R cells were treated with 1 μM selumetinib (+ Sel) or DMSO only (– Sel) for 4, 16 or 48 hours. RNA was extracted and subjected to microarray analysis, from which enrichment of cell cycle genes (gene set curated by Kyoto Encyclopedia of Genes and Genomes (KEGG)) was evaluated (**a**) and cell cycle genes upregulated (**b**) or downregulated (**c**) plotted. NES, normalized enrichment score; FDR, false discovery rate. **d**, **e** C6244-R cells were left untransfected (UT), transfected with non-targeting (NT) siRNA or transfected with $p21^{CIP1}$-specific siRNA (p21). COLO205 and HCT116 (C) cells were included as untransfected (UT) controls as indicated. 24 hours after transfection cells treated with 1 μM selumetinib (Sel; +) or DMSO only (–) for 4 hours and lysates harvested for western blotting (**d**) or for 48 hours and cell cycle profile assessed by flow cytometry (**e**). $P < 0.001$ (***) or $P > 0.05$ (ns) determined by one-way ANOVA with Tukey's multiple comparisons test of the G1 fractions. **f**, **g** C6244-R (**f**) or HT6244-R (**g**) cells were treated with selumetinib-free media (– Sel) for the indicated time periods (0-72 hours). COLO205 (**f**) or HT29 (**g**) cells were included as time 0 controls, and HeLa cells included as positive control for $p16^{INK4A}$ expression (C). Lysates were western blotted with the indicated antibodies.

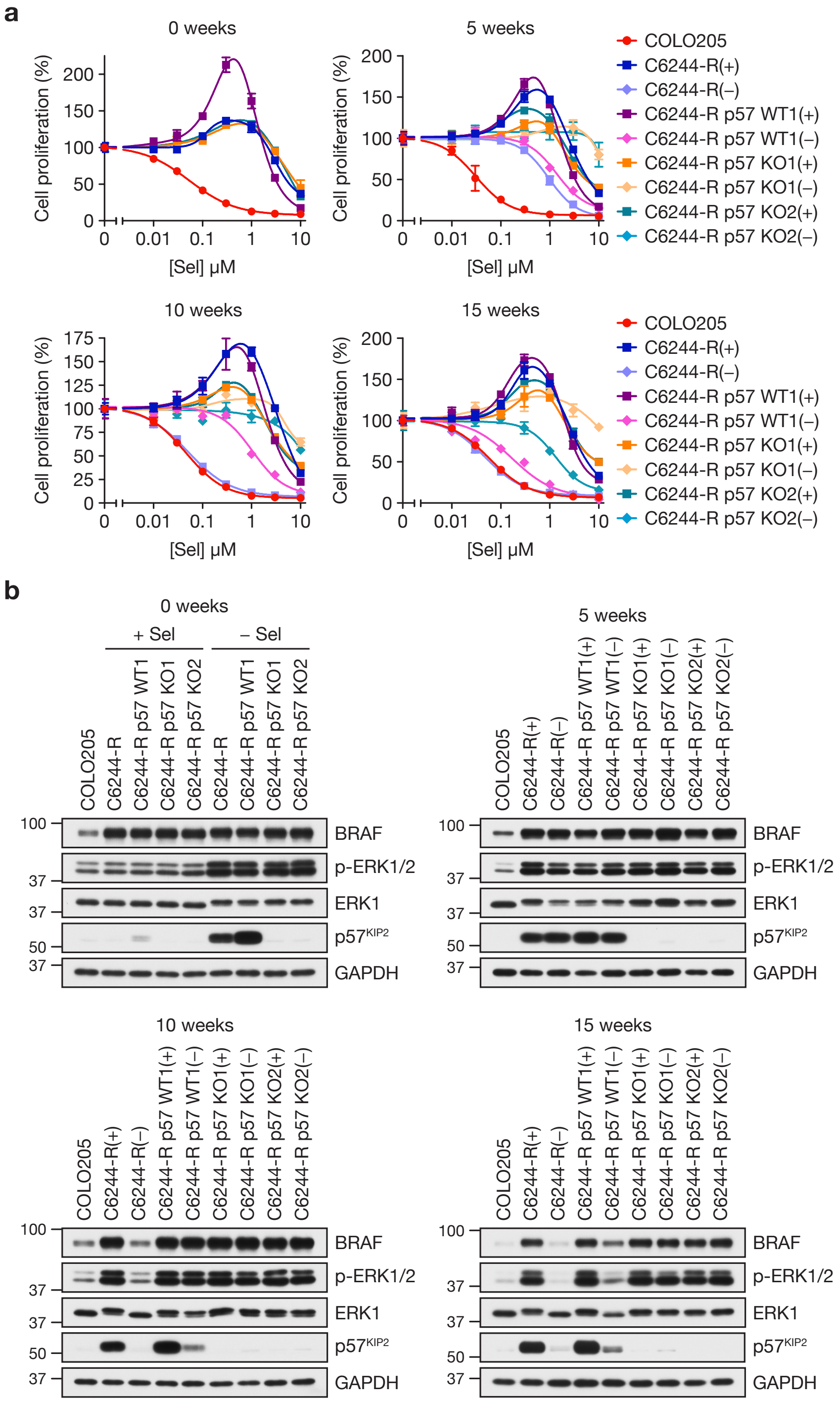

Supplementary Fig. 5 *CDKN1C*/p57^{KIP2} knockout inhibits the reversal of selumetinib resistance in C6244-R cells. **a**, **b** Wild type (p57 WT1) and knockout (p57 KO1, p57 KO2) C6244-R cells generated by CRISPR/Cas9 gene editing with guide RNA (gRNA#2) targeting *CDKN1C* (encoding p57^{KIP2}) were cultured in the presence (+) or absence (COLO205, (–)) of 1 μM selumetinib for the indicated times. Cells were then treated with increasing concentrations (10 nM to 10 μM) of selumetinib (Sel) for 24 hours, and DNA synthesis assayed by [^{3}H]thymidine incorporation (**a**), or incubated in selumetinib-free medium for 24 hours and lysates western blotted with the indicated antibodies (**b**). Results (**a**) are mean ± SD of cell culture triplicates and normalized to control for each cell line.

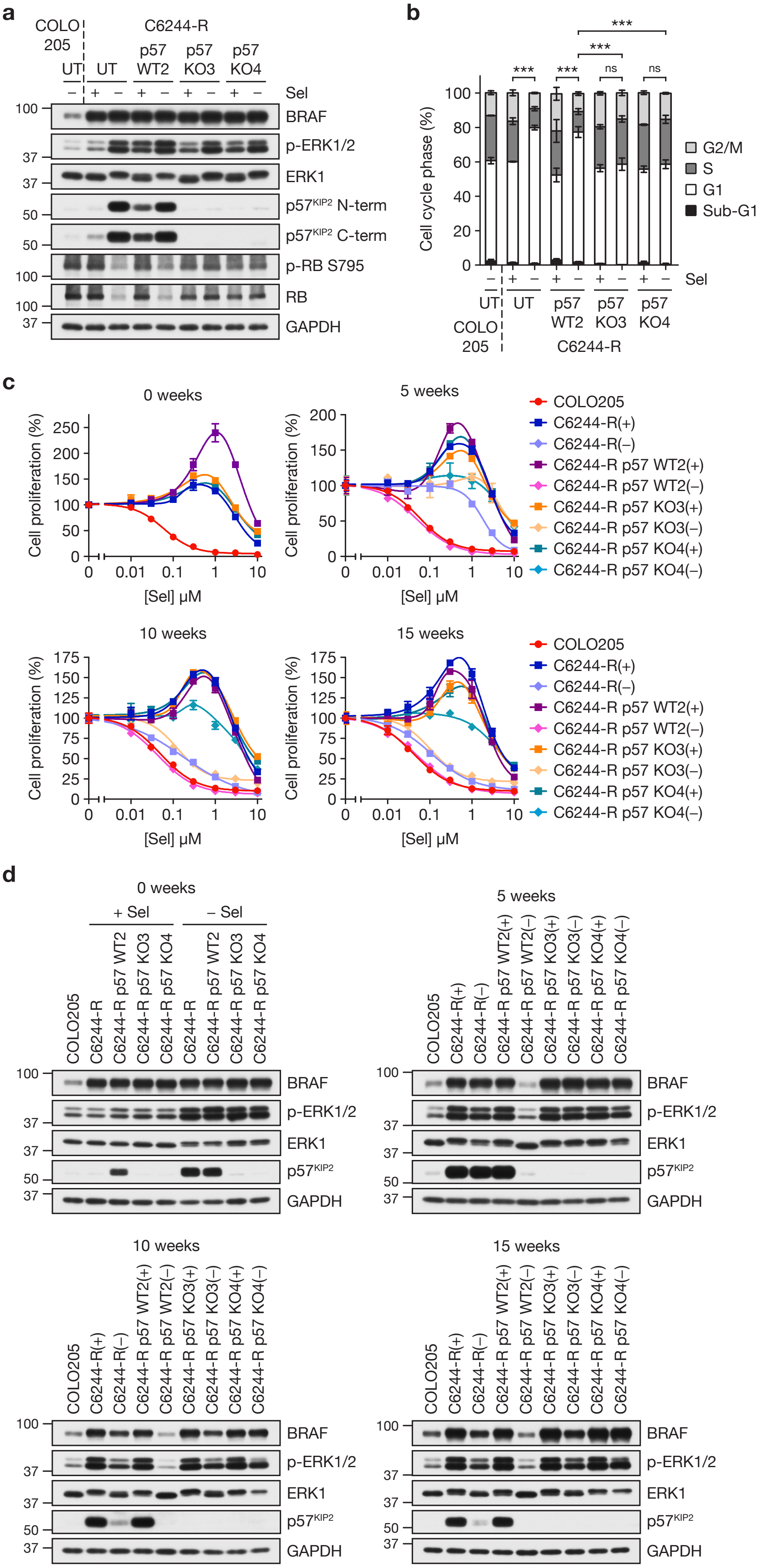
a
COLO 205
C6244-R
UT
UT
p57 WT2
p57 KO3
p57 KO4
Sel
BRAF
p-ERK1/2
ERK1
p57KIP2 N-term
p57KIP2 C-term
p-RB S795
RB
GAPDH
b
Cell cycle phase (%)
G2/M
S
G1
Sub-G1
***
ns
c
0 weeks
5 weeks
10 weeks
15 weeks
Cell proliferation (%)
[Sel] μM
COLO205
C6244-R(+)
C6244-R(–)
C6244-R p57 WT2(+)
C6244-R p57 WT2(–)
C6244-R p57 KO3(+)
C6244-R p57 KO3(–)
C6244-R p57 KO4(+)
C6244-R p57 KO4(–)
d
+ Sel
– Sel
C6244-R p57 WT2
C6244-R p57 KO3
C6244-R p57 KO4
p57KIP2

Supplementary Fig. 6 *CDKN1C*/p57^{KIP2} is required for cell cycle arrest and reversal of resistance upon MEKi withdrawal from C6244-R cells. **a**, **b** Wild type (p57 WT2) and knockout (p57 KO3, p57 KO4) C6244-R cells generated by CRISPR/Cas9 gene editing with guide RNA (gRNA#1) targeting *CDKN1C* (encoding p57^{KIP2}) were treated with (+) selumetinib (Sel) or DMSO only (–) for 72 hours. Untransfected COLO205 and C6244-R cells were included as controls. Cells were western blotted with the indicated antibodies (**a**) or cell cycle profile determined by flow cytometry (**b**). Results (**b**) are mean ± SD of four independent experiments. $P < 0.001$ (***) or $P > 0.05$ (ns) determined by one-way ANOVA with Tukey's multiple comparisons test of the G1 fractions. **c**, **d** Following culture in the presence (+) or absence (–) of 1 μM selumetinib for the indicated times, cells were treated with increasing concentrations (10 nM to 10 μM) of selumetinib (Sel) for 24 hours, and DNA synthesis assayed by [^{3}H]thymidine incorporation (**c**), or incubated in selumetinib-free medium for 24 hours and lysates western blotted with the indicated antibodies (**d**). Results (**c**) are mean ± SD of cell culture triplicates and normalized to control for each cell line.

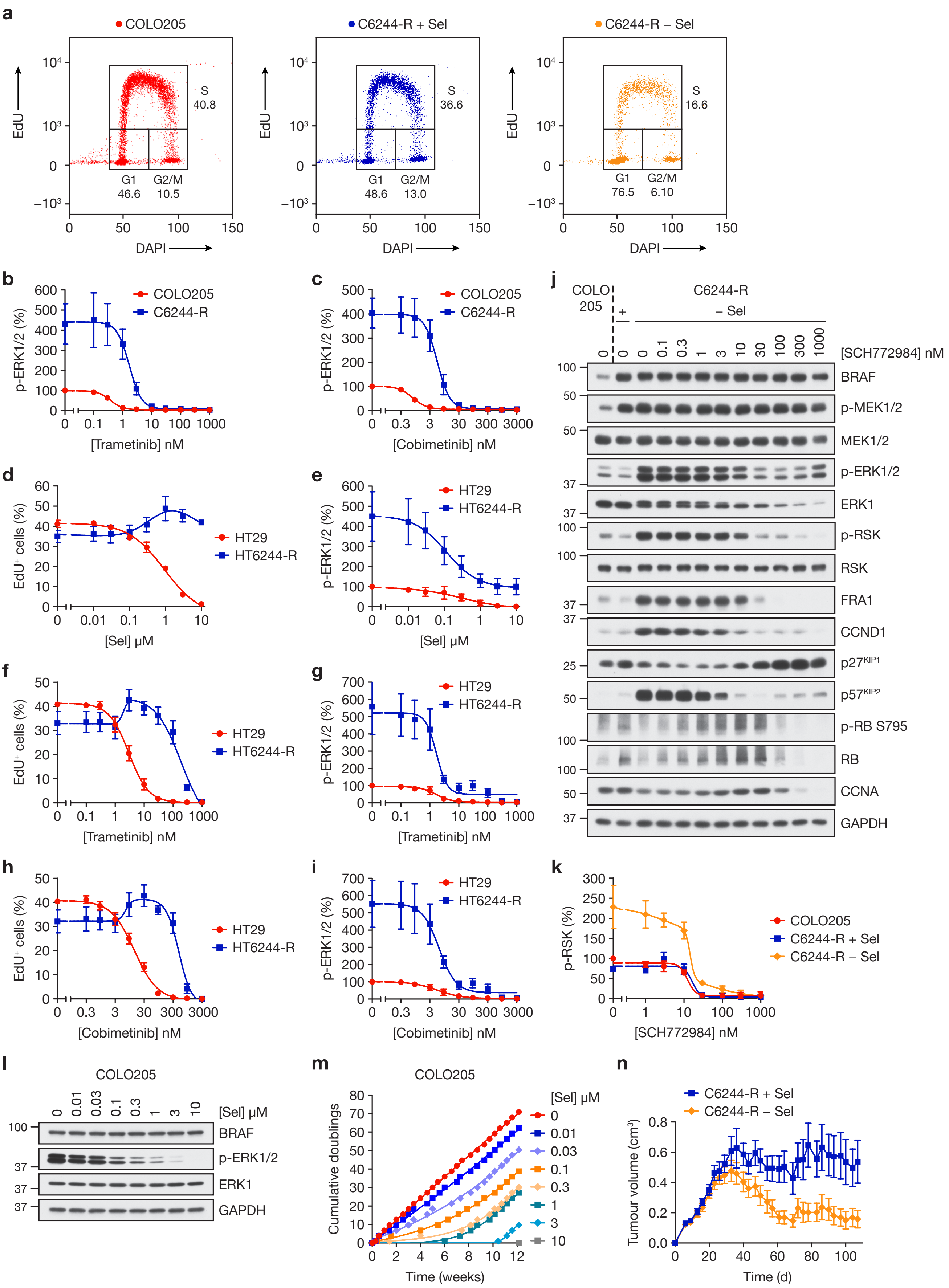
a
COLO205
C6244-R + Sel
C6244-R – Sel
EdU
DAPI
S
40.8
G1
46.6
G2/M
10.5
36.6
48.6
13.0
16.6
76.5
6.10
b
COLO205
C6244-R
p-ERK1/2 (%)
[Trametinib] nM
c
[Cobimetinib] nM
d
HT29
HT6244-R
EdU⁺ cells (%)
[Sel] μM
e
f
g
h
i
j
COLO 205
C6244-R
+
– Sel
[SCH772984] nM
0
0.1
0.3
1
3
10
30
100
300
1000
BRAF
p-MEK1/2
MEK1/2
p-ERK1/2
ERK1
p-RSK
RSK
FRA1
CCND1
p27KIP1
p57KIP2
p-RB S795
RB
CCNA
GAPDH
k
p-RSK (%)
COLO205
C6244-R + Sel
C6244-R – Sel
[SCH772984] nM
l
COLO205
[Sel] μM
0.01
0.03
BRAF
p-ERK1/2
ERK1
GAPDH
m
COLO205
Cumulative doublings
Time (weeks)
[Sel] μM
n
C6244-R + Sel
C6244-R – Sel
Tumour volume (cm³)
Time (d)

Supplementary Fig. 7 C6244-R cells acquire resistance to MEKi by reinstating parental levels of ERK1/2 phosphorylation for normal proliferation in vitro and in vivo. **a** COLO205 and C6244-R cells were treated with 1 μM selumetinib (C6244-R + Sel) or DMSO only (COLO205, C6244-R – Sel) for 72 hours. EdU incorporation and DAPI staining were determined by flow cytometry. Representative plots with values of percent cells gated are shown. **b**, **c** COLO205 and C6244-R cells were treated with the indicated concentrations of trametinib (**b**) or cobimetinib (**c**) for 72 hours. Phospho-ERK1/2 levels were determined by high-content image analysis. Results are normalized to COLO205 control. **d**-**i** HT29 and HT6244-R cells were treated with the indicated concentrations of selumetinib (**d**, **e**), trametinib (**f**, **g**) or cobimetinib (**h**, **i**) for 72 hours. Percent EdU-positive (EdU+) cells (**d**, **f**, **h**) or phospho-ERK1/2 levels (**e**, **g**, **i**) were determined by high-content image analysis. Results are normalized to HT29 control. **j** C6244-R cells were treated with the indicated concentrations of SCH772984 (SCH) in the absence of selumetinib (– Sel) for 72 hours. COLO205, and C6244-R cells treated with 1 μM selumetinib (+) were included as controls. Lysates were western blotted with the indicated antibodies. **k** COLO205 and C6244-R cells were treated as indicated with SCH772984 for 72 hours, either in the presence (C6244-R + Sel) or absence (COLO205, C6244-R – Sel) of 1 μM selumetinib. Phospho-RSK levels were determined by high-content image analysis. Results were normalized to COLO205 control. **l** COLO205 cells were treated with the indicated concentrations of selumetinib (Sel) for 24 hours. Lysates were western blotted with the indicated antibodies. **m** Cumulative doubling tallies for COLO205 cells growing in the presence of the indicated concentrations of selumetinib over 12 weeks. **n** Mice were dosed orally with vehicle only (C6244-R – Sel) or 10 mg kg-1 selumetinib (C6244-R + Sel) twice daily. The following day, C6244-R cells were injected subcutaneously in to 21 mice per treatment group. Mice continued to be dosed twice daily and tumour growth was monitored twice weekly. Results are mean ± SEM. **b**-**i**, **k** Results are mean ± SD of at least three independent experiments.

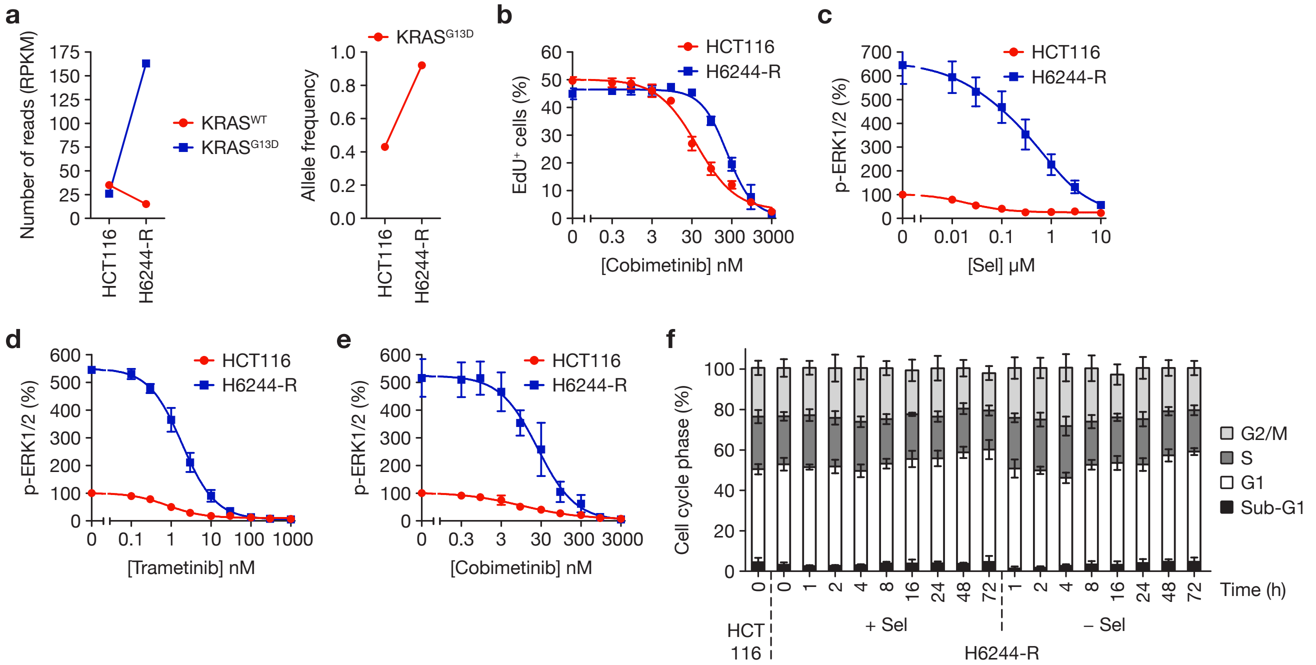

Supplementary Fig. 8 MEKi withdrawal from H6244-R cells with $KRAS^{G13D}$ amplification has no effect on cell proliferation. **a** HCT116 and H6244-R cells were treated with DMSO only (HCT116) or 2 μM selumetinib (H6244-R). RNA was then extracted and subjected to RNA sequencing analysis. The number of reads per kilobase million (RPKM) and allele frequency are shown. **b**-**e** HCT116 and H6244-R cells were treated with the indicated concentrations of cobimetinib (**b**, **e**), selumetinib (Sel) (**c**) or trametinib (**d**) for 72 hours. Percent EdU-positive (EdU^+) cells (**b**) or p-ERK1/2 levels (**c**-**e**) were determined by high-content image analysis. Results are mean ± SD of three independent experiments and normalized to HCT116 control. **f** HCT116 and H6244-R cells were treated with either 2 μM selumetinib (H6244-R + Sel) or DMSO only (HCT116, H6244-R – Sel) for the indicated times. Cell cycle distribution was determined by flow cytometry. Results are mean ± SD of three independent experiments.

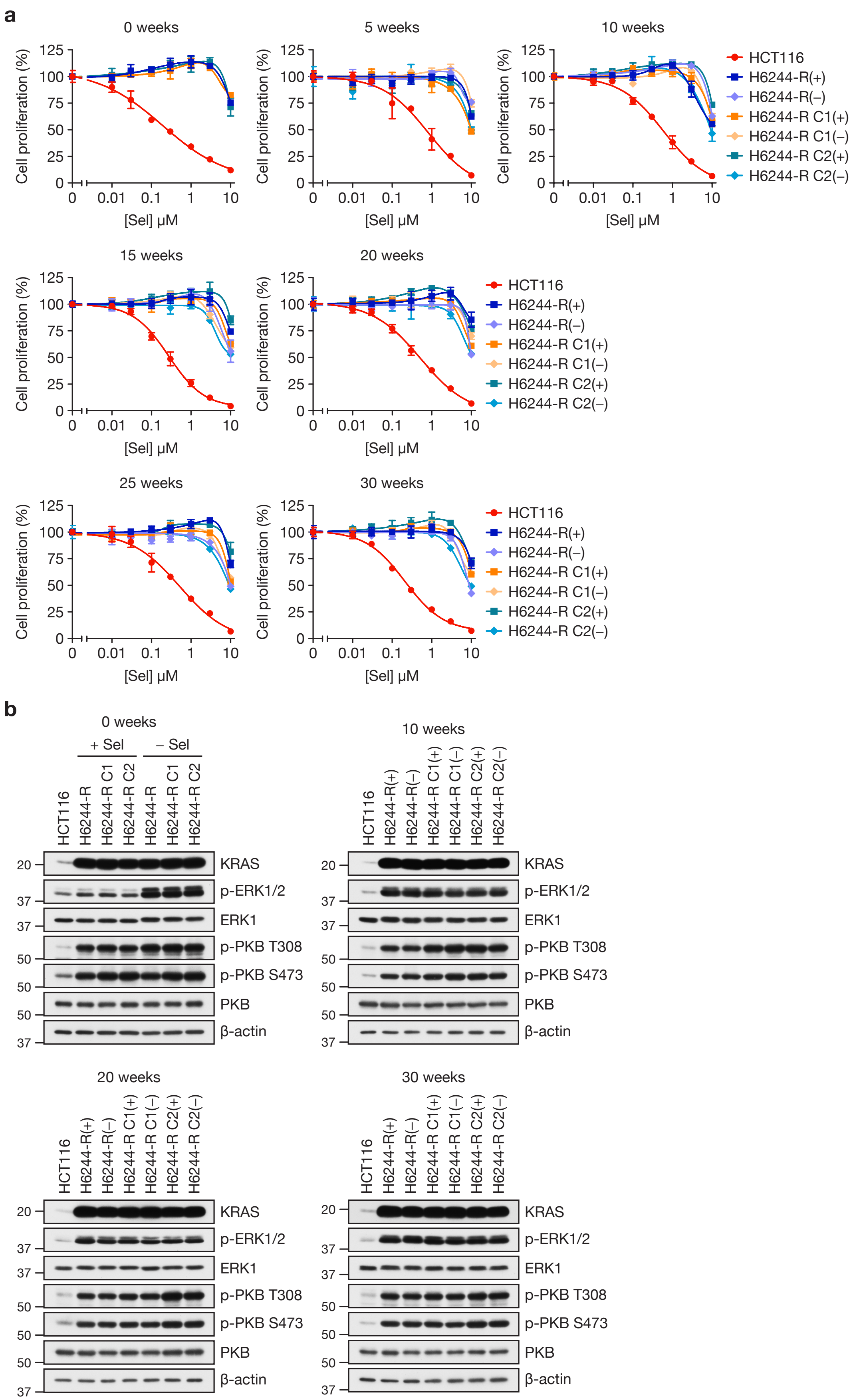

Supplementary Fig. 9 Selumetinib resistance is not reversible in KRAS^{G13D} amplified H6244-R cells. **a**, **b** Following culture in the presence (+) or absence (HCT116, (–)) of 2 µM selumetinib for the indicated times, cells were treated with increasing concentrations (10 nM to 10 µM) of selumetinib (Sel) for 24 hours, and DNA synthesis assayed by [^{3}H]thymidine incorporation (**a**), or incubated in selumetinib-free medium for 24 hours and lysates western blotted with the indicated antibodies (**b**). Results (**a**) are mean ± SD of cell culture triplicates.

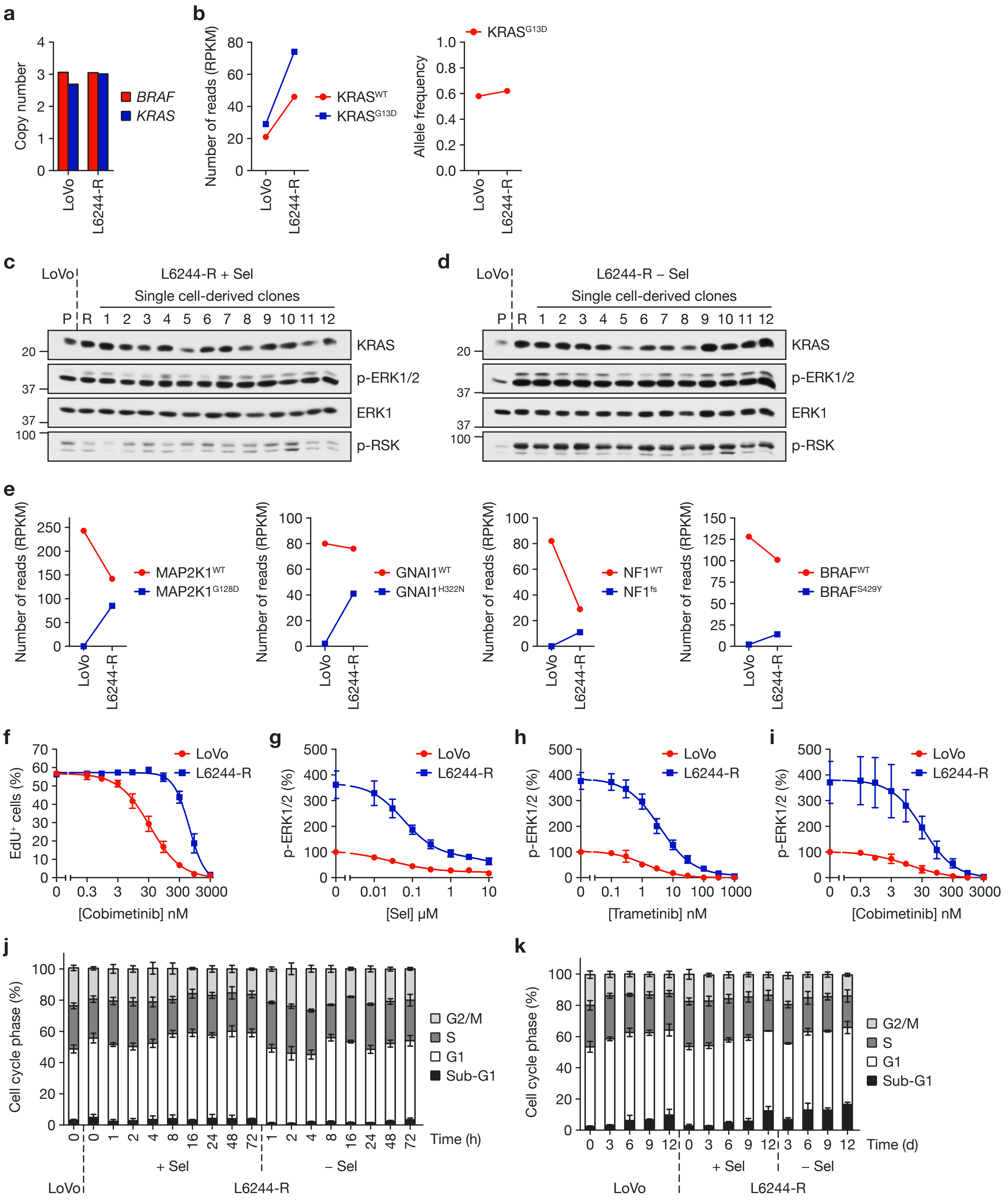

Supplementary Fig. 10 MEKi withdrawal from L6244-R cells has no effect on cell proliferation. **a** LoVo and L6244-R cells were treated with DMSO only (LoVo) or 4 μM selumetinib (L6244-R). DNA was extracted and copy number assessed by array CGH. **b** LoVo and L6244-R cells were treated with DMSO only (LoVo) or 4 μM selumetinib (L6244-R). RNA was then extracted, subjected to RNA sequencing analysis and the number of reads per kilobase million (RPKM) and allele frequency is shown. **c**, **d** Non-clonal L6244-R cells (R) and 12 single cell clone derivative cell lines (1-12) were treated with 4 μM selumetinib (**c**) or selumetinib-free medium (**d**) for 24 hours. In parallel parental LoVo cells (P) were treated with selumetinib-free medium for 24 hours for comparison. Lysates were fractionated by SDS-PAGE and immunoblotted with the indicated antibodies. **e** RNA sequencing analysis on LoVo and L6244-R cells as described in (**b**) and the number of reads per kilobase million (RPKM) is shown. **f**-**i** LoVo and L6244-R cells were treated with the indicated concentrations of cobimetinib (**f**, **i**), selumetinib (Sel) (**g**) or trametinib (**h**) for 72 hours. Percent EdU-positive (EdU+) cells (**f**) or phospho-ERK1/2 levels (**g**-**i**) were determined by high-content image analysis. Results are mean ± SD of three independent experiments and normalized to LoVo control. **j**, **k** LoVo and L6244-R cells were treated with 4 μM selumetinib (L6244-R + Sel) or DMSO only (LoVo, L6244-R – Sel) for the indicated times. Cell cycle distribution was determined by flow cytometry. Results are mean ± SD of three independent experiments.

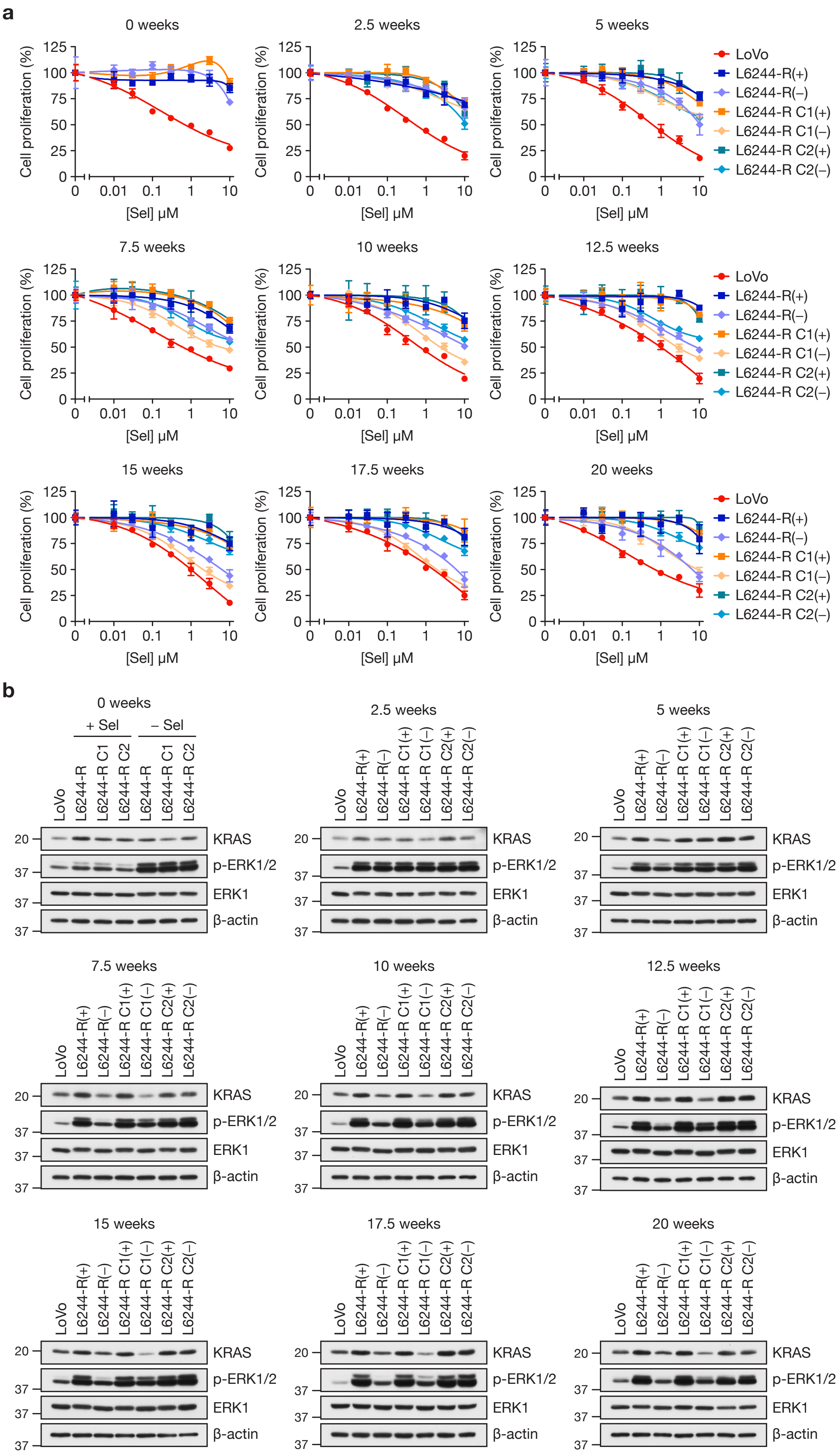

Supplementary Fig. 11 MEKi resistance is partially reversible in L6244-R cells. **a**, **b** Following culture in the presence (+) or absence (LoVo, (–)) of 4 μM selumetinib for the indicated times, cells were treated with increasing concentrations (10 nM to 10 μM) of selumetinib (Sel) for 24 hours, and DNA synthesis assayed by [^{3}H]thymidine incorporation (**a**), or incubated in selumetinib-free medium for 24 hours and lysates western blotted with the indicated antibodies (**b**). Results (**a**) are mean ± SD of cell culture triplicates and normalized to control for each cell line.

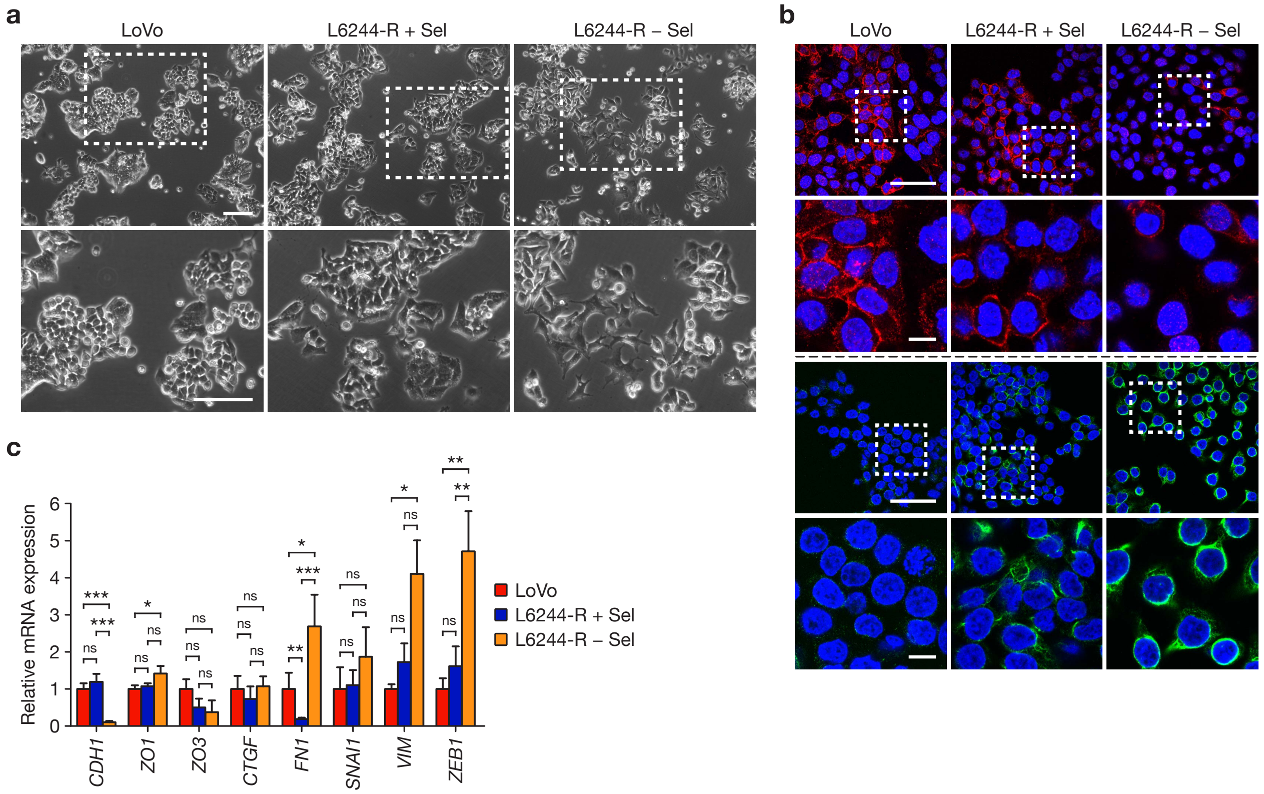

Supplementary Fig. 12 MEKi withdrawal from L6244-R cells induces EMT. **a** LoVo and L6244-R cells were treated with 4 μM selumetinib (L6244-R + Sel) or DMSO only (LoVo, L6244-R – Sel) for 9 days and imaged by brightfield phase contrast microscopy. Scale bars indicate 100 μm. **b** LoVo and L6244-R cells were treated with 4 μM selumetinib (L6244-R + Sel) or DMSO only (LoVo, L6244-R – Sel) for 9 days and stained for CDH1 (red) or VIM (green) and nuclei (blue). Scale bars indicate 50 μm (upper panels) and 10 μm (lower panels). **c** LoVo and L6244-R cells were treated with 4 μM selumetinib (L6244-R + Sel) or DMSO only (LoVo, L6244-R – Sel) for 9 days. Cells were lysed, RNA extracted and the relative expression of the indicated mRNAs determined by RT-qPCR and normalization to *RPL13A* expression. Results are mean ± SD of at least three independent experiments each performed in technical triplicate. $P < 0.001$ (***), $P < 0.01$ (**), $P < 0.05$ (*), $P > 0.05$ (ns) as determined by one-way ANOVA with Tukey's multiple comparisons test.

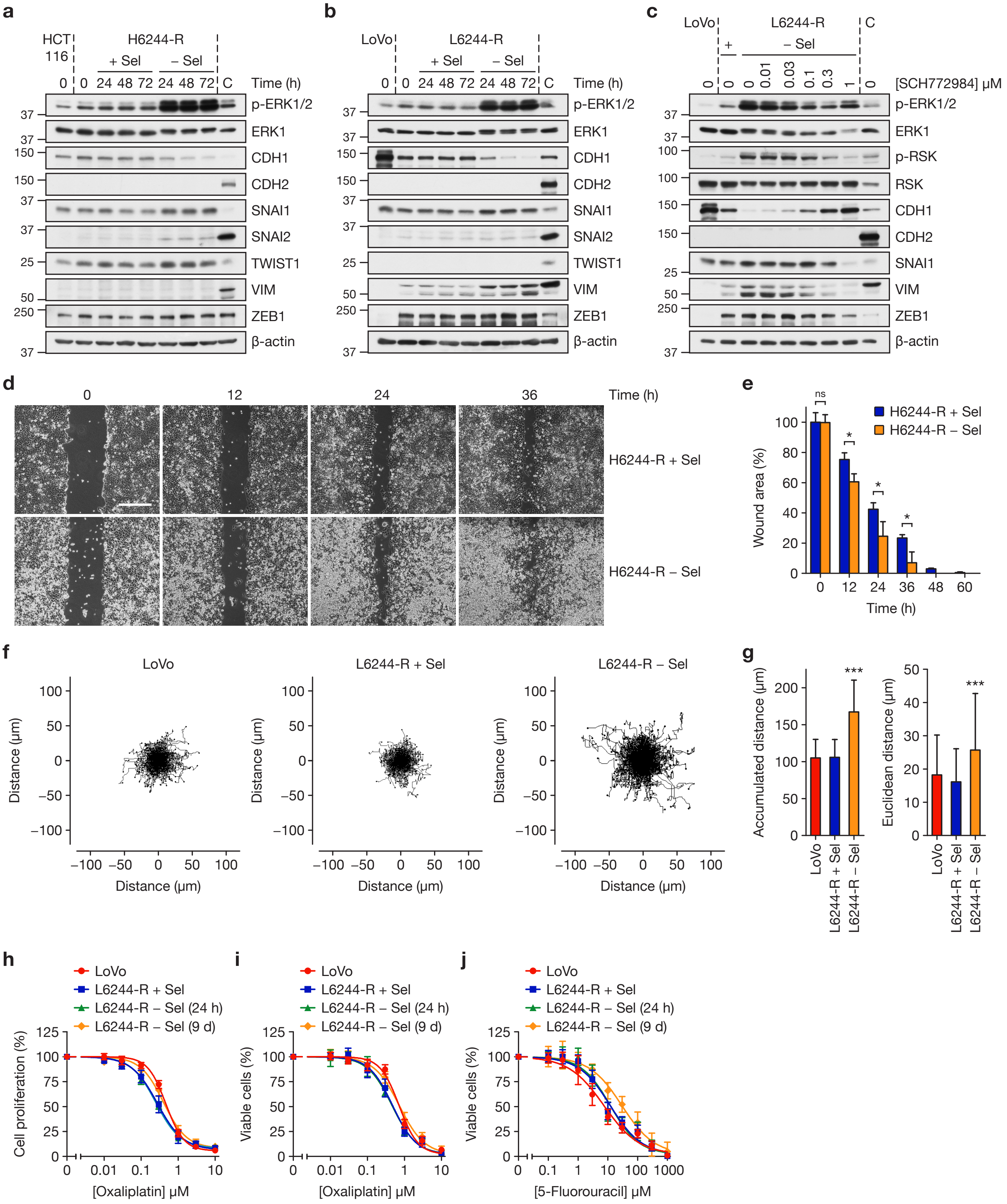
a
HCT 116
H6244-R
+ Sel
– Sel
0 0 24 48 72 24 48 72 C
Time (h)
p-ERK1/2
ERK1
CDH1
CDH2
SNAI1
SNAI2
TWIST1
VIM
ZEB1
β-actin
b
LoVo
L6244-R
+ Sel
– Sel
0 0 24 48 72 24 48 72 C
Time (h)
c
LoVo
L6244-R
C
+
– Sel
0 0 0 0.01 0.03 0.1 0.3 1 0
[SCH772984] μM
p-ERK1/2
ERK1
p-RSK
RSK
CDH1
CDH2
SNAI1
VIM
ZEB1
β-actin
d
0
12
24
36
Time (h)
H6244-R + Sel
H6244-R – Sel
e
H6244-R + Sel
H6244-R – Sel
Wound area (%)
Time (h)
ns
f
LoVo
L6244-R + Sel
L6244-R – Sel
Distance (μm)
Distance (μm)
g
Accumulated distance (μm)
Euclidean distance (μm)
LoVo
L6244-R + Sel
L6244-R – Sel
***
h
LoVo
L6244-R + Sel
L6244-R – Sel (24 h)
L6244-R – Sel (9 d)
Cell proliferation (%)
[Oxaliplatin] μM
i
Viable cells (%)
[Oxaliplatin] μM
j
Viable cells (%)
[5-Fluorouracil] μM

Supplementary Fig. 13 EMT induced by MEKi withdrawal is ERK1/2-dependent and associated with enhanced cell motility and chemoresistance. **a** HCT116 and H6244-R cells were treated with 2 μM selumetinib (+ Sel) or DMSO only (HCT116, – Sel) for the indicated times. **b** LoVo and L6244-R cells were treated with 4 μM selumetinib (+ Sel) or DMSO only (LoVo, – Sel) for the indicated times. **c** L6244-R cells were treated with the indicated concentrations of SCH772984 in the absence of selumetinib (– Sel) for 72 hours. LoVo, and L6244-R cells treated with 4 μM selumetinib (+), were included as controls. **a**-**c** Lysates were western blotted with the indicated antibodies, and A549 cells were used for positive control (C), except for SNAI2 (SW620 cells). Results are representative of at least two experiments giving equivalent results. **d**, **e** H6244-R cells were treated with 2 μM selumetinib (H6244-R + Sel) or DMSO only (HCT116, H6244-R – Sel) for 9 days. Confluent cell monolayers were scratched and wound healing monitored at the indicated times. Representative results (**d**) are shown with mean ± SD of three independent experiments (**e**). $P < 0.05$ (*) or $P > 0.05$ (ns) determined by unpaired two-tailed *t*-test. Scale bar indicates 1 mm. **f**, **g** LoVo and L6244-R cells were treated with 4 μM selumetinib (L6244-R + Sel) or DMSO only (LoVo, L6244-R – Sel) for 9 days. Cell movements were then tracked every 10 minutes for the following 16 hours. Spider diagrams of individual cell paths (**f**) are shown with mean accumulated and Euclidean distance ± SD (n > 300 cells for each cell line) (**g**). $P < 0.001$ (***) determined by one-way ANOVA with Tukey's multiple comparisons test. **h**-**j** LoVo and L6244-R cells were treated with 4 μM selumetinib (L6244-R + Sel) or DMSO only (LoVo, L6244-R – Sel (9 d)) for 9 days. The following day cells were treated with the indicated concentrations of oxaliplatin for 24 (**h**) or 72 (**i**) hours, or 5-fluorouracil for 72 hours (**j**), maintaining prior presence or absence of selumetinib. As a control, L6244-R cells maintained in selumetinib throughout were switched to selumetinib free-medium for the final 24 hours of treatment (L6244-R – Sel (24 h)). Cell proliferation was assayed by [^{3}H]thymidine incorporation (**h**) or cell viability assayed using Sytox Green (**i**, **j**). Results are mean ± SD of three independent experiments and normalized to control for each cell line.

Supplementary Table 1. Colorectal cancer consensus genes mutated in COLO205, HCT116, HT29 and LoVo cells.

	COLO205		HT29		HCT116		LoVo	
Gene	COSMIC	CCLE	COSMIC	CCLE	COSMIC	CCLE	COSMIC	CCLE
APC	T1556fs*3	E1554fs	E853*; T1556fs*3	E853*; E1554fs	WT	WT	R1114*; M1431fs; R2816Q	R1114*; T1430fs; R2816Q
TP53	Y103_R110del†	WT†	R273H	R273H	WT	WT	WT	WT
KRAS	WT	WT	WT	WT	G13D	G13D	G13D	G13D
FAT4	K4532N	K4532N	WT	WT	L1374P	L1374P; G2644fs	A4629T	A4629T
LRP1B	WT	WT	WT	WT	A3541V	A3541V	G485fs; I2362V; I3155T	G485fs; I2362V; I3155T
PIK3CA	WT	WT	P449T	P449T	H1047R	H1047R	WT	WT
TGFBR2	WT	WT	WT	WT	WT	WT	WT	WT
BRAF	V600E	V600E	T119S; V600E	T119S; V600E	WT	WT	WT	WT
ACVR2A	WT	WT	WT	WT	WT	K437fs	WT	K437fs
KMT2C	WT	WT	P2415S	P2415S	Q419R; I1344fs*1	Q419R; I1344fs; K2797fs	C1013R; E1313K	C1013R; E1313K; K2797fs
ZFHX3	WT	WT	I1217M	I1217M	G2941R	G2941R	WT	WT
KMT2D	WT	WT	WT	WT	R2173fs*6	V160M; P2442fs	H77fs; P648fs	H77fs; P648fs
FBXW7	WT	WT	WT	WT	WT	WT	R505C	R505C
RNF43	WT	WT	WT	WT	R117fs*41	R117fs	WT	WT
ARID1A	WT	WT	WT	WT	WT	WT	F2141fs*59	P1923fs
SMAD4	WT	WT	Q311*	Q311*	WT	WT	WT	WT
TRRAP	WT	WT	WT	WT	H3023Y; T3634A	H3023Y; T3663A	WT	WT
FAT1	WT	WT	WT	WT	WT	I247T	WT	WT
PREX2	WT	WT	WT	WT	R363W; S565fs*10	R363W; R562fs	Y176H; C938F	Y176H; C938F
TCF7L2	WT	WT	WT	WT	WT	WT	WT	R482fs
NF1	WT	WT	WT	WT	P388T; P678fs*10; I679fs*21	P388T; T676fs;	R1695Q	R1695Q
CDKN2A	WT	WT	WT	WT	WT‡	G23fs‡; E33fs	WT	WT
CTNNB1	WT	WT	WT	WT	S45del	S45del	WT	R535Q
MLH1	WT	WT	WT	WT	S252*	S252*	WT	WT
PTEN	WT	WT	WT	WT	WT	WT	WT	WT
RB1	WT	WT	WT	WT	WT	WT	WT	WT
**MSS/MSI**	MSS		MSS		MSI		MSI	
**CIMP**	CIMP+		CIMP+		CIMP+		CIMP−	

* Introduces stop codon.

† Mutation in COLO205 *TP53* at Y103 further supported by Ikediobi et al.[3], Berg et al.[4] and NCI CellMiner portal (http://discover.nci.nih.gov/cellminerdata/rawdata/mutation.txt).

‡ Mutation in HCT116 *CDKN2A* supported by Ikediobi et al.[3], Okamoto et al.[5] and NCI CellMiner portal.

CCLE, Cancer Cell Line Encyclopedia (https://portals.broadinstitute.org/ccle); CIMP, CpG island methylator phenotype; COSMIC, Catalogue of Somatic Mutations in Cancer (https://cancer.sanger.ac.uk/cosmic); del, deletion; fs, frameshift; MSI, microsatellite instability; MSS, microsatellite stability; WT, wild type. Predicted zygosity: heterozygous (blue); homozygous (red). Includes top 20 large intestine cancer consensus genes in COSMIC. CIMP and microsatellite status as in reference Berg et al.[4].

Supplementary Table 2. MEKi withdrawal from H6244-R cells does not increase tumour invasiveness *in vivo*.

HCT116		H6244-R + Sel		H6244-R – Sel	
1	Tumour invades fat	6	Tumour invades fat & muscle	27	
2		7	Tumour invades fat	28	Tumour invades fat
3		8		29	Tumour invades fat & muscle
4	Tumour invades fat	9	Tumour invades fat & muscle	30	
5		10		31	Tumour invades fat & muscle
		11		32	
		12	Tumour invades fat & muscle	33	
		13	Tumour invades fat & muscle	34	
		14		35	Tumour invades fat
		15		36	
		16		37	
		17	Tumour invades fat & muscle	38	
		18		39	
		19	Tumour invades fat & muscle	40	Tumour invades fat
		20		41	
		21		42	Tumour invades fat
		22		43	Tumour invades fat & muscle
		23		44	
		24		45	
		25	Tumour invades fat & muscle	46	
		26		47	Tumour invades fat & muscle
				48	
**Total invasive**	**2/5 (40%)**		**8/21 (38.1%)**		**8/22 (36.4%)**

Supplementary Table 3. Full list of reagents and resources used in this study.

REAGENT or RESOURCE		SOURCE	IDENTIFIER
**Antibodies**			
Anti-mouse Alexa Fluor 488 antibody	ICC 1:300	Thermo Fisher Scientific	Cat#A-11001
Anti-rabbit Alexa Fluor 488 antibody	ICC 1:300	Thermo Fisher Scientific	Cat#A-11008
Anti-rabbit Alexa Fluor 568 antibody	ICC 1:300	Thermo Fisher Scientific	Cat#A-11011
β-actin; mouse monoclonal (clone AC-15)	WB 1:2000	Sigma-Aldrich	Cat#A5441
BID; rabbit polyclonal	WB 1:1000	Cell Signaling Technology	Cat#2002
BIM; rabbit polyclonal	WB 1:1000	Merck Millipore	Cat#AB17003
BRAF; mouse monoclonal (clone F-7)	WB 1:500	Santa Cruz Biotechnology	Cat#sc-5284;
BrdU; mouse monoclonal (clone Bu20a)	FC 1:200	Cell Signaling Technology	Cat#5292
CCNA (Cyclin A); rabbit polyclonal	WB 1:500	Santa Cruz Biotechnology	Cat#sc-751
CCND1 (Cyclin D1); mouse monoclonal (clone DCS-6)	WB 1:200	Merck Millipore	Cat#CC12
CDH1 (E-cadherin); rabbit monoclonal (clone 24E10)	WB 1:1000; ICC 1:200	Cell Signaling Technology	Cat#3195
CDH2 (N-Cadherin); rabbit polyclonal	WB 1:1000	Cell Signaling Technology	Cat#4061
CDK2; rabbit polyclonal	WB 1:500	Santa Cruz Biotechnology	Cat#sc-163
CDK2; mouse monoclonal agarose conjugate (clone AN21.2)	WB 1:500	Santa Cruz Biotechnology	Cat#sc-53219 AC
ERK1; mouse monoclonal (clone MK12)	WB 1:1000	BD Biosciences	Cat#610031
FRA1; rabbit polyclonal	WB 1:500	Santa Cruz Biotechnology	Cat#sc-183
GAPDH; rabbit monoclonal (clone EPR16891)	WB 1:2000	Abcam	Cat#ab181602
KRAS; mouse monoclonal (clone 234-4.2)	WB 1:200	Merck Millipore	Cat#OP24
LC3B; rabbit polyclonal	WB 1:500	Sigma-Aldrich	Cat#L7543
MEK1/2; rabbit polyclonal	WB 1:1000	Cell Signaling Technology	Cat#9122
NOXA; mouse monoclonal (clone 114C307)	WB 1:200	Merck Millipore	Cat#OP180
p15^{INK4B}; rabbit polyclonal	WB 1:500	Santa Cruz Biotechnology	Cat#sc-613
p16^{INK4A}; rabbit polyclonal	WB 1:500	Santa Cruz Biotechnology	Cat#sc-759
p18^{INK4C}; mouse monoclonal (clone DCS118)	WB 1:1000	Cell Signaling Technology	Cat#2896
p19^{INKD}; mouse monoclonal (clone DCS-100)	WB 1:500	Santa Cruz Biotechnology	Cat#sc-56334
p21^{CIP1}; mouse monoclonal (clone SXM30)	WB 1:200	BD Biosciences	Cat#556431
p27^{KIP1}; mouse monoclonal (clone DCS72)	WB 1:200	Merck Millipore	Cat#NA35
p53; mouse monoclonal (clone DO-1)	WB 1:500	Merck Millipore	Cat#OP43
p57^{KIP2} (N-term); rabbit monoclonal (clone EP2515Y)	WB 1:500	Abcam	Cat#ab75974
p57^{KIP2} (C-term); rabbit polyclonal	WB 1:1000	Cell Signaling Technology	Cat#2557
PARP; rabbit polyclonal	WB 1:1000	Cell Signaling Technology	Cat#9542
PKB/AKT; rabbit polyclonal	WB 1:1000	Cell Signaling Technology	Cat#9272
RB; mouse monoclonal (clone 4H1)	WB 1:1000	Cell Signaling Technology	Cat#9309
RSK; rabbit monoclonal (clone 32D7)	WB 1:1000	Cell Signaling Technology	Cat#9355
Phospho-ERK1/2 T185 Y187/T202 Y204; rabbit polyclonal	WB 1:1000	Cell Signaling Technology	Cat#9101
Phospho-ERK1/2 T185 Y187/T202 Y204; rabbit monoclonal (clone D13.14.4E)	ICC 1:500	Cell Signaling Technology	Cat#4370
Phospho-ERK1/2 T185 Y187/T202 Y204 Alexa Fluor 488 conjugate; rabbit monoclonal (clone D13.14.4E)	FC 1:100	Cell Signaling Technology	Cat#4344
Phospho-GSK3α/β S21/S9; rabbit polyclonal	WB 1:1000	Cell Signaling Technology	Cat#9331
Phospho-MEK1/2 S217/S221; rabbit polyclonal	WB 1:1000	Cell Signaling Technology	Cat#9121
Phospho-PKB/AKT T308; rabbit monoclonal (clone D25E6)	WB 1:1000	Cell Signaling Technology	Cat#13038
Phospho-PKB/AKT S473; rabbit polyclonal	WB 1:1000	Cell Signaling Technology	Cat#9271
Phospho-RB S795; rabbit polyclonal	WB 1:1000	Cell Signaling Technology	Cat#9301
Phospho-RSK S380; rabbit polyclonal	WB 1:1000	Cell Signaling Technology	Cat#9341
Phospho-RSK S380; rabbit monoclonal (clone D3H11)	ICC 1:200	Cell Signaling Technology	Cat#11989
Phospho-RSK S380; rabbit monoclonal (clone D5D8)	WB 1:1000	Cell Signaling Technology	Cat#12032
Phospho-S6K T389; rabbit polyclonal	WB 1:1000	Cell Signaling Technology	Cat#9205
SNAI1 (Snail); rabbit monoclonal (clone C15D3)	WB 1:1000	Cell Signaling Technology	Cat#3879
SNAI2 (Slug); rabbit monoclonal (clone C19G7)	WB 1:1000	Cell Signaling Technology	Cat#9585
TWIST1; mouse monoclonal (clone Twist2C1a)	WB 1:500	Santa Cruz Biotechnology	Cat#sc-81417
VIM (Vimentin); rabbit monoclonal (clone D21H3)	WB 1:1000; ICC 1:100	Cell Signaling Technology	Cat#5741
ZEB1; rabbit monoclonal (clone D80D3)	WB 1:1000	Cell Signaling Technology	Cat#3396
**Chemicals, peptides, and recombinant proteins**			
5-Fluorouracil		Sigma-Aldrich	Cat#47576; CAS: 51-21-8
GDC-0941 (Pictilisib) PI3K inhibitor		Provided by AstraZeneca	CAS: 957054-30-7

Oxaliplatin	Selleck Chemicals	Cat#S1224;<br>CAS: 61825-94-3
Q-VD-OPh pan-caspase inhibitor	Merck Millipore	Cat#551476;<br>CAS: 1135695-98-5
SCH772984 ERK1/2 inhibitor	Selleck Chemicals	Cat#S7101;<br>CAS: 942183-80-4
Selumetinib (AZD6244) MEK1/2 inhibitor	Provided by AstraZeneca	CAS: 606143-52-6
Custom synthesized quantitative peptide standards, heavy labelled at C-terminal arginine ($^{13}C_6$, $^{15}N_7$):<br><br>ERK1_H_NP GQPFDVGP**R**$_H$<br>ERK2_H_NP GQVFDVGP**R**$_H$<br>ERK1_H_P IADPEHDHTGFLp**T**Ep**Y**VAT**R**$_H$<br>ERK2_H_P VADPDHDHTGFLp**T**Ep**Y**VAT**R**$_H$<br><br>pT, phospho-threonine; pY, phospho-tyrosine. R_H, heavy isotope arginine.	AQUA Ultimate, Sigma-Aldrich	N/A
**Critical commercial assays**		
Nick Translation Kit	Abbott Molecular	Cat#07J00-001
QuantiTect Reverse Transcription Kit	Qiagen	Cat#205311
SYBR Green PCR Master Mix	Thermo Fisher Scientific	Cat#4309155
Ambion Illumina TotalPrep RNA Amplification Kit	Thermo Fisher Scientific	Cat#AMIL1791
HumanHT-12 v4 Expression BeadChip Kit	Illumina	Cat#BD-103-0204
miRNeasy Mini Kit	Qiagen	Cat#217004
ScriptSeq RNA-Seq Library Preparation Kit	Illumina	Cat#SSV21124
AllPrep DNA/RNA/miRNA Universal Kit	Qiagen	Cat#80224
CytoSure HT Genomic DNA Labelling Kit	Oxford Gene Technology	Cat#500040
Agilent Human Genome CGH 2x400k Microarray	Agilent Technologies	N/A
Click-iT EdU Alexa Fluor 647 Flow Cytometry Assay Kit	Thermo Fisher Scientific	Cat#C10424
Click-iT EdU Alexa Fluor 647 HCS Assay Kit	Thermo Fisher Scientific	Cat#C10357
Human ProInflammatory-4 II Tissue Culture Kit	Meso Scale Discovery	Cat#K15025B
Human ProInflammatory 7-Plex Tissue Culture Kit	Meso Scale Discovery	Cat#K15008B
**Deposited data**		
Microarray	Gene Expression Omnibus (https://www.ncbi.nlm.nih.gov/geo/)	GSE120993
RNA sequencing	Gene Expression Omnibus (https://www.ncbi.nlm.nih.gov/geo/)	GSE126109
Array comparative genomic hybridization	Gene Expression Omnibus (https://www.ncbi.nlm.nih.gov/geo/)	GSE126367
**Experimental models: cell lines**		
Human: A549	ATCC	Cat#CCL-185;<br>RRID: CVCL_0023
Human: COLO205	ATCC	Cat#CCL-222;<br>RRID: CVCL_0218
Human: HCT116	Laboratory of Bert Vogelstein	RRID: CVCL_0291
Human: HeLa	ATCC	Cat#CCL-2;<br>RRID: CVCL_0030
Human: HT29	ATCC	Cat#HTB-38;<br>RRID: CVCL_0320
Human: LoVo	Laboratory of Kevin Ryan	RRID: CVCL_0399
Human: SW620	ATCC	Cat#CCL-227;<br>RRID: CVCL_0547
Human: selumetinib-resistant COLO205 (C6244-R) cells	Little et al., 2011	N/A
Human: selumetinib-resistant HCT116 (H6244-R) cells	Little et al., 2011	N/A
Human: selumetinib-resistant HT29 (HT6244-R) cells	Little et al., 2011	N/A
Human: selumetinib-resistant LoVo (L6244-R) cells	Little et al., 2011	N/A
**Experimental models: organisms/strains**		
Mouse, female: NOD.CB17-*Prkdc*^{scid}/J	The Jackson Laboratory	Cat#001303;<br>RRID:IMSR_JAX:001303
Mouse, female: athymic mice (nu/nu:Alpk; AstraZeneca)	AstraZeneca	N/A
**Quantitative PCR primers**		
Human *B2M*<br><br>Fwd: 5′-TGCTGTCTCCATGTTTGATGTAT-3′<br>Rev: 5′-TCTCTGCTCCCCACCTCTAAGT-3′	Sigma-Aldrich custom synthesis	N/A

Human *CDH1*<br><br>Fwd: 5′-CTGGCGTCTGTAGGAAGGCA-3′<br>Rev: 5′-AACAGCAAGAGCAGCAGAATCAG-3′	Sigma-Aldrich custom synthesis	N/A
Human *CDH2*<br><br>Fwd: 5′-ATGAAGAAGGTGGAGGAGAAGAAGA-3′<br>Rev: 5′-ACAGGCTTGATGGCATCAGG-3′	Sigma-Aldrich custom synthesis	N/A
Human *CTGF*<br><br>Fwd: 5′-CGAAGCTGACCTGGAAGAGAAC-3′<br>Rev: 5′-TCGGTATGTCTTCATGCTGGTG-3′	Sigma-Aldrich custom synthesis	N/A
Human *FN1*<br><br>Fwd: 5′-GGACCACACAGAACTATGATGCC-3′<br>Rev: 5′-CCACTGATCTCCAATGCGGTA-3′	Sigma-Aldrich custom synthesis	N/A
Human *RPL13A*<br><br>Fwd: 5′-CCTGGAGGAGAAGAGGAAAGAGA-3′<br>Rev: 5′-TTGAGGACCTCTGTGTATTTGTCAA-3′	Sigma-Aldrich custom synthesis	N/A
Human *SNAI1*<br><br>Fwd: 5′-ACCACTATGCCGCGCTCTT-3′<br>Rev: 5′-GGTCGTAGGGCTGCTGGAA-3′	Sigma-Aldrich custom synthesis	N/A
Human *SNAI2*<br><br>Fwd: 5′-AACTACAGCGAACTGGACACACA-3′<br>Rev: 5′-TGACAGGCATGGAGTAACTCTCA-3′	Sigma-Aldrich custom synthesis	N/A
Human *TWIST1*<br><br>Fwd: 5′-GCAGGACGTGTCCAGCTC-3′<br>Rev: 5′-CTGGCTCTTCCTCGCTGTT-3′	Sigma-Aldrich custom synthesis	N/A
Human *VCAN*<br><br>Fwd: 5′-GTTGGACTGATGGCAGCACA-3′<br>Rev: 5′-GATTACAACACAGTCTTCTCCAGCAG-3′	Sigma-Aldrich custom synthesis	N/A
Human *VIM*<br><br>Fwd: 5′-CAGGAGGAGATGCTTCAGAGAGA-3′<br>Rev: 5′-AAGGTCAAGACGTGCCAGAGA-3′	Sigma-Aldrich custom synthesis	N/A
Human *ZEB1*<br><br>Fwd: 5′-GGCACCTGAAGAGGACCAGA-3′<br>Rev: 5′-ATAGCCTCTATCACAATATGGACAGGT-3′	Sigma-Aldrich custom synthesis	N/A
Human *ZO1*<br><br>Fwd: 5′-GAGGTAGAACGAGGCATCATCC-3′<br>Rev: 5′-AATCTCCAGAAGTCAGCACGGT-3′	Sigma-Aldrich custom synthesis	N/A
Human *ZO3*<br><br>Fwd: 5′-CGAGGAGATGGAGCTGGTGA-3′<br>Rev: 5′-AAGTGAGTGCGGATGTAGAAGGAG-3′	Sigma-Aldrich custom synthesis	N/A
**Sequencing primers**		
Human *CDKN1C* sequencing primers:<br><br>Fwd: 5′-AGAAGAGTCCACCACCGGAC-3′<br>Rev: 5′-CGGACAGCTTCTTGATCGCC-3′	Sigma-Aldrich custom synthesis	N/A
**RNA interference sequences**		
siRNA (siGENOME SMARTpool) against human *SNAI1* targeting sequences:<br>1. ACUCAGAUGUCAAGAAGUA.<br>2. GCAAAUACUGCAACAAGGA.<br>3. GCUCGGACCUUCUCCCGAA.<br>4. GCUUGGGCCAAGUGCCCAA.	Dharmacon, GE Healthcare Life Sciences	Cat#M-010847-00-0005
siRNA (siGENOME SMARTpool) against human *SNAI2* targeting sequences:<br>1. GGACACACAUACAGUGAUU.<br>2. UAAAUACUGUGACAAGGAA.<br>3. GAAUGUCUCUCCUGCACAA.<br>4. GAAUCUGGCUGCUGUGUAG.	Dharmacon, GE Healthcare Life Sciences	Cat#M-017386-00-0005

siRNA (siGENOME SMARTpool) against human *ZEB1* targeting sequences:<br>1. GAACCACCCUUGAAAGUGA.<br>2. GAAGCAGGAUGUACAGUAA.<br>3. AAACUGAACCUGUGGAUUA.<br>4. GAUAGCACUUGUCUUCUGU.	Dharmacon, GE Healthcare Life Sciences	Cat#M-006564-02-0005
siRNA (siGENOME SMARTpool) non-targeting sequences:<br>1. UAAGGCUAUGAAGAGAUAC.<br>2. AUGUAUUGGCCUGUAUUAG.<br>3. AUGAACGUGAAUUGCUCAA.<br>4. UGGUUUACAUGUCGACUAA.	Dharmacon, GE Healthcare Life Sciences	Cat#D-001206-14-05
siRNA against human *CDKN1A* targeting sequence:<br><br>CUGUACUGUUCUGUGUCUU	Eurofins MWG Operon custom synthesis	N/A
**Guide RNA (gRNA) sequences**		
gRNAs to human *CDKN1C*:<br><br>*CDKN1C* gRNA 1: TCCGCAGCACATCCACGATG<br>*CDKN1C* gRNA 2: GTGGGACCTTCCCAGTTACT	ATUM	N/A
**FISH probes**		
BRAF FISH probe PAC clone RP5-1173P7	Laboratory of Paul Edwards	N/A
Chromosome 7 centromere probe	Laboratory of Suet-Feung Chin and Carlos Caldas	N/A
**Recombinant DNA**		
pD1301-AD mammalian Cas9 (double stranded nuclease *Steptococcus pyrogenes* Cas9) genome editing vector	ATUM	N/A
**Software and algorithms**		
GraphPad Prism 5	GraphPad Software	https://www.graphpad.com/scientific-software/prism/
BD CellQuest Pro Software	BD Biosciences	https://www.bd.com/en-uk/products/molecular-diagnostics/cytometric-analysis-products
Adobe Photoshop	Adobe Systems Europe Ltd	http://www.adobe.com/uk/products/photoshop.html
FlowJo	FlowJo LLC	https://www.flowjo.com/solutions/flowjo
CytoVision	Leica Biosystems	http://www.leicabiosystems.com/clinical-microscopy-surgery-radiology/cytogenetics/products/cytovision/
Imaris	Bitplane, Oxford Instruments	http://www.bitplane.com/imaris
Illumina BeadStudio	Illumina	https://emea.illumina.com/
lumi R	Bioconductor	http://www.bioconductor.org/packages//2.7/bioc/html/lumi.html
limma	Bioconductor	http://bioconductor.org/packages/release/bioc/html/limma.html
GSEA software	Broad Institute, Massachusetts Institute of Technology, USA	http://software.broadinstitute.org/gsea/index.jsp
DESeq	Bioconductor	https://bioconductor.org/packages/release/bioc/html/DESeq.html
edgeR	Bioconductor	http://bioconductor.org/packages/release/bioc/html/edgeR.html
TopHat	CCB, Johns Hopkins University, Baltimore, USA	https://ccb.jhu.edu/software/tophat/index.shtml
Genome Analysis Toolkit (GATK)	Broad Institute, Massachusetts Institute of Technology, USA	https://software.broadinstitute.org/gatk/
Agilent Feature Extraction Software	Agilent Technologies	https://www.agilent.com/en/products/genomics-agilent

Nexus Copy Number	BioDiscovery	http://www.biodiscovery.com/nexus-copy-number/
Manual Tracking ImageJ plug in	https://github.com/fiji/Manual_Tracking/releases/tag/Manual_Tracking-2.1.1	https://imagej.net/Manual_Tracking
ibidi GmbH Chemotaxis and Migration Tool	ibidi GmbH	https://ibidi.com/manual-image-analysis/171-chemotaxis-and-migration-tool.html
Skyline	MacCoss Lab Software, University of Washington, USA	https://skyline.ms/project/home/software/Skyline/begin.view

Antibody dilutions are given for flow cytometry (FC), immunocytochemistry (ICC) and Western blotting (WB) as indicated.

Supplementary References

1. Dry, J. R. et al. Transcriptional pathway signatures predict MEK addiction and response to selumetinib (AZD6244). *Cancer Res*. **70**, 2264-2273 (2010).

2. Fridman, A. L. & Tainsky, M. A. Critical pathways in cellular senescence and immortalization revealed by gene expression profiling. *Oncogene* **27**, 5975-5987 (2008).

3. Ikediobi, O. N. et al. Mutation analysis of 24 known cancer genes in the NCI-60 cell line set. *Mol. Cancer Ther.* **5**, 2606-2612 (2006).

4. Berg, K. C. G. et al. Multi-omics of 34 colorectal cancer cell lines - a resource for biomedical studies. *Mol. Cancer* **16**, 116 (2017).

5. Okamoto, A. et al. Mutations and altered expression of p16INK4 in human cancer. *Proc. Natl. Acad. Sci. U.S.A*. **91**, 11045-11049 (1994).
