# Supplementary material for: MEK1/2 inhibitor withdrawal reverses acquired resistance driven by BRAFV600E amplification whereas KRASG13D amplification promotes EMT-chemoresistance
Source: Nat Commun. 2019 May 2;10:2030. doi: 10.1038/s41467-019-09438-w (PMC6497655; doi:10.1038/s41467-019-09438-w)
Supplement: Supplementary file 2 — Description of Additional Supplementary Files [file 41467_2019_9438_MOESM2_ESM.docx]

**Description of Supplementary Files**

**File Name:** Supplementary Data 1.

**Description:** Uncropped Western blot images of Figure 4c. Wild type (p57 WT1) and knockout (p57 KO1, p57 KO2) C6244-R cells generated by CRISPR/Cas9 gene editing with guide RNA (gRNA#2) targeting CDKN1C (encoding p57KIP2) were treated with (+) selumetinib (Sel) or DMSO only (−) for 72 hours. Untransfected COLO205 and C6244-R cells were included as controls. Lysates were then Western blotted with the indicated antibodies and images of the full uncropped membranes are shown.

**File Name:** Supplementary Data 2.

**Description:** Uncropped Western blot images of Figure 8f. HCT116 and H6244-R cells were either left untransfected (UT), transfected with non-targeting (NT) siRNA or transfected with SNAI1-, SNAI2-, or ZEB1-specific siRNA as indicated. 24 hours later cells were treated with 2 μM selumetinib (+) or DMSO only (−) for 48 hours. Lysates were Western bloƩed with the indicated antibodies and images of the full uncropped membranes are shown.
