# Supplementary material for: MEK1/2 inhibitor withdrawal reverses acquired resistance driven by BRAFV600E amplification whereas KRASG13D amplification promotes EMT-chemoresistance
Source: Nat Commun. 2019 May 2;10:2030. doi: 10.1038/s41467-019-09438-w (PMC6497655; doi:10.1038/s41467-019-09438-w)
Supplement: Supplementary file 3 — Supplementary Data 1 [file 41467_2019_9438_MOESM3_ESM.pdf]

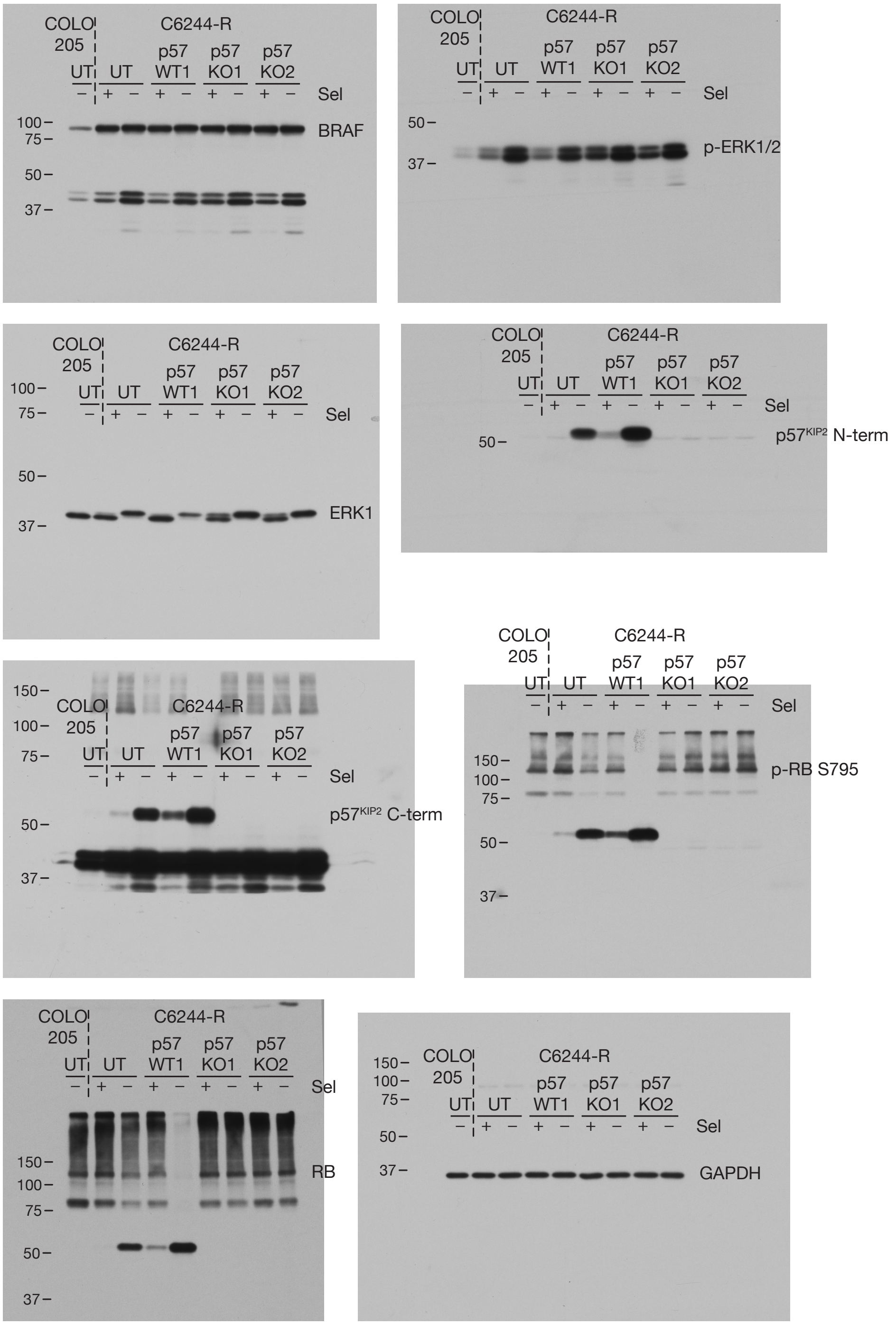

Supplementary Data 1. Uncropped Western blot images of Figure 4c. Wild type (p57 WT1) and knockout (p57 KO1, p57 KO2) C6244-R cells generated by CRISPR/Cas9 gene editing with guide RNA (gRNA#2) targeting *CDKN1C* (encoding p57^{KIP2}) were treated with (+) selumetinib (Sel) or DMSO only (–) for 72 hours. Untransfected COLO205 and C6244-R cells were included as controls. Lysates were then Western blotted with the indicated antibodies and images of the full uncropped membranes are shown.
