# Supplementary material for: MEK1/2 inhibitor withdrawal reverses acquired resistance driven by BRAFV600E amplification whereas KRASG13D amplification promotes EMT-chemoresistance
Source: Nat Commun. 2019 May 2;10:2030. doi: 10.1038/s41467-019-09438-w (PMC6497655; doi:10.1038/s41467-019-09438-w)
Supplement: Supplementary file 4 — Supplementary Data 2 [file 41467_2019_9438_MOESM4_ESM.pdf]

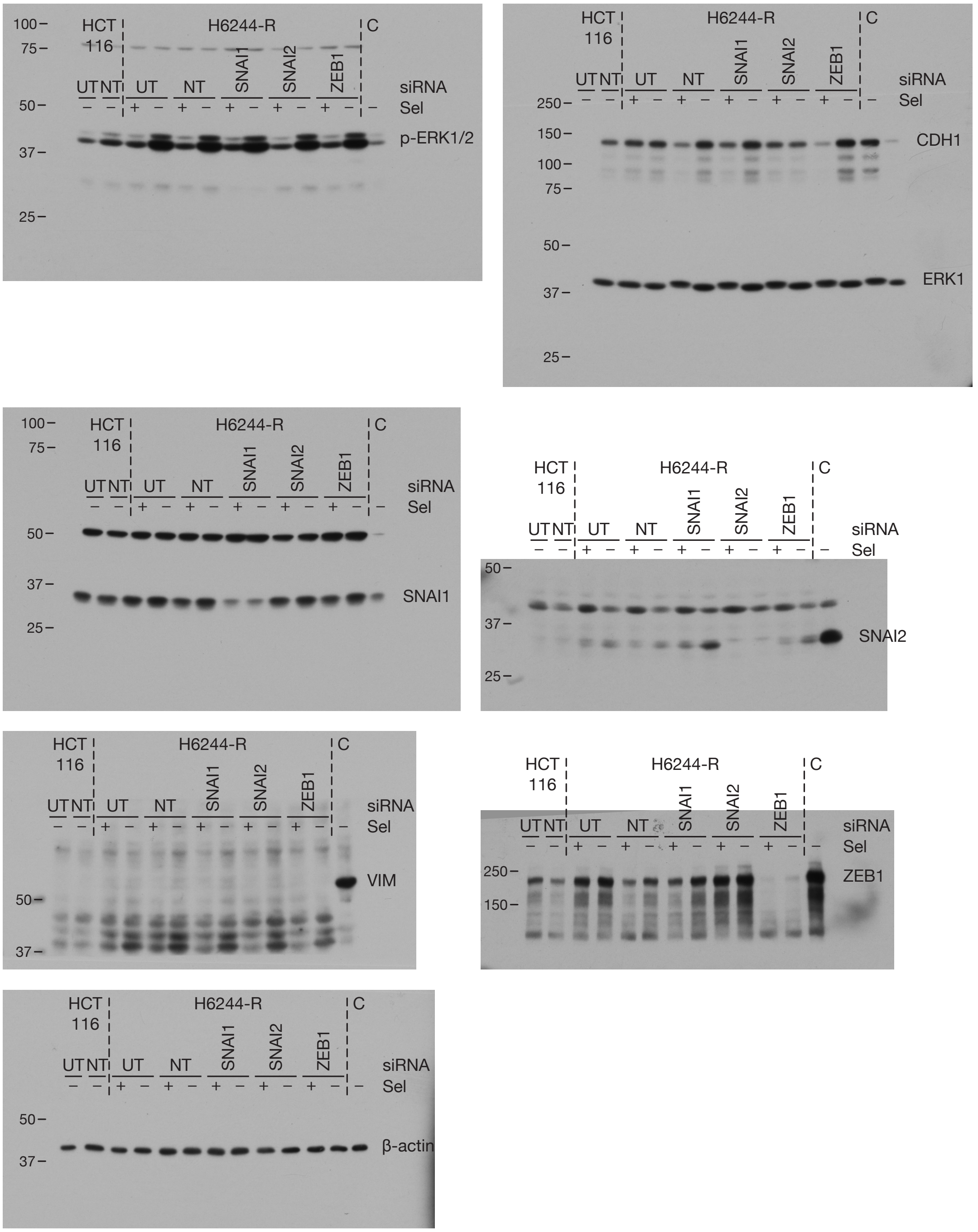

Supplementary Data 2. Uncropped Western blot images of Figure 8f. HCT116 and H6244-R cells were either left untransfected (UT), transfected with non-targeting (NT) siRNA or transfected with SNAI1-, SNAI2-, or ZEB1-specific siRNA as indicated. 24 hours later cells were treated with 2 μM selumetinib (+) or DMSO only (–) for 48 hours. Lysates were Western blotted with the indicated antibodies and images of the full uncropped membranes are shown.
